# A meta-analysis of carbon losses and gains from tropical moist forest degradation and regeneration

**DOI:** 10.1126/sciadv.adz1923

**Published:** 2026-07-03

**Authors:** Viola Heinrich, Amelia Holcomb, Simon Besnard, Daniela Requena Suarez, Susan Cook-Patton, Clément Bourgoin, Robin Chazdon, David A. Gibbs, Flavia Souza Mendes, Iain McNicol, Charlotte Wheeler, Celso H. L. Silva-Junior, Bienvenu H. K. Amani, Jean-Francois Bastin, Timothée Besisa Nguba, Na Chen, Huiying Chen, Philippe Ciais, Ricardo Dalagnol, Xu Dou, Quan Duan, Xueyuan Gao, Ava Nafiseh Goodarzi, Bruno Hérault, Jo House, David M. Lapola, Mengyu Liang, Zekai Meng, Gert-Jan Nabuurs, Erin Poor, Luke Parsons, Johannes Reiche, Stephen Sitch, Ruben Valbuena, Anne-Juul Welsink, Serge Wiltshire, Chao Wu, Yidi Xu, Jinqi Zhao, Luiz Aragão, Martin Herold

**Affiliations:** ^1^ Section 1.4 Remote Sensing and Geoinformatics, GFZ Helmholtz Centre for Geosciences, Potsdam, Germany.; ^2^ School of Geographical Sciences, University of Bristol, Bristol, UK.; ^3^ Department of Computer Science and Technology, University of Cambridge, Cambridge, UK.; ^4^ Department of Geographical Sciences, University of Maryland, College Park, MD 20742, USA.; ^5^ The Nature Conservancy, Arlington, VA 22203, USA.; ^6^ Smithsonian Environmental Research Center, 647 Contees Wharf Road, Edgewater, MD 21037, USA.; ^7^ European Commission, Joint Research Centre (JRC), Ispra, Italy.; ^8^ Forest Research Institute, University of the Sunshine Coast, Sunshine Coast, Queensland, Australia.; ^9^ World Resources Institute, Washington, DC 20002, USA.; ^10^ Planet Labs Germany GmbH, Berlin, Germany.; ^11^ School of Geosciences, University of Edinburgh, Edinburgh EH9 3FF, UK.; ^12^ Revalue, 25 Horsell Rd, London N5 1XL, UK.; ^13^ Department of Plant Science, University of Cambridge, Cambridge, UK.; ^14^ Instituto de Pesquisa Ambiental da Amazônia (IPAM), Brasília 70863-520, Brazil.; ^15^ Programa de Pós-Graduação em Biodiversidade e Conservação (PPGBC), Universidade Federal do Maranhão (UFMA), São Luís 65080-805, Brazil.; ^16^ Université Nangui Abrogoua, Unité de Formation et de Recherches en Sciences de la Nature, Laboratoire d’Écologie et du Développement Durable (LEDD), 02 BP 801 Abidjan 02, Côte d’Ivoire.; ^17^ Teaching and Research Center (TERRA), Gembloux Agro-Bio Tech—Université de Liège, 5030 Gembloux, Belgium.; ^18^ Ecole Régionale Post-Universitaire d’Aménagement et Gestion Intégrés des Forêts et Territoires Tropicaux (ERAIFT), Kinshasa P.O. Box 15.373, Democratic Republic of the Congo.; ^19^ Department of Civil and Environmental Engineering, Massachusetts Institute of Technology (MIT), Cambridge, MA 02139, USA.; ^20^ Department of Earth System Science, Ministry of Education Key Laboratory for Earth System Modeling, Institute for Global Change Studies, Tsinghua University, Beijing 100084, China.; ^21^ Laboratoire des Sciences du Climat et de l’Environnement, LSCE/IPSL, CEA-CNRS-UVSQ, Université Paris-Saclay, 91191 Gif-sur-Yvette, France.; ^22^ CTrees, Pasadena, CA 91105, USA.; ^23^ Department of Ecology and Evolutionary Biology, Princeton University, Princeton, NJ 08544, USA.; ^24^ High Meadows Environmental Institute, Princeton University, Princeton, NJ 08544, USA.; ^25^ CIRAD, UR Forêts et Sociétés, Yamoussoukro, Côte d’Ivoire.; ^26^ Centro de Pesquisas Meteorológicas e Climáticas Aplicadas à Agricultura - CEPAGRI, Universidade Estadual de Campinas - UNICAMP, Campinas 13086-883, Brazil.; ^27^ Department of Earth System Science, Stanford University, Stanford, CA 94305, USA.; ^28^ Wageningen Environmental Research, Wageningen University and Research, Wageningen, Netherlands.; ^29^ Peat to Peak Consulting, Lakewood, CO 80215, USA.; ^30^ Laboratory of Geo-Information Science and Remote Sensing, Wageningen University and Research, Wageningen, Netherlands.; ^31^ Faculty of Environment, Science, and Economy, University of Exeter, Exeter, UK.; ^32^ Swedish University of Agricultural Sciences, Umeå, Sweden.; ^33^ Earth Observation and Geoinformatics Division, National Institute for Space Research (INPE), São José dos Campos, Brazil.; ^34^ University of Potsdam, Potsdam, Germany.

## Abstract

Aboveground carbon (AGC) fluxes from deforestation and subsequent regrowth in tropical moist forest (TMF) are increasingly well characterized, but carbon losses and gains following partial disturbance are uncertain. We synthesized 146 studies quantifying postdisturbance AGC changes relative to undisturbed forests across TMF. Immediate AGC losses (mean ± 1 SD; 2.5 ± 2.3 years after disturbance) following partial anthropogenic disturbances were greatest for forest fires (49 ± 26%), selective logging (34 ± 20%), and edge effects (31 ± 19%). Higher-frequency and -intensity disturbances significantly increased carbon loss. After 20 years of regeneration, AGC stock was higher in recovering degraded forests (41 to 117%) compared to secondary regrowth forests after complete deforestation (1 to 74%), indicating greater regeneration potential when forest structure is preserved. Our compiled database and associated meta-analysis improve accuracy and completeness for carbon inventory reporting and modeling. Substantial AGC losses and gains from distinct degradation and recovery processes are now better characterized, serving as an evidence base for policies to halt degradation and foster recovery for climate mitigation.

## Teaser

Synthesizing 146 studies shows how disturbance type shapes complex degradation and recovery carbon fluxes in tropical forests.

## INTRODUCTION

Tropical moist forests (TMFs) store about 70% (267 Pg C) of global living biomass and historically account for approximately one-third of the global terrestrial carbon sink (*1*). This sink is declining due to deforestation and degradation (*1*). While emissions from large-scale tropical deforestation (complete forest cover removal) are generally well documented and quantified, emissions related to degradation (e.g., caused by understory forest fire, selective logging, edge effects, windthrow, and drought) are difficult to quantify systematically (*2*–*4*). Yet, degradation can result in substantial aboveground carbon (AGC) losses, equivalent to 40 to 280% of deforestation emissions (*5*, *6*). If deforested lands are abandoned and degraded forests remain undisturbed, they can regenerate; at least one-fifth of TMFs are either recovering degraded or secondary regrowth forest (*7*). Secondary forest regrowth after deforestation is relatively well studied across tropical America (*8*–*11*), but less so in Africa and Asia. Recovering degraded forest trajectories remain even less well understood (*12*, *13*). Currently, carbon gains from forest regeneration are represented with inconsistent detail in carbon cycle models and national reporting such as National Greenhouse Gas Inventories (NGHGI) and Forest Reference Emissions Levels (FRELs) (*14*). Furthermore, carbon losses from forest degradation are either inconsistently reported or omitted altogether (*15*–*17*).

Research into postdisturbance carbon losses and gains in TMF has surged, driven in part by the importance of these forests for mitigating climate change and biodiversity loss (*18*, *19*) (table S1). More comprehensive accounting for carbon losses and gains is important for NGHGIs (*20*, *21*), notably in the framework of the Paris Agreement’s Global Stocktake (*22*), as well as for results-based payments (*23*). Despite being halfway through the UN Decade on Ecosystem Restoration, the Forest Declaration Assessment highlighted that progress toward halting degradation in TMF was off-track by 20% in 2023 (*24*).

Advances in satellite remote sensing (RS) since approximately 2015, in combination with field plot and airborne data (fig. S1A), are making it increasingly possible to disaggregate AGC losses from degradation versus deforestation, and AGC gains from recovery (following partial disturbance) versus regrowth (following complete forest clearance). The recent rise in studies about postdisturbance carbon fluxes has created an opportunity for a comprehensive review (*25*) and synthesis across datasets, disentangling the unique contribution of each disturbance driver and regeneration process to the net carbon budget of TMFs.

In this meta-analysis, we (i) outline the proximate drivers of degradation and subsequent recovery; (ii) review the advances in AGC loss and postdegradation gain quantification over the last four decades; (iii) perform a meta-analysis and synthesize existing estimates of loss and gain from TMF degradation and regeneration across disparate data sources, assessing the extent to which they agree at continental and regional scales; and (iv) discuss how research capabilities may support operational and policy-driven efforts, highlighting where future research is needed to fill the remaining gaps.

## CHARACTERIZING FOREST DEGRADATION AND REGENERATION DRIVERS

### Definitions

For this review, “deforestation” refers to the complete loss (clearance) of forest cover and associated AGC. For “degradation,” we follow a definition similar to the Intergovernmental Panel on Climate Change (IPCC): a partial loss in tree cover leading to a medium- to long-term loss of AGC (i.e., carbon loss in “forest land remaining forest land”). In practice, definitions of degradation differ (*26*–*29*), partly because of socio-political context, with the added complexity that disturbances vary in spatial extent, intensity, frequency, and severity, and can interact and co-occur with each other (*30*). “Secondary regrowth forest” or “regrowth” refers to natural AGC gain after deforestation, while “recovering degraded forest” or “recovery” refers to natural gain after degradation. Together, we refer to both categories of AGC gains as “natural regeneration” (*7*).

### Drivers

**Fig. 1.**
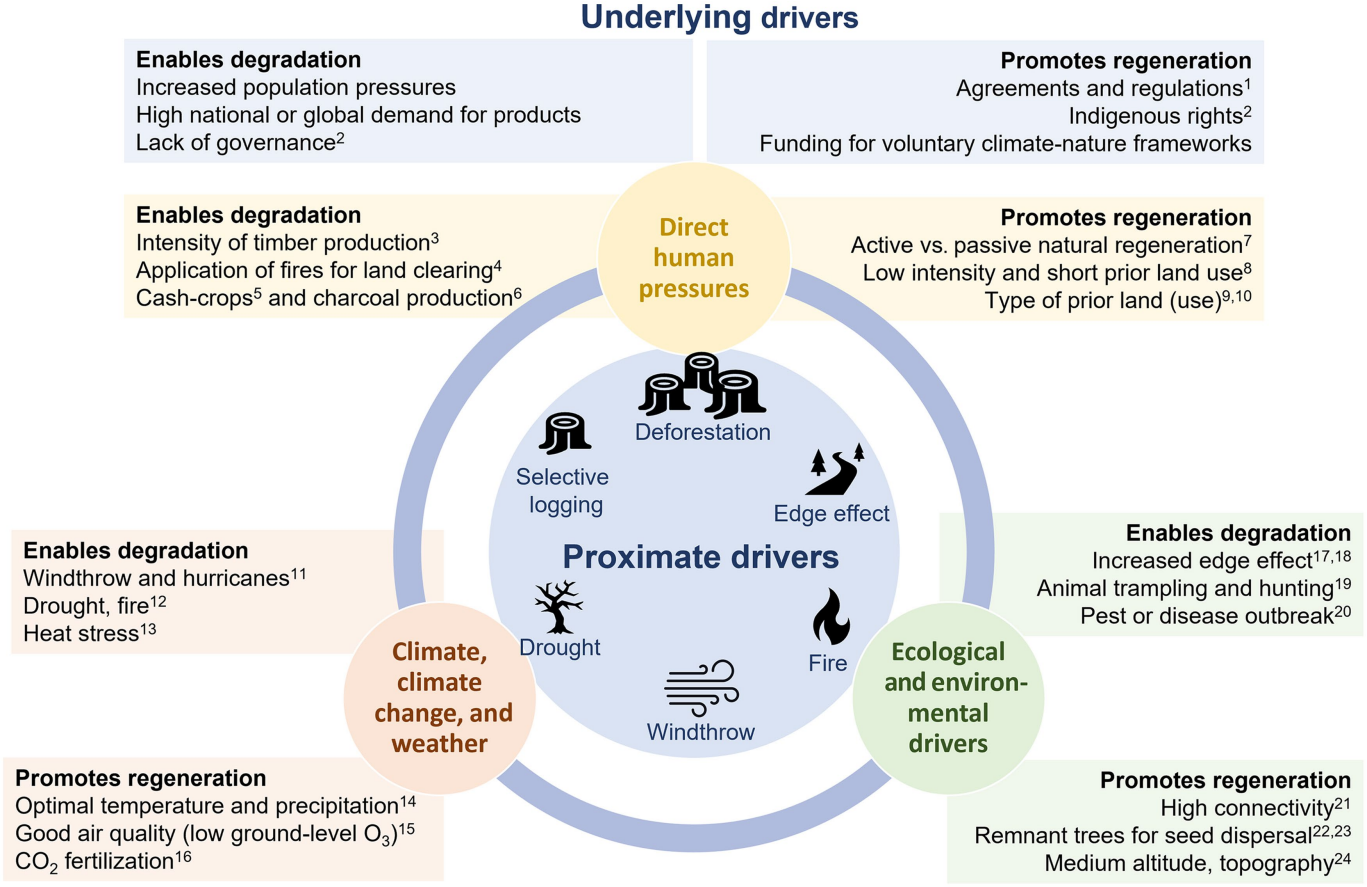
Summary of the main drivers that enable degradation and promote regeneration, respectively. The proximate drivers are central to distinguishing degradation from deforestation and any potential regeneration that will occur thereafter. Numbers indicate the associated references to which the point refers to (different from how they appear in Reference Summary), they are as follows: (1) See table S1; (2) Baragwanath *et al.* (*147*); (3) Imai *et al.* (*148*); (4) Balch *et al.* (*149*); (5) Barima *et al.* (*150*); (6) Liang *et al.* (*90*); (7) Philipson *et al.* (*53*); (8) Jakovac *et al.* (*151*); (9) N’Guessan *et al.* (*51*); (10) Fearnside *et al.* (*61*); (11) Magnabosco Marra *et al.* (*152*); (12) Brando *et al.* (*153*); (13) Docherty *et al.* (*134*); (14) Poorter *et al.* (*10*); (15) Cheesman *et al.* (*65*); (16) Friedlingstein *et al.* (*36*); (17) Silva Junior *et al.* (*15*); (18) Traoré *et al.* (*154*); (19) Quisehuatl-Medina *et al.* (*155*); (20) van Lierop *et al.* (*156*); (21) Smith *et al.* (*55*); (22) Estrada-Villegas *et al.* (*157*); (23) Amani *et al.* (*56*); (24) Davidson *et al.* (*59*); (25) Su *et al.* (*58*). This figure builds and expands on the work of Lapola *et al.* (*30*).

**Fig. 2.**
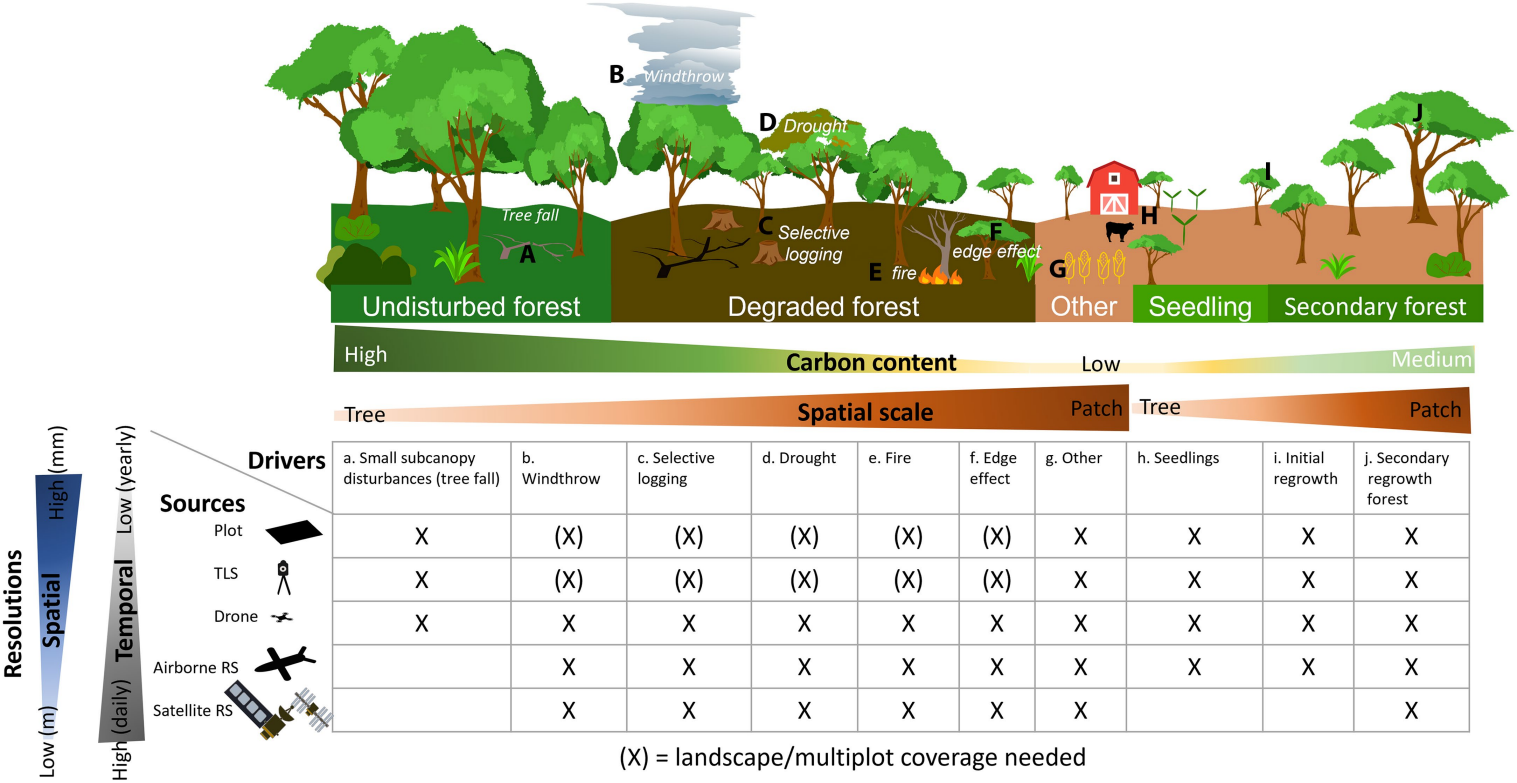
Conceptual diagram of changing forest structure dynamics, the drivers of disturbance, and the data sources available to measure them. The different stages are as follows: (a) an undisturbed forest with small subcanopy disturbance; a degraded forest, which may experience disturbances such as (b) windthrow, (c) selective logging, (d) drought, (e) fire, or a combination of multiple disturbances at various intensities; (f) forest edge effect where multiple disturbances may also interact; (g) other lands that experienced deforestation, but where tree remnants remain common in the landscape; (h) the regrowth of seedlings; (i) the development of small forest structures; (j) larger regenerating forest structures. The colored bars below illustrate the aboveground woody biomass carbon (green-yellow) across forest stages, together with a simplified representation of the approximate spatial scales at which disturbance drivers affect forest area change during degradation, deforestation, and regrowth (reds-browns). In the table of data sources, the crosses indicate the data sources [field plot data, terrestrial laser scanning, drone, airborne remote sensing (RS), and satellite-RS] currently used to measure carbon change across these forest structure dynamics, along with their approximate spatial (blues) and temporal (grays) resolutions. In the case of ground-based measurements, the ability to reliably measure changes may require landscape or multiplot coverage. Illustration credit: T. Rosan.

In TMF, the five main proximate drivers of degradation with quantified AGC losses are as follows: forest fires, selective logging, edge effects, drought-induced mortality (*31*), and windthrow (Figs. 1 and 2) (*30*, *32*–*35*). Distinguishing between direct anthropogenic and natural disturbances is key for the Global Carbon Budget (*36*) and other carbon accounting, such as NGHGI. In practice, disentangling anthropogenic and natural degradation is challenging given their interaction and feedback cycles (*37*). For example, in the fire-sensitive Amazon, human-induced fires are exacerbated in size and intensity by anthropogenic climate change, forest conditions, and fragmentation patterns (*38*). Selective logging (an anthropogenic disturbance) can lead to drier microclimates and increase dry fuel load, making forest fires more likely (*39*). Studies in the 1990s showed that forest fragmentation (an anthropogenic disturbance) leads to greater tree mortality within 100 m of the forest edge than interior trees due to microclimate changes and elevated wind and light exposure. This is known as the “edge effect” (*40*) (Fig. 2) and is understood to reach up to 1.5 km into the forest interior (*41*). Land-use changes and natural climatic oscillations, such as oceanic temperature (*42*), can lower precipitation and exacerbate drought (*43*, *44*), which can then increase tree mortality and fire incidence (*45*). Windthrows are natural disturbances (Fig. 2), yet may also become more frequent and severe with anthropogenic climate change (*46*). The sensitivity of TMF to unique and interacting drivers can lead to different pathways of carbon loss and subsequent gains, which can now be quantified according to their underlying mechanisms, reducing uncertainties in overall carbon fluxes.

Unlike the direct drivers of degradation, which are generally known and can be grouped accordingly, studies concerning drivers of AGC gain often consider multiple environmental, climatic, and human interventions (Fig. 1), making it difficult to directly compare and identify the most influential drivers. Nevertheless, time since disturbance and forest age are generally considered to be dominant drivers influencing AGC gain in regenerating forests (*11*, *47*). Secondary regrowth forests take at least 50 to 80 years to approach the structure of an old-growth rainforest (*48*) and considerably longer to recover their seed bank and floristic composition (*49*). Slowing or accelerating the regeneration process is linked to other direct drivers, such as (i) the characteristics of the disturbance (type, spatial extent, intensity, and frequency) (*32*, *50**–**52*); (ii) the type of regeneration taking place, e.g., assisted versus passive natural regeneration (*53*), management or silvicultural treatments (*54*); (iii) biological conditions such as the proximity to forests (*55*), seed availability, and presence of remnant trees (*56*); (iv) climatic conditions, such as precipitation and drought (*57*); and (v) environmental conditions such as altitude, topography (*58*), and soil type (*59*, *60*) (Fig. 1).

Prior land use before land abandonment and subsequent regrowth strongly influences AGC gain; field studies show slower regrowth after rice than yam cultivation in West Africa (*51*) and 38% higher AGC gain on former croplands than pastures in the Brazilian Amazon, linked to larger seedbanks and lower burn rates (*61*, *62*). Regrowth may also be accelerated by the decision to retain remnant trees within cultivated fields, which maintains soil fertility, buffers microclimates, and acts as seed sources for forest regeneration (*56*) (Fig. 1). Silvicultural treatments after selective logging such as thinning and liana cutting have both shown to increase rates of recovery after degradation (*54*, *63*). Studies integrating multiple field and satellite-RS data sources show a broad-scale influence of key climate variables on AGC gain (*9*–*11*). Research in tropical America shows faster AGC gain in regions with high precipitation, low water deficit (*10*, *11*), and low maximum temperature (*12*, *64*). Air pollution disproportionately affects secondary regrowth forests given their proximity to anthropogenic frontiers, with high levels of ozone reducing net primary productivity of trees (*65*). Soil nutrients and conditions also influence AGC gain, although divergent results across datasets limit general conclusions (*11*, *59*, *66*, *67*).

## FROM FIELD TO SATELLITE: CURRENT APPROACHES TO MEASURING CARBON LOSSES AND GAINS

AGC stock cannot directly be measured without destructive methods. Instead, it relies on applying allometric equations and wood density values to convert measurable variables (e.g., tree/forest height, diameter, and species) into estimates of aboveground biomass (*68*). AGC is then inferred from aboveground biomass by assuming a carbon-to-water ratio in living biomass between 0.456 and 0.5, depending on the ecoregion (*69*).

**Fig. 3.**
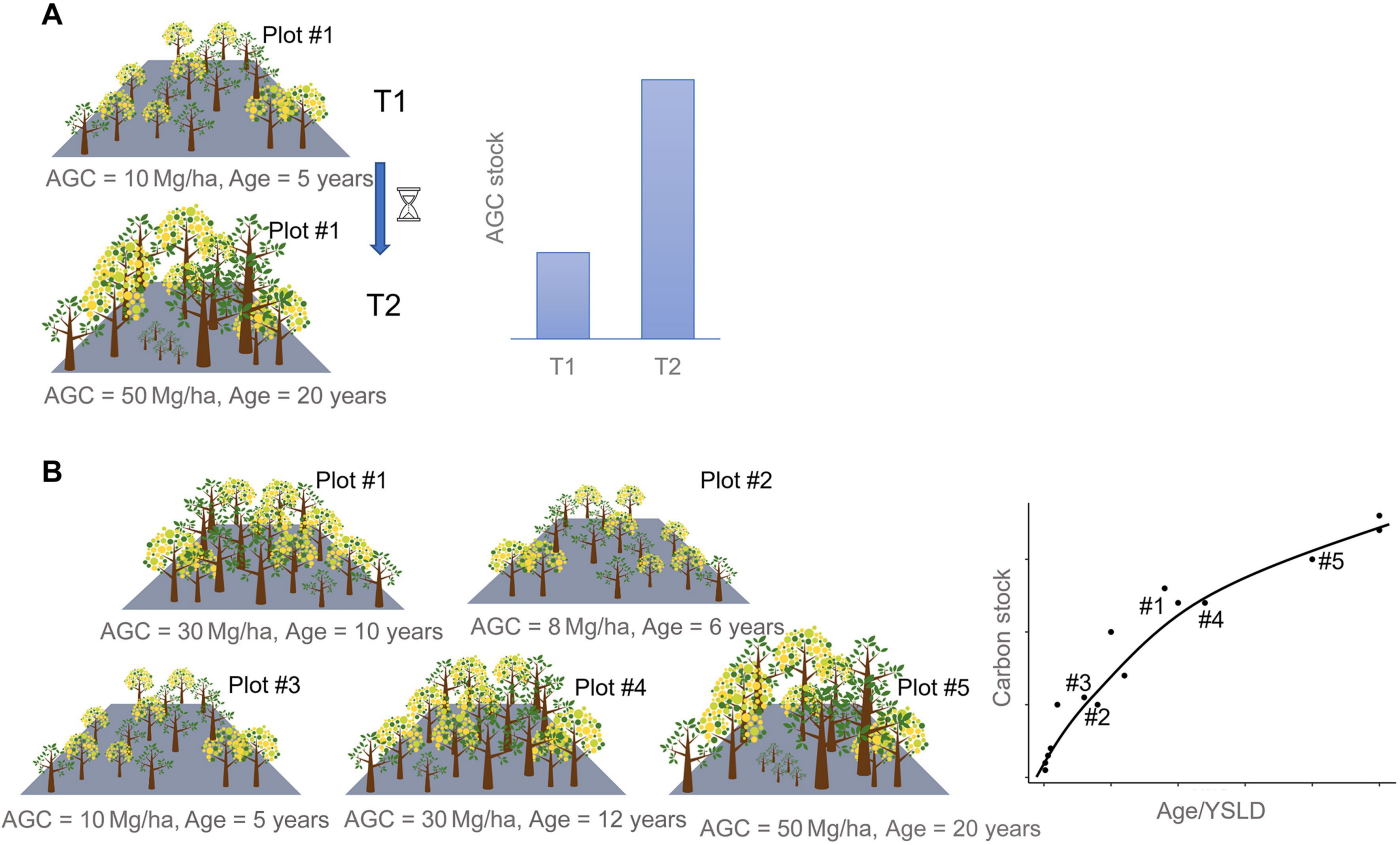
Illustration of the two approaches for measuring aboveground carbon (AGC) losses and gains. (**A**) Remeasuring plots/satellite pixels of forest at two distinct time points. (**B**) Measuring multiple plots of distinct forest structure properties [AGC, age/years since last disturbance (YSLD)] at the same/similar time, and infer temporal changes in a space-for-time-substitution (chronosequences) approach.

AGC losses and year-on-year AGC gains can be summarized by “emission” and “removal” factors, typically expressed as a mean loss (Mg C ha^−1^) in the case of acute degradation and deforestation events or a mean annual rate of gain (Mg C ha^−1^ year^−1^) in the case of ongoing growth. These factors are applied to activity data (e.g., area information of deforestation, degradation, regrowth, and recovery) to estimate total greenhouse gas (GHG) emissions to the atmosphere or GHG removals from the atmosphere by forest sinks (Box 1 and Fig. 3). While the uncertainties in activity area data are relatively well accounted for in best practice guidance (*70*, *71*), emissions and removal factors are critical points that introduce substantial uncertainty into GHG accounting methods. Here, we review the four primary data sources from which emissions and removal factors are derived: field sites, airborne-RS, satellite-RS, and “data integration,” an integration of multiple field data sources across large geographical extents.

Box 1. **Summary of the methods available for measuring forest carbon losses and gains.**

**1) Repeat measurements:**

Carbon losses and gains are measured by comparing a single site/remote sensing pixel cluster at multiple points in time (T1 and T2) (Fig. 3A). This approach provides an estimate of net carbon change and can be used to derive emissions and removal factors for other sites with similar properties.

**2) Chronosequence/Space-for-time substitution approach:**

Numerous plots of different ages or at different stages of disturbance and recovery are measured/observed with field or RS data at the same (or at a similar) time (Fig. 3B). Temporal patterns are modeled from the heterogeneity across the sites and used to provide carbon loss or gain estimates. Gross growth and mortality are more difficult to assess in this manner, i.e., only a net stock difference is assessed. One key limitation to the chronosequence approach is the assumption that spatial differences in AGC are purely due to differences in forest age. However, older and younger forests often occur in different landscapes, as land use practices, forest fragmentation, and environmental and climate conditions change over time, and may not be directly comparable.

### Field plot data: Conventional forest inventory and terrestrial laser scanning

Field data, particularly repeat measurements of permanent plots, are foundational to estimating AGC losses and gains (Box 1 and Fig. 3). For example, they have been used to assess temporal changes in Amazonian secondary succession (*72*, *73*). For operational applications, repeat measurements are constrained by data availability and methodological limitations. For example, most tropical forest countries have conducted only a single national forest inventory (NFI) (Fig. 2A) (*74*), precluding the direct calculation of carbon stock changes. Where remeasurements exist, field protocols and long census intervals tend to systematically underestimate the impacts of degradation and young forest regeneration (*75*, *76*). Furthermore, NFI protocols often omit small trees and highly degraded and recently deforested regions (*76*, *77*), and marking trees for remeasurement may affect human behavior in those areas, leading to biased samples (*72*). Terrestrial laser scanning (TLS) can better capture tree structure and diversity, improving allometry estimates in highly diverse tropical forests using nondestructive methods (*26*). However, TLS remains a time-consuming and difficult task, especially in remote and large-scale areas.

### Field data integration

When repeated measurements are not feasible, a collection of numerous field data plots across large geographical areas can offer landscape-scale understanding of degradation-recovery processes not captured in single field plots (Fig. 2). A chronosequence or space-for-time substitution approach is often used (*69*, *78*, *79*) (Box 1 and Fig. 3), estimating AGC losses and gains at regional (*9*, *51*) or continental scales (*48*, *79*). This approach has greater temporal resolution than infrequent repeat measurements, but at the expense of spatial detail. Chronosequence approaches require systematic spatial information on forest type and age and are commonly used in ecological research and to inform policy. For example, the tier 1 removal factors for (sub)tropical young secondary (≤20 years) and old secondary forest (>20 years) in the 2019 Refinement to the 2006 IPCC Guidelines for NGHGI Good Practice Guidance (GPG) were based on a collection of field data across forest age gradients (*69*, *79*).

### Airborne-RS

Airborne laser scanning (ALS) has increasingly been used to quantify AGC losses and gains, offering high spatial resolution information to capture a range of degradation-regrowth dynamics at regional to (sub)national scales (Fig. 2). Tropical regions rich in ALS data include the Amazon, where repeat ALS measurements have quantified carbon losses for different degradation drivers (*80*), based on indirect measures of AGC such as canopy height. As ALS surveys are time and resource intensive, repeat coverage remains limited; globally, only about 19% of scanned areas have been surveyed more than once. The scientific community is now working toward a harmonized global ALS Atlas to improve integration across datasets (*81*).

### Satellite-RS

Like ALS, Satellite-RS datasets provide indirect biomass estimates inferred from vegetation spectral and structural signals (e.g., greenness, canopy height, cover, and water content) (*82*). Satellite-based mapping of forest carbon dynamics has advanced since 2020 (fig. S1A) (*83*), especially in dense tropical forests using optical and radar satellites (*13*, *15*, *84**–**86*) (Fig. 2). Long-term (e.g., Landsat) and newer [e.g., Sentinel-1, Sentinel-2, or Global Ecosystem Dynamics Investigation (GEDI)] missions enhance our capacity to track carbon gain and loss in forests (*87*, *88*).

Satellite-based repeat measurements of AGC have been used to quantify long-term degradation across African woodlands (*89*) and forests (*90*). Meanwhile, coincident repeat GEDI shots has enabled estimates of immediate carbon losses following degradation events, although its utility for detecting subtle regrowth remains largely unvalidated (*87*). Instead, chronosequence (Box 1 and Fig. 3) approaches have successfully been used in satellite-RS approaches to assess AGC gains in recovering degraded and secondary regrowth forest (*12*, *91*, *92*). The approach relies on satellite-based information of AGC (*82*) and temporal data of forest types and ages (e.g., degraded, secondary, undisturbed forest) (*84*, *93*). This approach can also be used for degradation AGC losses, derived by comparing AGC in recently degraded regions and undisturbed forest (*41*, *45*, *94*), or from the *y* intercept of a space-for-time recovery model (*12*, *90*). Overall, satellite-RS can now more comprehensively capture AGC dynamics in degraded and recovering tropical forests over the last four decades, serving as a useful tool to complement field-site and data-integration datasets (*92*).

Nevertheless, satellite-derived biomass products also have uncertainties and internal inconsistencies within products across years that make it difficult to calculate AGC changes directly from successive maps (*95*, *96*). Compounding these difficulties, degradation and its associated recovery occur across a range of spatial extents, sometimes smaller than what can be detected by some of the best available biomass products (Fig. 2) (*97*). Integrating field data and satellite data can help to overcome these issues, offering unique insights, such as providing highly accurate estimates of recovery in degraded tropical forest in Peru (*98*). Satellite-based activity data have been incorporated into national reporting (*74*), expanding our understanding of when degradation and recovery occur outside the limited areas captured by field data (*34*, *85*).

Overall, each data source offers complementary information. Wall-to-wall satellite-RS maps of AGC provide comprehensive coverage (*99*), spatially filling gaps where ground and ALS data are lacking. Field and TLS data provide highly accurate information on AGC stock, supporting the calibration/validation of satellite-derived AGC estimates, which are affected by systematic biases, including overestimation in low-biomass regions and saturation-driven underestimation in high-biomass regions (*95*).

## META-ANALYSIS OF AGC LOSS AND GAIN RATES

Here, we present a meta-analysis based on our compilation of 146 peer-reviewed research studies (1988 to January 2026) (see Methods: Meta-analysis) that estimate AGC losses and gains in TMF. We categorize studies based on the region studied (tropical America, Africa, or Asia) and the data source used [field sites, satellites, airborne-RS, or data integration (the integration of multiple field data sources across large geographical extents)] (fig. S1 and table S6). To maintain consistency across drivers, data sources, spatial extents (local, regional, subnational, national, and continental), and regions (America, Africa, and Asia), we normalized the data by reporting per-hectare carbon losses and gains (Mg C ha^−1^) relative to AGC density in undisturbed forest as the percentage AGC loss/gain (mean ± 1 SD). This allowed us to synthesize conclusions across studies, identify knowledge gaps, and guide end-users on applicable AGC loss and gain rates to be used in carbon reporting and modeling.

### Carbon losses from deforestation and degradation

**Fig. 4.**
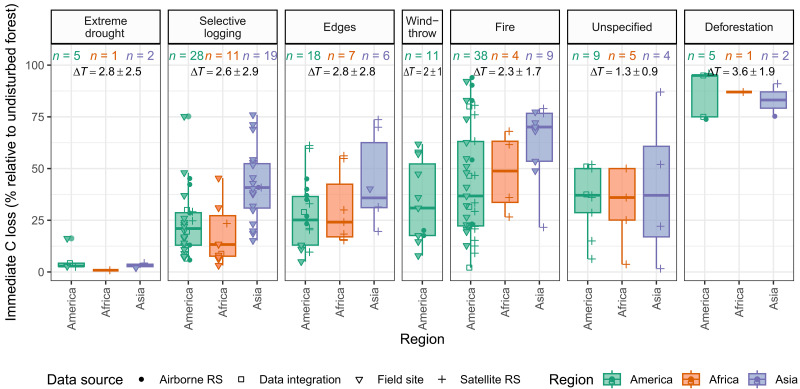
The immediate carbon (C) loss (as percentage) for different disturbance types compared to nearby undisturbed or prior forest state. Values are equivalent to % loss per hectare. The different disturbance types that can cause deforestation and degradation and the carbon losses as identified by different data sources. Δ*T* refers to the average number of years between the disturbance event and when the measurements for the study were taken, ± is 1 SD. *N* is the number of datapoints. We acknowledge that there may be overlaps in the datasets used by each study, especially the data integration studies.

Reported carbon losses from degradation ranged from 0.8 to 96% of predisturbance/undisturbed stocks (Fig. 4 and fig. S2), with measurements taken on average 2.5 (±2.3) years postdisturbance (fig. S8). AGC loss estimates across data sources (field site, airborne-RS, satellite-RS, and data integration) showed no significant difference [Tukey’s honestly significant difference (HSD); *P* > 0.05, fig. S3], but large variability within disturbance drivers (Fig. 4 and fig. S6). Because of variability and the limited number of studies per region, most between-group comparisons of carbon losses by disturbance type were not statistically significant, but may show a large effect size (fig. S2).

For studies estimating emissions from deforestation, in which carbon losses were not 100%, the average loss was 86 ± 9.8% (Fig. 4 and table S2) and always significantly higher than drivers of partial disturbance (fig. S2). Extreme drought had consistently low AGC loss estimates (mean: 5 ± 5.6%; table S2), significantly lower than forest fires (mean: 48 ± 25.5%) and conventional selective logging (mean: 39 ± 19.2%; table S2) (Tukey’s HSD; *P* < 0.05; Hedge’s *g* > 0.8). On the basis of mixed-effect models that individually encompass data type, spatial extent, and degradation type, we saw that “local” extent studies reported stronger negative effects (*P* < 0.1) than across other extents (*P* > 0.1), but the disturbance drivers had the strongest and most significant impact on AGC loss (*P* < 0.0001) (table S5). This suggests that our reported means are widely applicable across regional to continental extents, when no locally relevant values are available (see Methods: Meta-analysis).

**Fig. 5.**
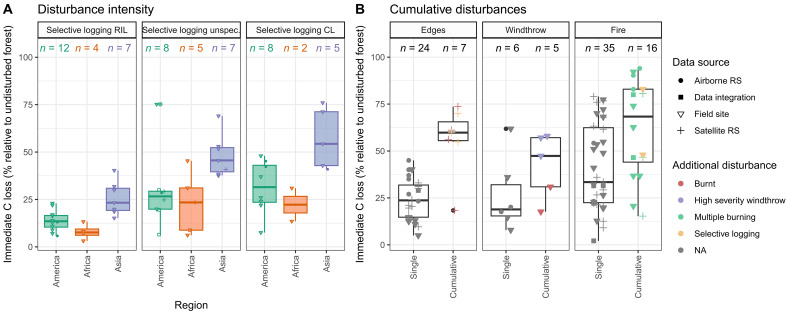
The immediate carbon (C) loss (as percentage) considering different intensities and cumulative effects of disturbances. Values are equivalent to % loss per hectare. (**A**) The carbon deficits across reduced impact logging (RIL), conventional selective logging (CL), and where the type of selective logging was unspecified split up according to the three major tropical regions (colors) and by data source type (shapes). (**B**) The impact of cumulative disturbances, which includes repeat disturbances, where the study observed two or more disturbances of the same type over multiple years at the same location, and co-occurring disturbances, which refers to different disturbance types occurring at the same time or space.

High-intensity and repeated (cumulative) disturbances were associated with higher carbon losses per hectare (Fig. 5 and fig. S8) (Tukey HSD test, *P* < 0.05, figs. S3 and S5). The meta-analysis showed that AGC losses were significantly higher from conventional selective logging (mean: 39 ± 20%) than lower-intensity reduced impact logging (mean: 16 ± 9%) across the three regions (Tukey’s HSD; *P* < 0.05; Hedge’s *g* > 0.8) (Fig. 5A and table S2). Furthermore, AGC losses from selective logging (all types) were consistently the highest in Asia, followed by America and Africa (table S2). On an area basis, cumulative disturbances generally produced significantly greater AGC losses than single disturbances (fig. S5 and table S3). Degraded forests at edges that experienced additional disturbances, such as burning or selective logging, resulted in mean 56 ± 18% AGC loss, compared to edge effects alone (mean: 24 ± 24% AGC loss) (Fig. 5B and table S3). Single forest fires resulted in immediate AGC losses of 41 ± 24%, compared to 62 ± 26% due to repeated fires (table S2).

### Carbon gains from regeneration

**Fig. 6.**
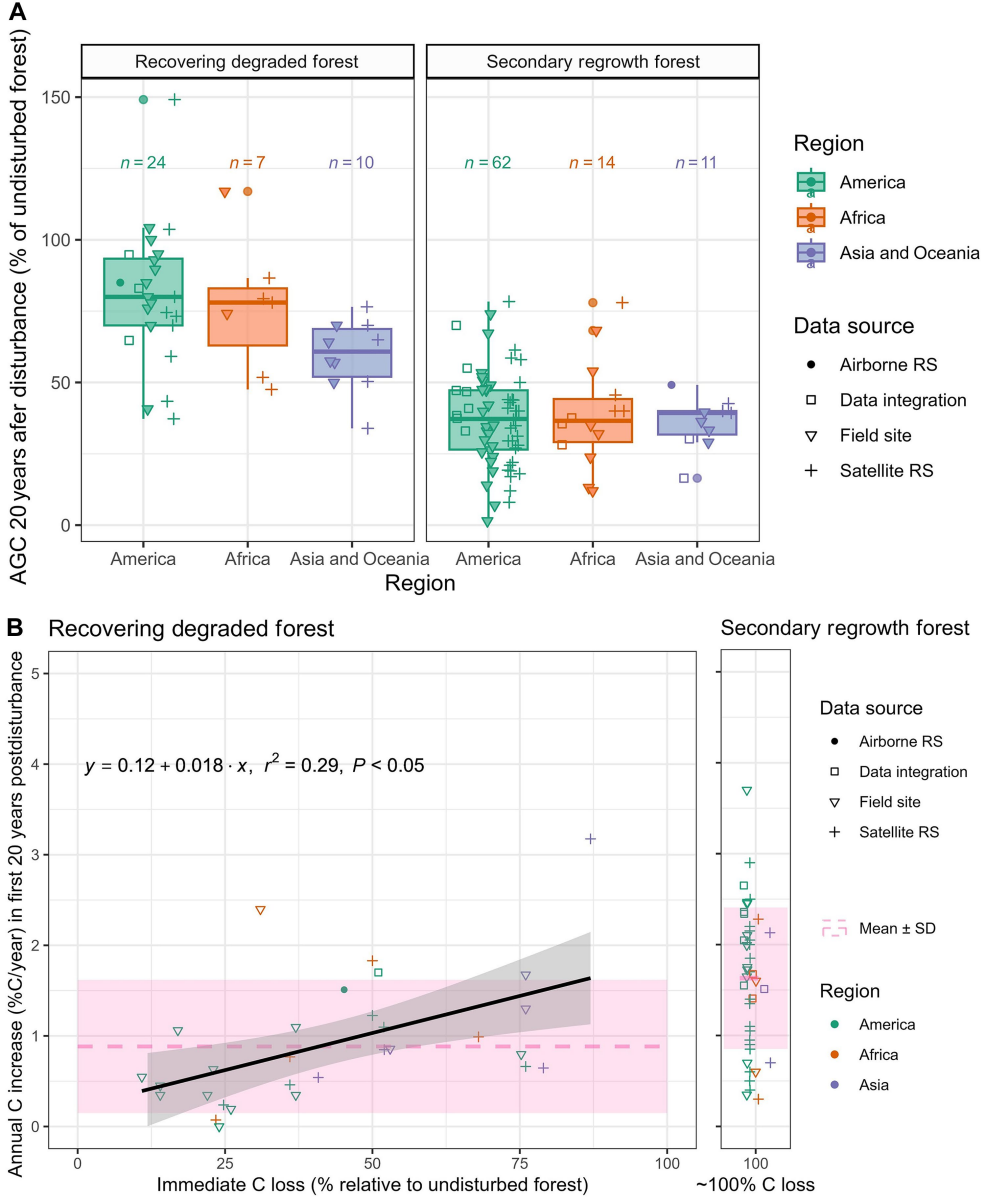
Relative aboveground carbon (AGC) stocks and gains after disturbance in degraded and secondary forest across the tropics. (**A**) Boxplots of the relative aboveground carbon stock after 20 years after disturbance ceased. Points represent individual inputs from different studies according to the data source (shapes), with *n* = indicating the total number of inputs for each region and forest type. (**B**) The relative carbon losses (*x* axis) and associated annual gains (*y* axis) based on studies that captured both disturbance and regeneration in recovering degraded and secondary regrowth forest. Pink dashed horizontal lines are the mean and 1 SD of annual C increase (as %); the black line is the linear regression (and associated equation shown) assuming an *x* ~ *y* relationship. In both (A) and (B), values are relative to prior standing forest or nearby undisturbed forest state. Note that for secondary forest, the loss may not be 100% but is displayed as such here for presentation purposes only.

For AGC gains, we found high variability across studies, reflecting the spatiotemporal complexity of forest regeneration (Fig. 6A) and limited data across Africa, Asia, and Oceania, compared to America. Twenty years after the last disturbance, AGC stocks per hectare were, on average, significantly higher in recovering degraded forest (mean: 75 ± 23% relative to undisturbed AGC density) than in secondary regrowth forest (mean: 38 ± 16%) (Fig. 6A; Student’s *t* test *P* < 0.05). The difference is not unusual given that more carbon stock remains after degradation than deforestation (Fig. 4). In the first 20 years after disturbance, percentage rates of accumulation were faster in secondary regrowth forest (+1.6 ± 0.8% year^−1^) compared to recovering degraded forest (+0.9 ± 0.7% year^−1^) (Fig. 6B), with no significant impact of the spatial extent at which the studies took place (table S6).

While relative rates enable direct comparisons across large regions and data sources, absolute estimates of carbon gains (Mg C ha^−1^ year^−1^) are more directly applicable for reporting frameworks. Across the tropics, the mean absolute AGC accumulation rate was lower in recovering degraded forests (1.7 to 2.8 Mg C ha^−1^ year^−1^) than secondary regrowth forests (2.2 to 2.6 Mg C ha^−1^ year^−1^) (figs. S9 and 14 and table S4), but this difference was not statistically significant (Student’s *t* test *P* > 0.05). Furthermore, relatively few studies were available for recovery of carbon in degraded forest recovery, limiting comparisons. One might expect rates to be lower in degraded forests, potentially due to ongoing tree mortality after the initial disturbance, and greater competition among remaining standing trees (*98*, *100*), which grow slower than new trees after deforestation (*12*). Across the studies, there was consensus that absolute accumulation rates in young regenerating forest (≤20 years) are significantly higher than in older forest ages (>20 years) (*P* < 0.05; fig. S9), driven by fast-growing, open pioneer species, but rates may also be influenced by whether the study used chronosequence or remeasurement (*101*).

**Fig. 7.**
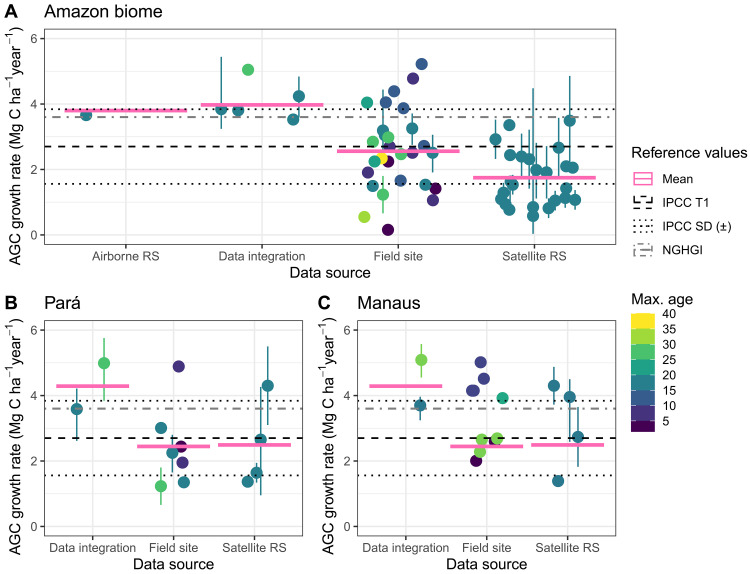
Regional and locally relevant mean absolute aboveground carbon (AGC) accumulation rates (in Mg C ha^−1^ year^−1^) for young (≤20 years) secondary regrowth forest. Studies are from within (**A**) the Amazon and, more specifically, (**B**) Para and (**C**) Manaus. Max. age refers to the maximum included in the respective studies; note that the Max. age may be higher than 20 years if the growth rate was described to be linear in the study. For the field-site data, we determined the location of the data based on the information given in the site descriptions. For the satellite data and data integration studies, we used region-specific growth curves or clipped spatially explicit data to within 50 km of field-site locations to extract the mean growth rate. The cross bar for each data source is the mean for the respective data sources. Error bars denote the uncertainties given in each study, when available. We acknowledge that there may be overlaps in the datasets used by each study, especially the data integration and field-site studies.

In addition, we conducted a detailed analysis of absolute carbon gains, for which we had substantial data, namely, in young Amazonian secondary regrowth forests (Fig. 7 and fig. S9). We compared data sources to ascertain whether rates from different methodologies agree, despite potential variability in the regrowth ages used to derive the growth rates, especially in the field data (Fig. 7 and fig. S16). At the Amazon biome scale, the average of all data sources fell within the SD of the IPCC tier 1 estimate for young secondary regrowth forests in North and South American tropical rainforests (Fig. 7A) (average: 2.69 ± 1.14 Mg C ha^−1^ year^−1^). Satellite-derived AGC gain rates were consistently lower (average: 1.75 Mg C ha^−1^ year^−1^) compared to those measured in field sites (average: 2.56 Mg C ha^−1^ year^−1^) and data integration (average: 3.97 Mg C ha^−1^ year^−1^) (Fig. 7A and fig. S9). We also compared field-site and data-integration regrowth rates from the State of Pará (Brazil) and around the city of Manaus with regionally derived satellite estimates (Fig. 7, B and C). The average secondary regrowth rates converged for field- and satellite-derived estimates, and the greatest difference was for data integration (Tukey’s HSD test *P* < 0.05) (fig. S12).

## OPPORTUNITIES AND GAPS FOR USING EXISTING CARBON LOSS AND GAIN DATA IN TROPICAL FOREST DYNAMICS

### Deforestation and degradation

**Fig. 8.**
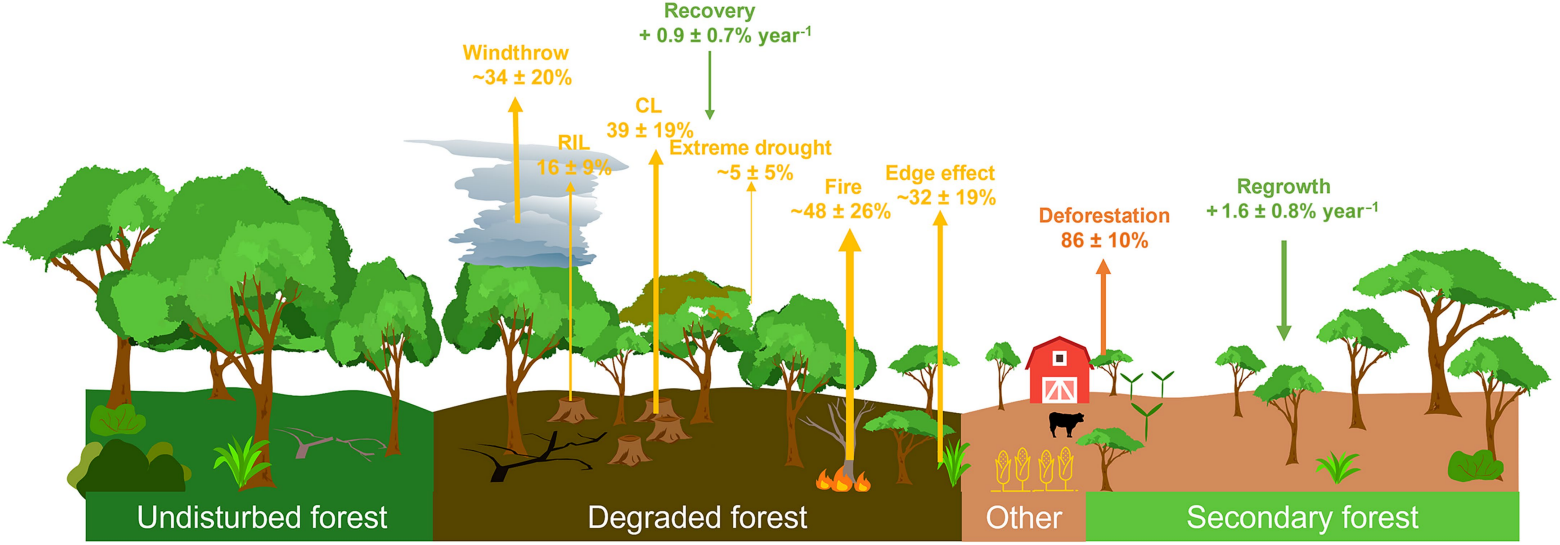
Summary of the carbon losses due to degradation and carbon gains in recovering degraded forest and secondary regrowth forest. Conceptual diagram showing the average relative losses and gains in Aboveground Carbon (as a percentage) compiled from our synthesis, as well as their associated SD. For recovering degraded forests and secondary regrowth forests, the percentage gain is relevant only for the first 20 years of regeneration. Illustration credit: T. Rosan.

Our meta-analysis shows general agreement in percentage of AGC losses among various data sources for different degradation drivers. High variability within driver categories underscores the need for locally informed aggregation of degradation impacts. Satellite-RS data, with increasing spatial and temporal resolution, can detect diverse forest disturbance drivers and fill gaps where repeat ground sampling is infeasible (Table 1). Our database and associated meta-analysis showed that a range of data sources can provide comparable estimates of carbon losses from different types of forest degradation for regional-extent applications, which can be used in carbon accounting. Therefore, our mean carbon loss estimates have the potential to directly contribute to the IPCC Emissions Factor Database (*102*) and to future updates to the IPCC GPG on NGHGIs or FRELs (Table 1, Fig. 8, and table S2) (*79*, *103*).

**Table 1.** Different data uses, best-available data, and outstanding gaps that are currently not in operational and policy-driven efforts.

Use of research outputs for various forest monitoring efforts	Spatiotemporal and precision requirements	Is research-driven data available?	Example products/uses	Necessary improvements
Conservation/restoration projects within Reducing Emissions from Deforestation, Degradation and enhancements of forest carbon stocks (REDD+) for other carbon credits	Small changes over small areas, high precision required given small total changes, estimates every 1–5 years	**Degradation AGC loss**: No freely available data. High-resolution private data may be available to support users	**Degradation loss estimates**: e.g., Ctrees REDD+AI platform or open access data integration (*86*, *109*)	**Degradation loss estimates**: Sensors that precisely measure biomass change in dense forests. Integration of ground and satellite sensing is likely to be required, with field protocols that appropriately sample degradation (*158*). Reducing costs and barriers for ALS/TLS is a priority.
		**Regeneration AGC gain:** High-resolution data are available for secondary forest but with high uncertainties at pixel scale. No such data available for recovering degraded forest.	**Regeneration AGC gain:** 1-km spatial scale global data on young secondary forest regrowth (*9*	**Regeneration AGC gain:** Improve representation of recovery following degradation, considering types and intensities. Reduce uncertainties based on repeat measurements at local extent, especially in undermeasured regions and at project level.
Country-level estimates (NGHGI/FREL), global stocktake, global carbon flux estimates	Small changes over large areas, estimates every ~5 years	**Degradation AGC loss:** Freely available data and algorithms are available	**Degradation AGC loss:** Previous studies (*23*, *30*) and studies used in this meta-analysis	**Degradation AGC loss:** Accurate distinction of degradation type. Move toward reducing uncertainty in current biomass estimates by enabling robust comparisons with other approaches (e.g., remeasurement), and calibration with field data from a wide variety of forest types and regions. Incorporate long-term degradation emissions into estimates.
		**Regeneration AGC gain:** Freely available data and algorithms are available	**Regeneration AGC gain:** Regionally aggregated and gridded data across the tropics for secondary and degraded forest recovery (*12*, *92*)	**Regeneration AGC gain:** Accurate distinction of regenerating forest types, with measure of uncertainty for spatial maps. Move toward reducing uncertainty by enabling robust comparisons with other approaches (e.g., remeasurement), and having improved spatial delineation of regenerating forest after different disturbance.
Near real-time alerting of disturbance and advanced planning of recovery potential in the path of intervention actions	Small changes over medium-to-large areas, subannual alerting, precise emissions estimate not needed; in regrowth, focus on areas with high regrowth potential as well as risks to regrowing forests	**Degradation AGC loss:** Freely available data and algorithms are available	**Degradation AGC loss:** e.g., Reiche *et al.* (*86*)+ existing estimates of emissions	**Degradation AGC loss:** Distinguishing deforestation from degradation, and disturbance types in near-real time
		**Regeneration AGC gain:** Emerging data sources of regrowth potential and associated carbon removal potential	**Regeneration AGC gain:** Spatially explicit information on where it is biophysically possible for regeneration (*159*) and carbon storage potential (*160*)	**Regeneration AGC gain:** Beyond considering where it is biophysically suitable for forest to regenerate, it is crucial to consider what is socially feasible
Scientific/social understanding of long-term trends in ecosystem health and function (biomass, understanding long-term cycles of loss and growth) to support the IPCC process (e.g., assessment reports)	Small changes over medium-to-large areas, few strict temporal requirements, precise emissions estimate not needed	Freely available satellite data do not provide enough information. Likely to be missing key differences in loss/succession patterns. ALS and field studies show promise.	**Degradation AGC loss:** e.g., estimates used and provided in this study	All points above are valid
			**Regeneration AGC gain:** e.g., estimates used and provided in this study	

In addition to attributing carbon losses to proximate disturbance drivers, it is also useful to categorize disturbances by their intensity and reoccurrence (single versus cumulative). For example, our synthesis suggests that AGC losses per unit area from extreme drought are low (Fig. 4), but these can drive substantial aggregated emissions due to their large spatial extent (*30*) and decadal legacy mortality effects (*104*) and can trigger other disturbances (*42*). While satellite-RS has historically relied on single, binary classifications of disturbed versus undisturbed forest (*105*), and underestimated small-scale or under-canopy impacts (*106*), it is increasingly moving toward quantifying disturbance frequency and the intensity (*84*, *107*, *108*). Satellite-RS data are therefore well positioned to support country reporting in a unique way. Emerging RS data and technologies are starting to offer attribution to degradation drivers such as logging and fire [e.g DIST-ALERT, RAdar for Detecting Deforestation (RADD alerts), and Land Use Change Alerts (LUCA)] (*109**–**112*) (Table 1), and some studies show that RS-derived disturbance metrics correlate with percent biomass loss (*26*, *87*). Future research is therefore merited to enable assessments of the intensity of (carbon) impacts. Treating disturbance as a continuum from low-impact degradation to full forest clearance (Fig. 6) would improve comparability across sites and forest types, strengthen field and satellite-RS data integration, and better represent the complex disturbance-recovery dynamics in tropical landscapes (*113*).

A key challenge is that forest degradation lacks a universal definition (*16*, *114*, *115*), because defining degradation is an inherently socio-political and technological construct, shaped by reporting frameworks, ecosystem context, and detection capability (*116*). Defining degradation is not a matter of semantics; labeling a forest as “degraded” has consequences for monitoring, reporting, and eligibility for results-based payments. Yet, some practices labeled “anthropogenic degradation” in the tropics are considered “forest management” in Europe (*117*). In the Brazilian Amazon, only illegal selective logging is currently reported to the United Nations Framework Convention on Climate Change as a form of degradation (*118*). Such restrictions to reporting frameworks will need to be readdressed as indirect anthropogenic degradation drivers (e.g., windthrow and extreme drought) cause greater carbon losses due to accelerated climate change (*119*). Technological advances may also reshape definitions, for example, as high-resolution imagery (<5 m) increasingly captures small-scale disturbances (*109*, *120*) (Table 1). Temporarily dense observations help distinguish land management types not discernible in annual data (e.g., forest clearing by fire versus delayed burning of debris for cultivation) (*121*) (Table 1). Long-term data distinguish “background” disturbances in stable forests from prolonged degradation that results in declining forest function (*122*, *123*). These temporal insights enable different communities to define and operationalize forest degradation in ways aligned with their research, policy, and reporting objectives.

### Regrowth and recovery

Our review has shown the value of regrowth rates derived from different data sources: No single data source alone will provide thorough regrowth assessments across all spatial extents. Field-site data have driven the science forward in determining different rates of forest regrowth after different prior land uses, with data integration showing optimal regeneration trajectories (*67*). Satellite-RS approaches now enable us to account for plot selection biases in field-site data (*124*) and to capture greater spatial and temporal variability in the regrowth rates of secondary regrowth forest, using a space-for-time substitution approach to overcome high uncertainty at the pixel scale (*95*). A multidata approach may be possible as the satellite-RS community moves toward high-quality, open-access, repeat biomass maps (*87*).

Our compiled database identifies that AGC accumulation estimates for secondary regrowth forest are readily available at project and regional extents (Table 1), though we caution that these should not be applied to different forest/biome types or further than the maximum age used to create the estimate (table S4). Large-scale AGC accumulation rates for recovering degraded forests are available when no locally relevant information is available (Fig. 6). Although there has been a recent push in the literature toward more geospatially explicit regrowth rates, for example, gridded at a 1-km spatial scale, using space-for-time substitution, machine learning, or a combination of both (Table 1), these approaches alone are insufficient. Locally relevant removal factors from consistent, repeat measurements of permanent plots (e.g., 2ndFor) remain essential.

In future research, it is worth exploring why we see consistent (though nonstatistically different) lower absolute regrowth rates in secondary regrowth forests derived from satellite-RS data, and higher rates in data integration studies (Fig. 7A). Uncertainties to explain this include the following: (i) uncertainties in both the satellite-derived and field-derived biomass estimates, which may in part be linked to different uses of allometric equations and wood density values and associated their uncertainties; (ii) mismatches in secondary forest age definitions due to potential underestimation of regrowth begin in satellite-RS data (point of detection in imagery) compared to field site (point of land abandonment); (iii) different assumptions on the remaining AGC before regrowth begins, especially small trees in early stages of regrowth (*125*); (iv) field plot selection bias (*95*, *124*), which may avoid or underrepresent areas affected by multiple disturbances; and (v) satellite-RS data capturing and reflecting a wider variety of rates of regrowth.

### Carbon fluxes across disturbance-recovery trajectories

Our review and associated meta-analysis highlight a crucial gap in our current framing of forest dynamics postdisturbance: With a few exceptions (*45*, *126*), studies rarely follow deforestation/degradation to regrowth/recovery over time, considering the ongoing spatiotemporal interactions between these two processes (Fig. 1). We find that most degradation studies do not conduct long-term monitoring after an initial loss event, with measurements occurring, on average, 2.5 (±2.3) years after disturbance. Regrowth studies often begin at deforestation or regrowth detection (*12*) and most measure secondary regrowth forests rather than recovering degraded forests.

As a result, the impact of legacy effects from degradation on recovery remain underexplored. Some disturbances, such as (recurring) fire and (long-term) drought (*15*, *23*), may cause forests to be a net carbon source for decades due to ongoing decomposition of dead biomass or persistent tree mortality rates (*15*, *23*, *127*). Studies indicate that Amazonian forests may be less drought resistant than African forests (*128*). Moreover, while most studies focus on AGC (*25*), this does not capture the entire ecosystem-level carbon dynamics. For example, logged forests may rapidly regain tree cover, yet elevated soil respiration and decomposition carbon fluxes can offset AGC gains, keeping the ecosystem a net carbon source for at least a decade (*129*). Our meta-analysis shows that the intensity of degradation influences subsequent recovery, with a clear connection between the trajectory of AGC gain and the amount initially lost (Fig. 6B and fig. S14). Deriving removal factors for different degradation types is currently limited (*15*, *92*) due to data gaps and limited available studies. Overall, despite the importance of accounting for temporal and legacy effects of degradation, there is no unified methodology or supporting data to do so. This leads to confusion for carbon reporting and accounting (*16*) and potentially missed emissions for the carbon budget (*36*).

We therefore highlight the value of long-term repeat field measurements that follow the pathway from degradation to recovery (*73*), exploring prior land use, degradation drivers, and disturbance intensity as predictors of forest regeneration. Increasingly longer forest disturbance/recovery analyses using RS time series may further enrich these analyses. Where possible, eddy-covariance flux towers can be used to capture ecosystem-scale carbon fluxes beyond AGC (*130*). When combined with carbon and disturbance information, these observations can provide a more complete picture of legacy degradation impacts and help reduce uncertainties in postdisturbance carbon balance. Further studies are needed to assess synergies and trade-offs with biodiversity goals during degradation and regeneration, including changes in species composition and diversity (*67*, *131*). Furthermore, ongoing and future climate change (*132*) will influence disturbance type and intensity (*133*), associated emissions, and potential regrowth (*73*), with little current research into this in degraded/regenerating forests.

### Multidata integration for technological advances

Across the various data sources in our review and meta-analysis, there is potential for technological advances, either by integrating multiple data sources or by developing new technologies. For example, new satellite missions like ESA’s BIOMASS and NASA-ISRO’s NISAR will provide long-wavelength P- and L-band radar data, respectively, which are expected to improve sensitivity to woody-biomass components compared to optical and short-wavelength radar sensors (e.g., C-band Sentinel-1), especially in young secondary regrowth forest. Further, spaceborne lidar missions (e.g., NASA’s GEDI and proposed missions such as EDGE or VEGGIE-H) provide direct measurements of forest vertical structure, with repeat measurements enabling more accurate quantification of tree height and AGC changes.

Field data and airborne-RS imagery remain essential for validating satellite-derived biomass and activity data in accordance with IPCC GPG (*70*). In particular, calibration datasets from degraded and regenerating forests, spanning different disturbance types, extents, ages, and biomass ranges, are needed, because many existing biomass calibration datasets are from undisturbed forests (*95*). Field-site experiments also remain crucial for testing mechanistic hypotheses about the drivers of disturbances, including the roles of CO_2_ fertilization (*114*) and heat stress (*134*). Increasingly, many tropical countries have a robust NFI in place, providing an unbiased and long-term basis for ground-based monitoring of forest dynamics (*135*).

Airborne-RS and expanded satellite-RS data at very high resolution (*86*, *109*) could complement these efforts (*85*) by capturing small-scale structural changes to further help attribute the degradation drivers that are challenging to differentiate from space (*109*). Uncertainties in identifying the start of regrowth (*136*) can be addressed by combining field and satellite-RS data in disturbance areas (*98*). Projects such as GEOTREES, which establish field plots using TLS in regrowing and disturbed forests, are vital for addressing field plot selection bias and improving satellite-RS calibration outside undisturbed forests (*95*). Such technology may also help to improve allometric equations and wood density values, which are the foundation of any AGC estimates (*137*).

Last, substantial geographic gaps remain: Most (63%) of the studies in our meta-analysis were from the Americas, specifically the Amazon (50%) (figs. S15 and S16). The small sample sizes for some disturbance/regrowth types, e.g., extreme drought, may bias the calculated means in our meta-analysis. Furthermore, despite the wealth of information on secondary forest AGC accumulation, variations remain regarding regrowth rates across the pan-tropical continents. IPCC tier 1 removal factors for young secondary forests in tropical rainforests suggest that rates are highest in Africa, followed by America and Asia, but all with overlapping confidence intervals (*79*). Other data compilation studies (*9*, *138*, *139*) and satellite-based analyses (*12*, *41*, *44*) do not fully align with this pattern. Addressing these uncertainties requires more targeted, collaborative research, investment in sustaining and expanding the existing field data, and complementing these with satellite-RS data in underrepresented regions, such as African, Asian, and Oceanian tropical forests. Particular attention should be given to specific regrowth processes with remnant trees, restoration practices, and cultural land-use practices, which play a critical role in shaping regrowth trajectories. To support countries in developing locally relevant estimates, research efforts and methods must become more transparent, accessible, and better aligned with monitoring and reporting requirements.

### Forest policy rooted in scientific advances

The alarming trend of ongoing TMF degradation needs to be reversed (*24*). To achieve this, forest policies must be science-based, including transparent and accessible methods and data for estimating carbon losses and gains from degradation and regeneration. Our meta-analysis of 146 studies assessing the magnitude and variability of AGC losses and gains in TMF provides new resources to advance climate-related forest protection for a variety of users. These users include national and subnational policy-makers designing forest and climate strategies, national forest monitoring teams, carbon market regulators, and standard bodies seeking to prioritize mitigation efforts. Recognizing forest degradation, deforestation, and regeneration as interconnected and highly variable processes is fundamental to ensuring that methods, policy measures, and voluntary commitments are effectively designed and implemented.

Our review highlights how RS data can complement field data in key ways: by addressing field data selection bias, filling data gaps in inaccessible regions, and quantifying fluxes that are otherwise difficult to capture at landscape level, such as edge effects. Remaining uncertainties and variabilities can be characterized by considering the type and, crucially, intensity of the disturbance to improve estimates of biomass loss and gain. Data gaps remain most pronounced in Africa and Asia, particularly in long-term field plot networks, disturbance attribution, and capturing land-use and forest management practices.

By putting multiple studies in context with each other, we can understand the importance of different degradation drivers relative to deforestation carbon losses (per unit area). Such information can help direct attention to halting degradation in forest protection and climate mitigation policies. A similar comparison accounting for the complexities in the trajectories of recovering degraded forest would provide insights into effective forest restoration strategies, as has been happening for secondary regrowth forest, for which numerous estimates are available. Although continuous improvements in methodologies will enhance data precision, delaying action under the pretext of data uncertainty is no longer justifiable. Existing evidence—much of which has been available since the 1980s—is sufficient to estimate large-scale forest carbon fluxes and support informed mitigation efforts. This knowledge equips us to better protect and restore forests to deliver lasting climate and biodiversity actions in resilient landscapes.

## METHODS: META-ANALYSIS

We compiled a database of 146 peer-reviewed research studies (published between 1988 and 10 January 2026) on carbon losses and gains following disturbance and regeneration. We follow a similar approach to de Andrade *et al.* (*23*); using Google Scholar and Web of Science, we searched “tropical” with either “carbon” or “biomass,” and “degradation/disturbance” or “recovery/regrowth,” including both anthropogenic and natural disturbances. We also included cited references within studies. The goal of this rapid review (*140*) with meta-analysis was to synthesize AGC losses and gains from degradation and regrowth, respectively, across a range of approaches, and characterize their variation. These approaches included data from field sites, airborne-RS, satellite, and data integration. We use data integration to refer to studies that integrate multiple field data sources across large geographical extents.

For carbon loss, we included 84 studies that readily reported relative AGC losses (% loss relative to undisturbed forest) or absolute AGC values (Mg C ha^−1^), from which relative values could be easily calculated (table S6). For carbon gain, we included 84 studies that readily provided AGC gain estimates. Biomass values were converted to carbon using a factor of 0.456 (*141*). In both cases, by “readily,” we mean a study that was easily findable, and where the data on AGC losses and gains in the main article, Supplementary Materials, or data were presented such that they could be extracted without much additional manipulation and were easy to interpret, e.g., by providing values directly in text, in equations, or in figures. The aim of this exercise was to mirror a potential approach by people working in operational applications. For a few spatially explicit studies, we followed up with the corresponding authors to obtain information for the relevant TMF regions.

We report information on AGC losses and gains relative to prior or nearby undisturbed states to enable comparisons across natural biomass gradients and across study scales. However, comparing to nearby undisturbed forests may introduce uncertainty as older and younger forests often occur in different landscapes and environmental conditions; undisturbed forests, for example, typically grow at higher elevations and steeper slopes (*142*), and are thus an imperfect comparison to disturbed forests. Absolute carbon densities are given in the Supplementary Materials (figs. S6 to S8).

For this review, we focus on the proximate drivers but do not attempt to parse whether they are primarily due to anthropogenic or natural causes. Our review includes degradation from forest fires, selective logging, drought, edge effects, and windthrow. We do not include degradation from tsunamis/hurricanes/cyclones, lightning events that strike individual trees, or pest and disease outbreaks because of their highly localized or ephemeral nature and general limited data coverage.

We categorized studies by data source (field site, satellite-RS, airborne-RS, and data integration) (table S6) to demonstrate where and how different data sources are complementary and to highlight gaps in the utility of different data sources across regions and different drivers of disturbance/regeneration. For studies using multiple data sources, we assigned the study to the data source that derived AGC loss/gain, rather than the data source used to estimate the activity data. Following previous studies (*101*, *143*), we calculated summary statistics and used Tukey’s HSD test (*144*) and Hedge’s *g* effect size (*145*) to assess statistically significant differences between data groupings and the associated strength of the differences. The HSD accounts for subgroup sample size as well as the number of tests being run and is particularly useful for small sample sizes.

The relative magnitudes of AGC losses and gains may be influenced by or are relative to the study area/scale. To test whether there was a significant difference in disturbance/regrowth intensity across study types and the respective scale relevant to each study, we used linear mixed-effect models, including the reference ID as a random effect (table S5). Following previous reviews (*25*), the spatial extents of the studies were interpreted based on whether the study fell into being: local (site/landscape level); regional (state or multisite); subnational (approximately biome level); national (country scale); continental (multiple countries within a continent); or pantropical (single values relevant for the pantropics). The inclusion of “Extent,” as well as study type, accounts for the fact that, across the data sources used (e.g., satellite and field data), relative AGC losses and gains were estimated at different scales. All statistical analyses were carried out in the programming Language R (*146*).

## Supplementary Materials

### This PDF file includes:

Figs. S1 to S16 Tables S1 to S8 References
