# Supplementary material for: A meta-analysis of carbon losses and gains from tropical moist forest degradation and regeneration
Source: Sci Adv. 2026 Jul 3;12(27):eadz1923. doi: 10.1126/sciadv.adz1923 (PMC13330863; doi:10.1126/sciadv.adz1923)
Supplement: Supplementary file 1 — Figs. S1 to S16 Tables S1 to S8 References [file sciadv.adz1923_sm.pdf]

Science Advances
AAAS

Supplementary Materials for

A meta-analysis of carbon losses and gains from tropical moist forest degradation and regeneration

Viola Heinrich *et al.*

Corresponding author: Viola Heinrich, viola.heinrich@gfz.de

Sci. Adv. **12**, eadz1923 (2026)
DOI: 10.1126/sciadv.adz1923

This PDF file includes:

Figs. S1 to S16
Tables S1 to S8
References

Supplementary Information

Supplementary Figures

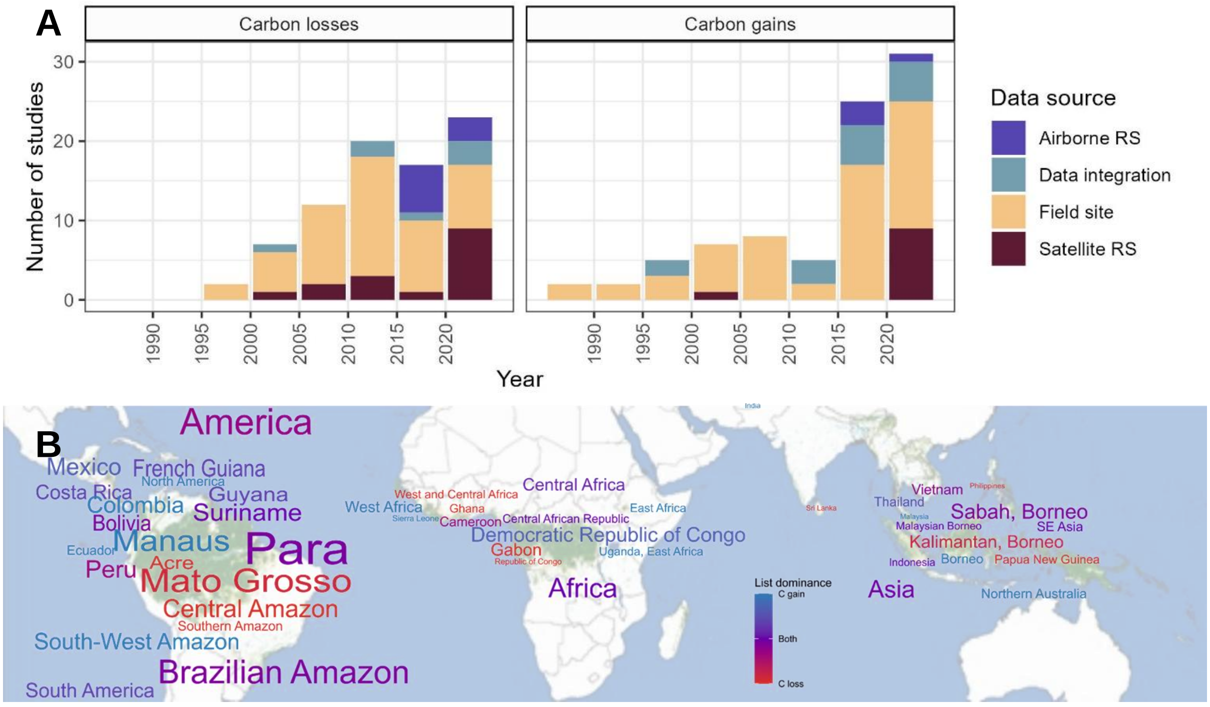

Supplementary Figure 1. Summary of the studies included in the meta-analysis. (a) Evolution of the studies, based on data sources through time. (b) WordCloud with the dominant subregion assigned to each of the studies. The basemap shows the approximate geographic location across the Tropical Moist Forest belt according to Vancutsem et al. Colours indicates if the subregion included studies mainly containing information on Carbon loss (C loss: red) or Carbon Gain (C gain: blue) or an equal amount of information from both disturbance and regrowth (Both: purple). *Note: the geolocations of the words were pseudo-randomly assigned for readability and may not align with their geographic region.*

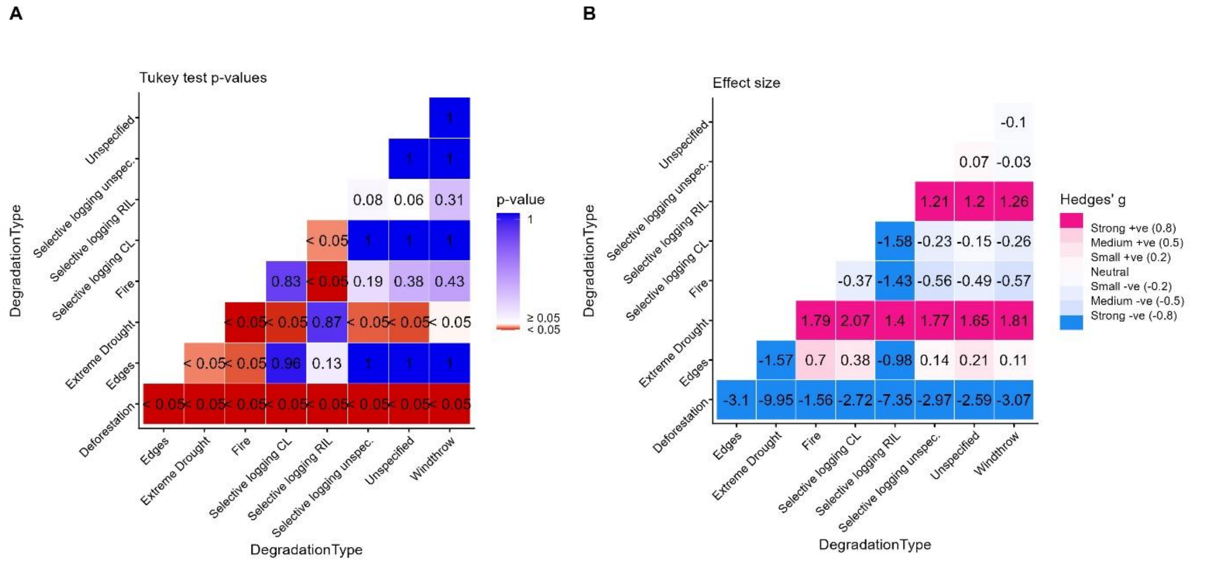

Supplementary Figure 2. Correlograms showing the outputs from various statistical tests between degradation types. (a) p-values from Tukey's HSD test to determine statistically significant differences between degradation types**.** Values show the p-values, where $p < 0.05$, this has been rounded to $p < 0.05$. Shading relates to the p-values, where hues of red are $p < 0.05$ and hues of blue are $p >= 0.05$. (b) Hedges's g effect sizes to determine the strength of the association.

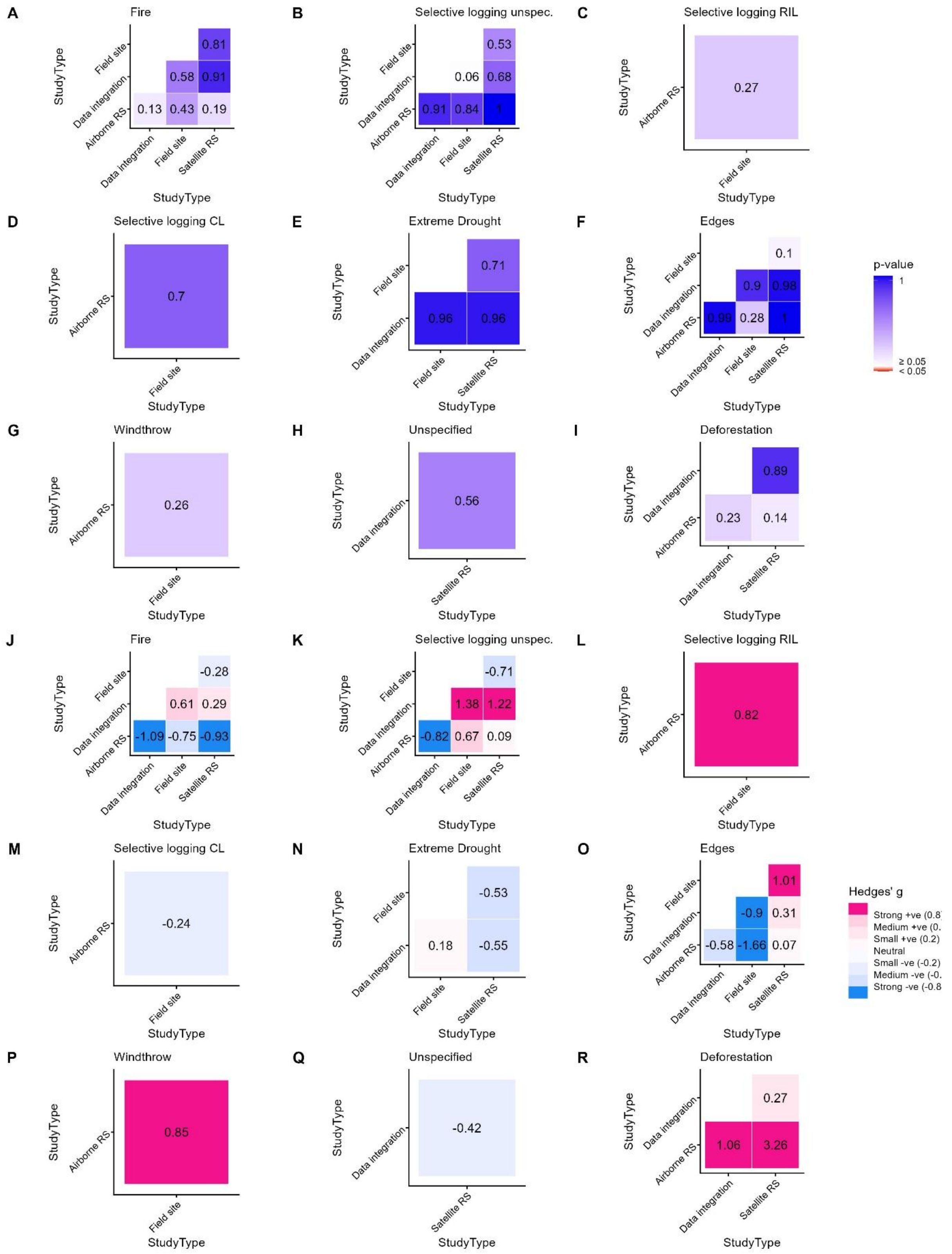

Supplementary Figure 3. Correlograms showing the outputs from various statistical tests between different data sources (StudyType) within degradation types. a-i are the p-values from Tukey's HSD test to determine statistically significant differences, where $p < 0.05$, this has been rounded to $p < 0.05$. Shading relates to the p-values, where hues of red are $p < 0.05$ and hues of blue are $p >= 0.05$. (j - r) are the Hedges's g effect sizes to determine the strength of the association.

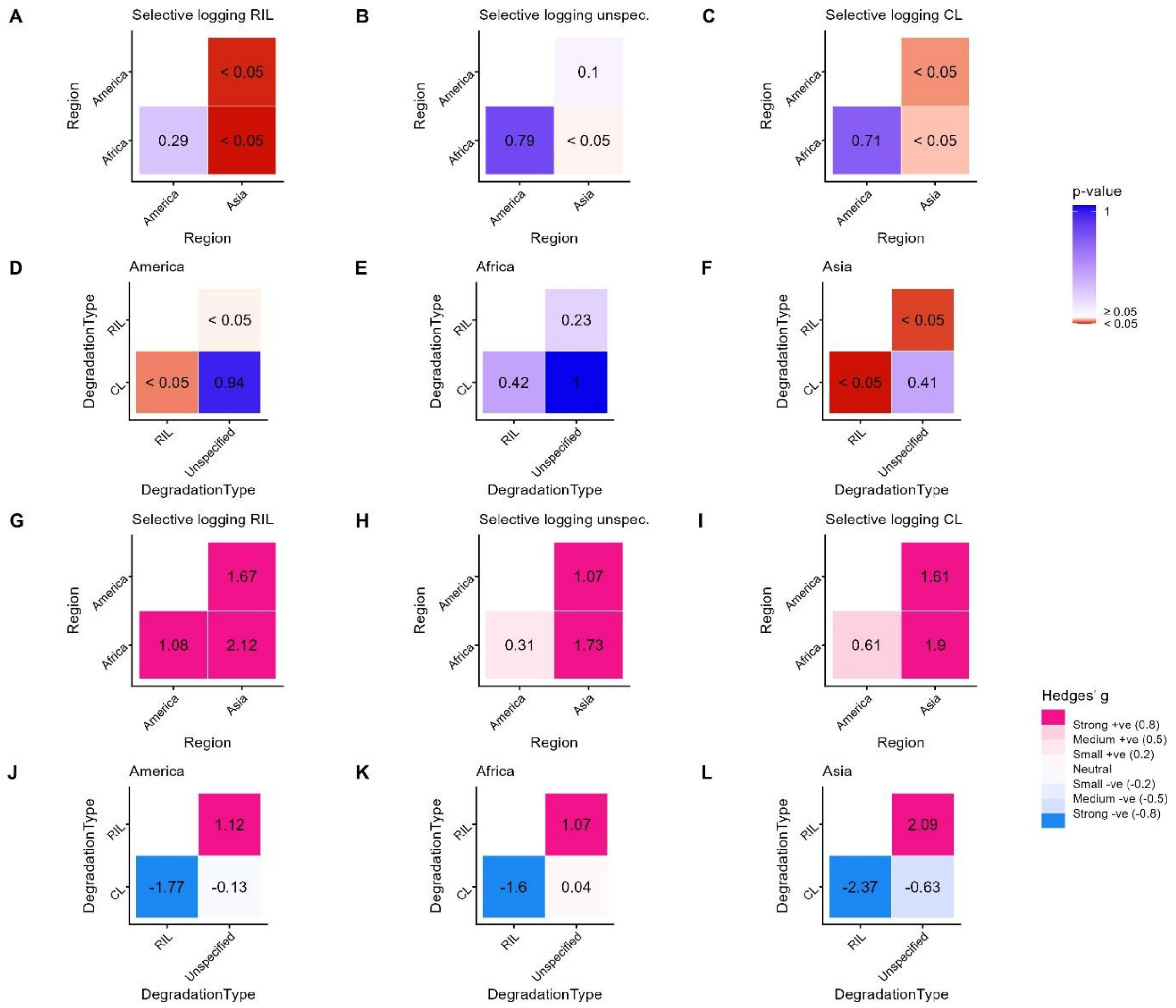

Supplementary Figure 4. Correlograms showing the outputs from various statistical tests between different intensities of selective logging activities. a-f are the p-values from Tukey's HSD test to determine statistically significant differences, where $p < 0.05$, this has been rounded to $p < 0.05$. Shading relates to the p-values, where hues of red are $p < 0.05$ and hues of blue are $p >= 0.05$. (g - l) are the Hedges's g effect sizes to determine the strength of the association. (a – c) and (g – i) relate to the different regions in the respective types of selective logging, while (d – f) and (j – l) relate to the logging intensities in the respective regions.

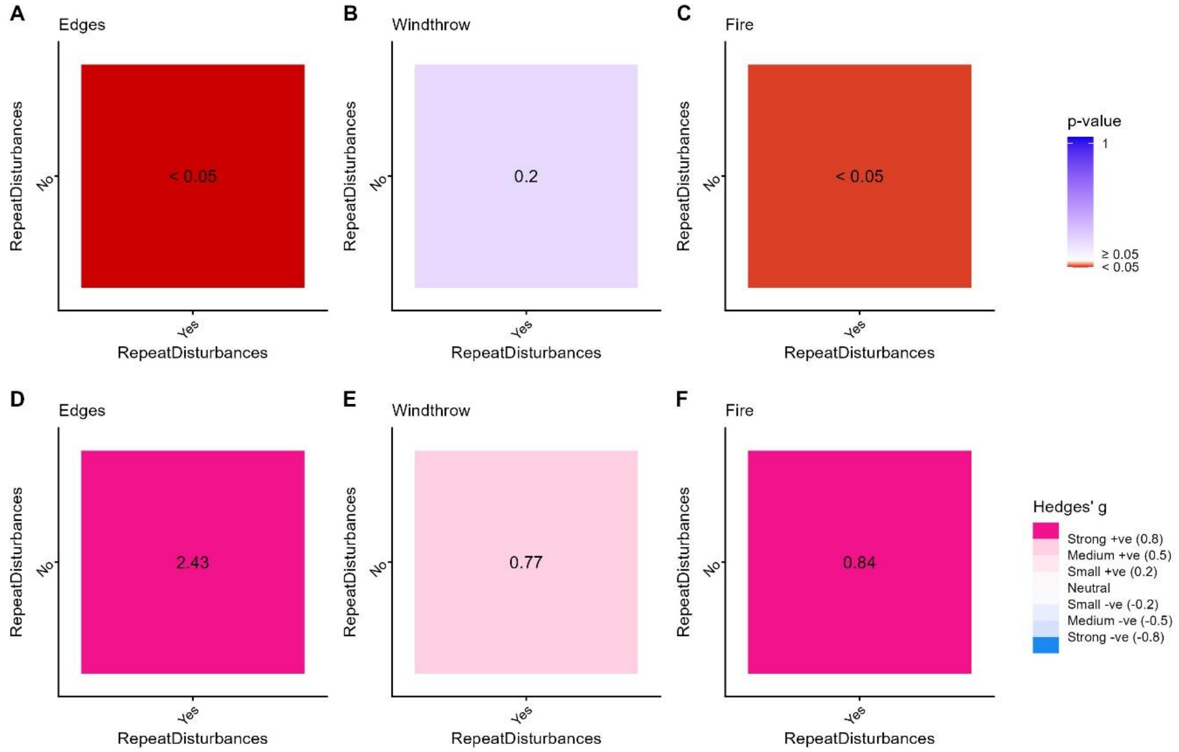

Supplementary Figure 5. Correlogram showing the outputs from various statistical tests between disturbance events that were repeated/co-occurring (Yes) or single events (No). (a), (b), (c) compare p-values for Edge effects, Windthrow and fire, respectively. P-values are from Tukey's HSD test to determine statistically significant differences between repeated disturbance events**.** Values show the p-values, where p < 0.05, this has been rounded to p < 0.05. Shading relates to the p-values, where hues of red are p < 0.05 and hues of blue are p >= 0.05. (d - f) Hedges's g effect sizes to determine the strength of the association.

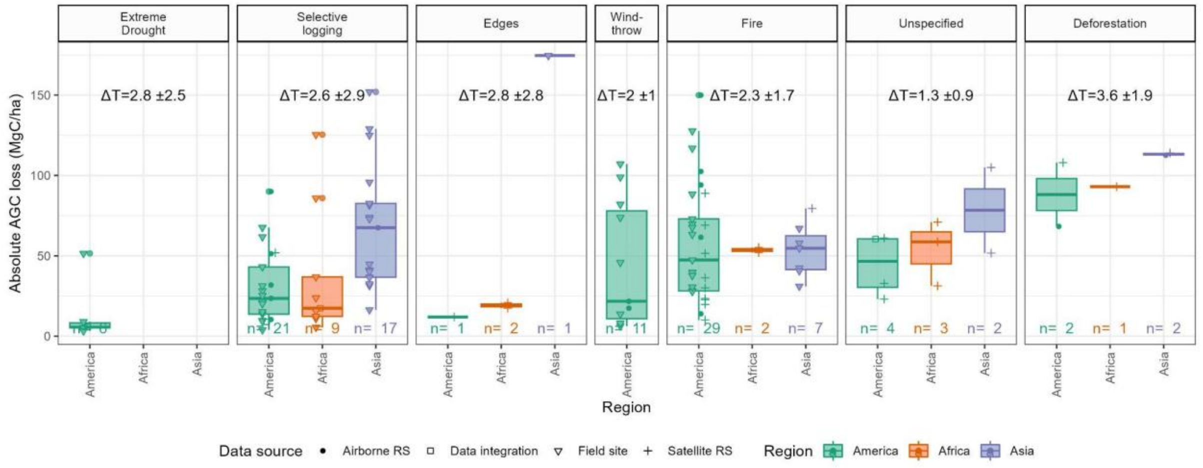

Supplementary Figure 6. The absolute AGC losses for different disturbance types compared to nearby or prior undisturbed forest state. The different disturbance types that can cause deforestation and degradation and the carbon losses as identified by different data sources. ΔT refers to the average number of years between the disturbance event and when the measurements for the study were taken, ± is the associated standard deviation. N is the number of datapoints. We acknowledge that there may be overlaps in the datasets used by each study, especially the data integration studies.

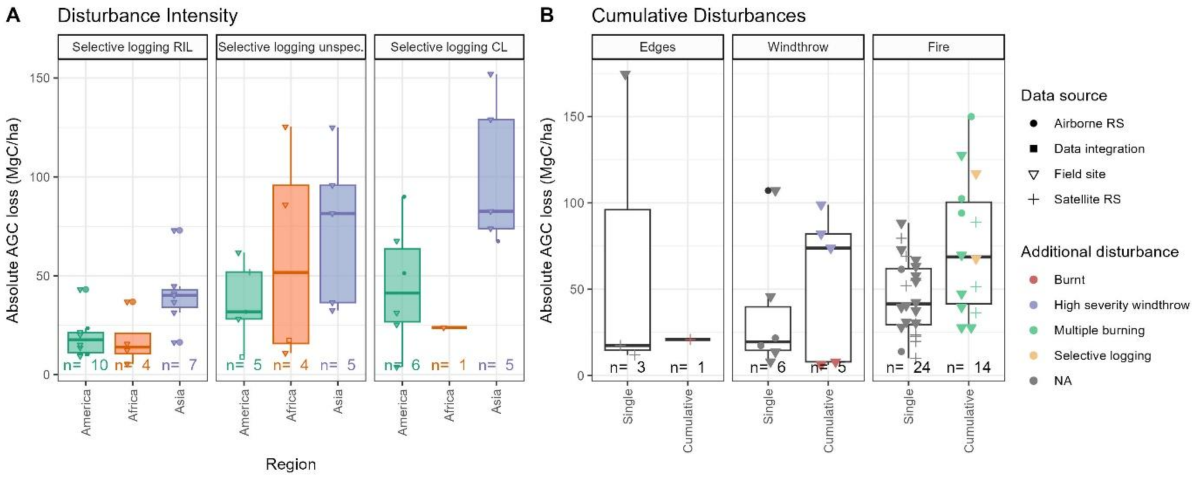

Supplementary Figure 7. The absolute AGC losses considering different intensities and cumulative effects of disturbances. (a) The carbon deficits across Reduced Impact Logging (RIL), Conventional selective logging (CL) and where the type of selective logging was unspecified split up according to the three major tropical regions (colours) and by data source type (shapes). (b) The impact of cumulative disturbances which includes repeat disturbances, where the study observed two or more disturbances over multiple years at the same location, and co-occurring disturbances which refers to different disturbance types occurring at the same time or space.

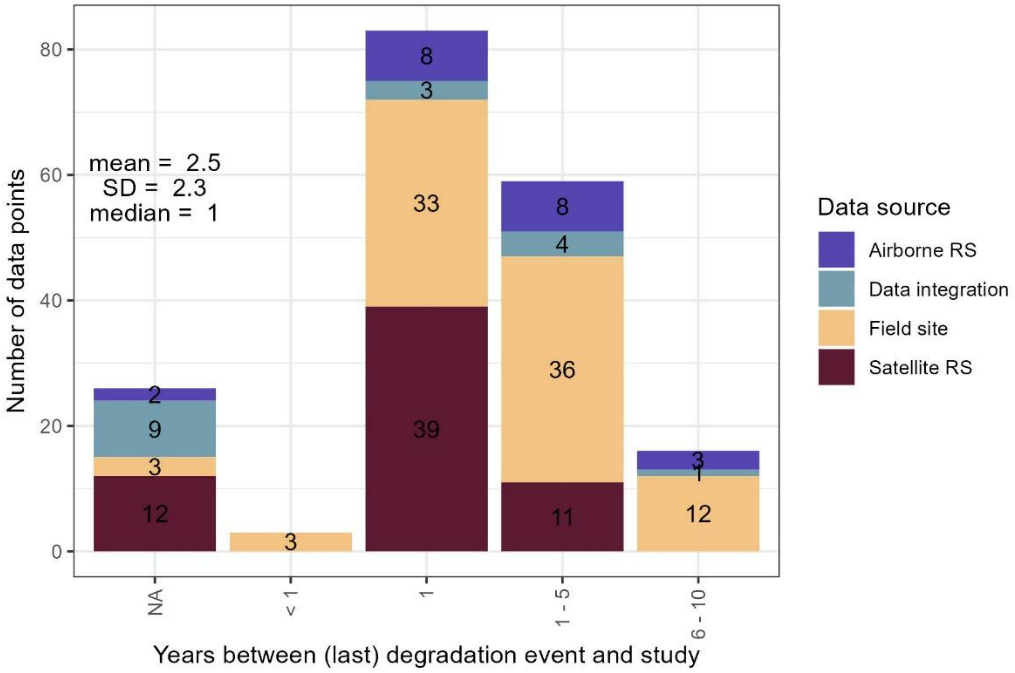

Supplementary Figure 8. Bar graph summary of the number of years between the disturbance event and when the measurement for the study was taken. Text shows the mean, associated standard deviation and median years. The mode is 1 as can be seen from the graph.

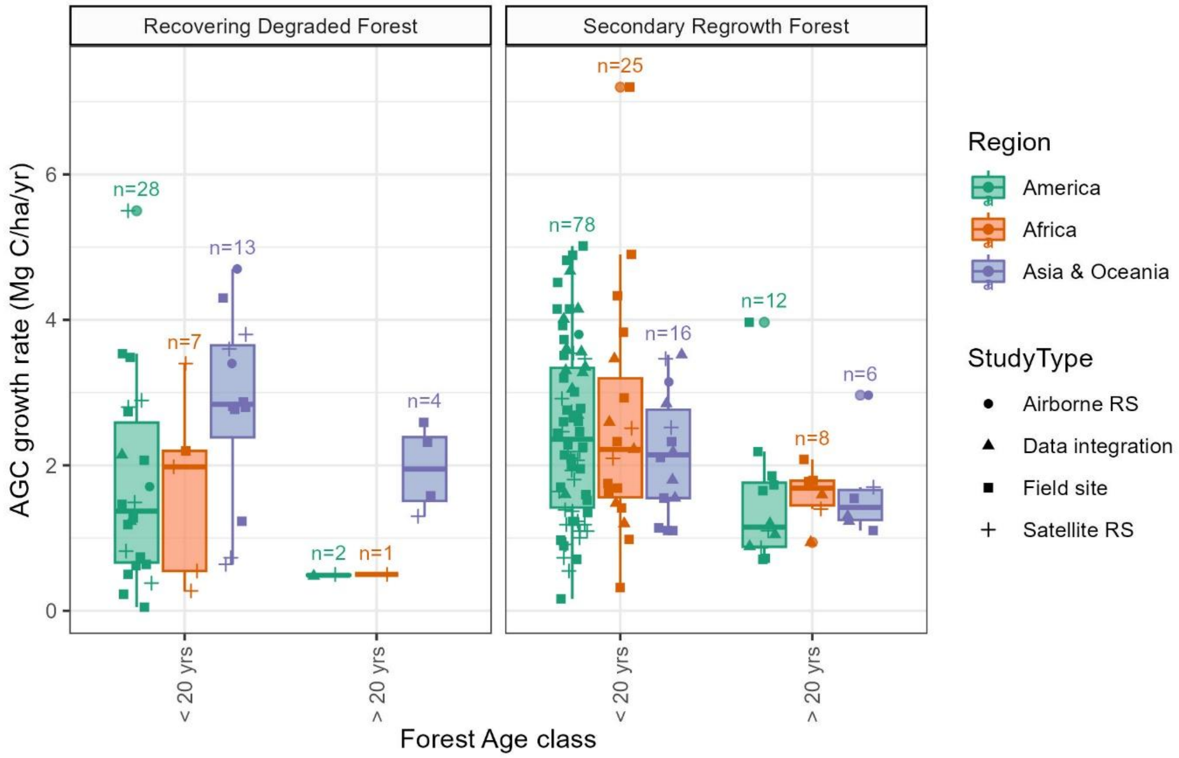

Supplementary Figure 9. Boxplots of the absolute aboveground carbon growth rate (in Mg C/ha/yr) in degraded and Secondary Regrowth Forest across the tropics. Points represent individual inputs from different studies according to the data source (shapes), with n= indicating the total number of inputs for each region and forest type. X axis has further grouped forest according to their age or years since last disturbance, names < 20 years and >20 years.

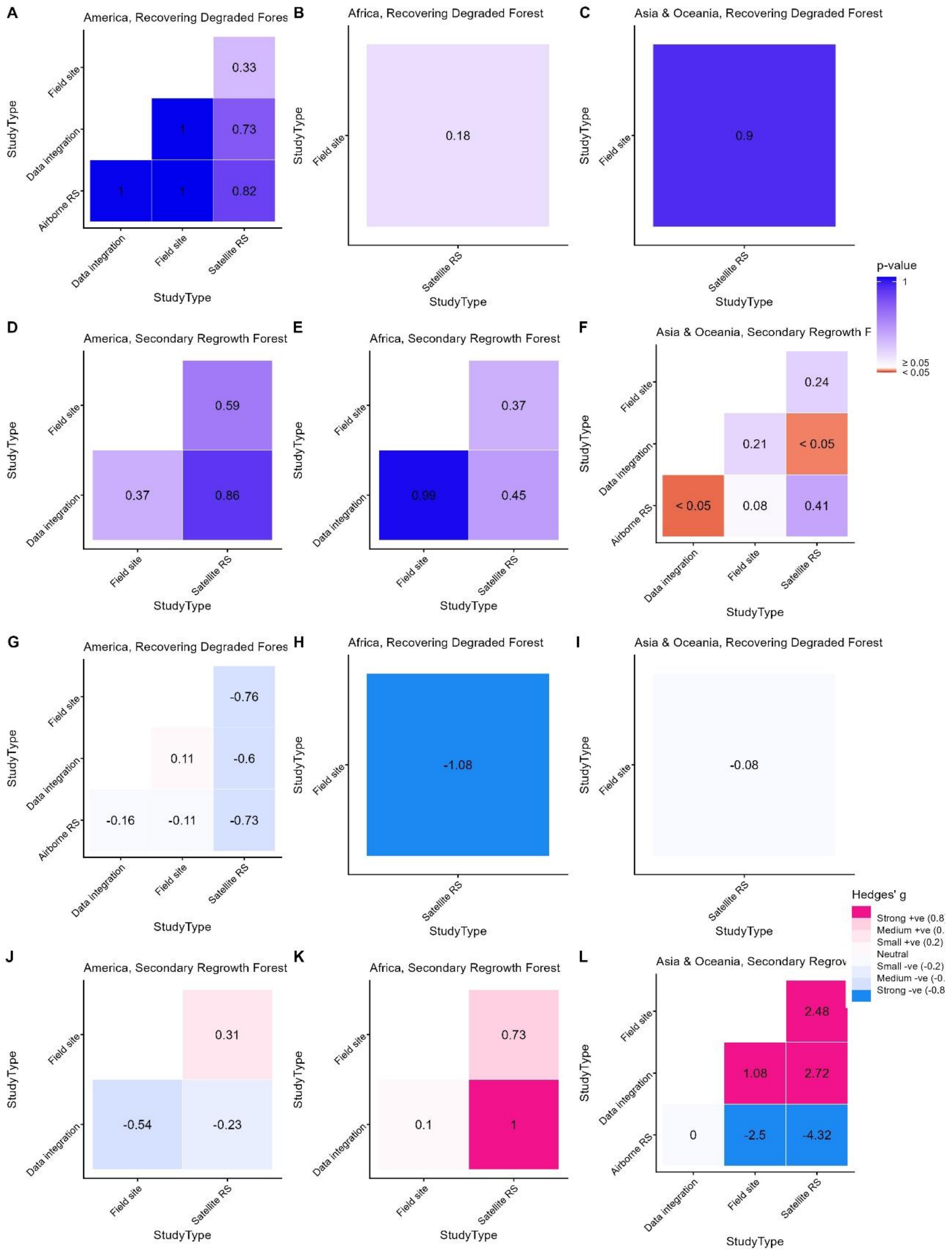

Supplementary Figure 10. Correlograms showing the outputs from various statistical tests between different data sources (StudyType) within different regenerating forests, according to the relative carbon gain after 20 years. (a – f) are the p-values from Tukey's HSD test to determine statistically significant differences, where $p < 0.05$, this has been rounded to $p < 0.05$. Shading relates to the p-values, where hues of red are $p < 0.05$ and hues of blue are $p >= 0.05$. (g – i) are the Hedges's g effect sizes to determine the strength of the association. Where a-c and g-i are for recovering degraded forest, and d-f and j-l are for secondary regrowing forest.

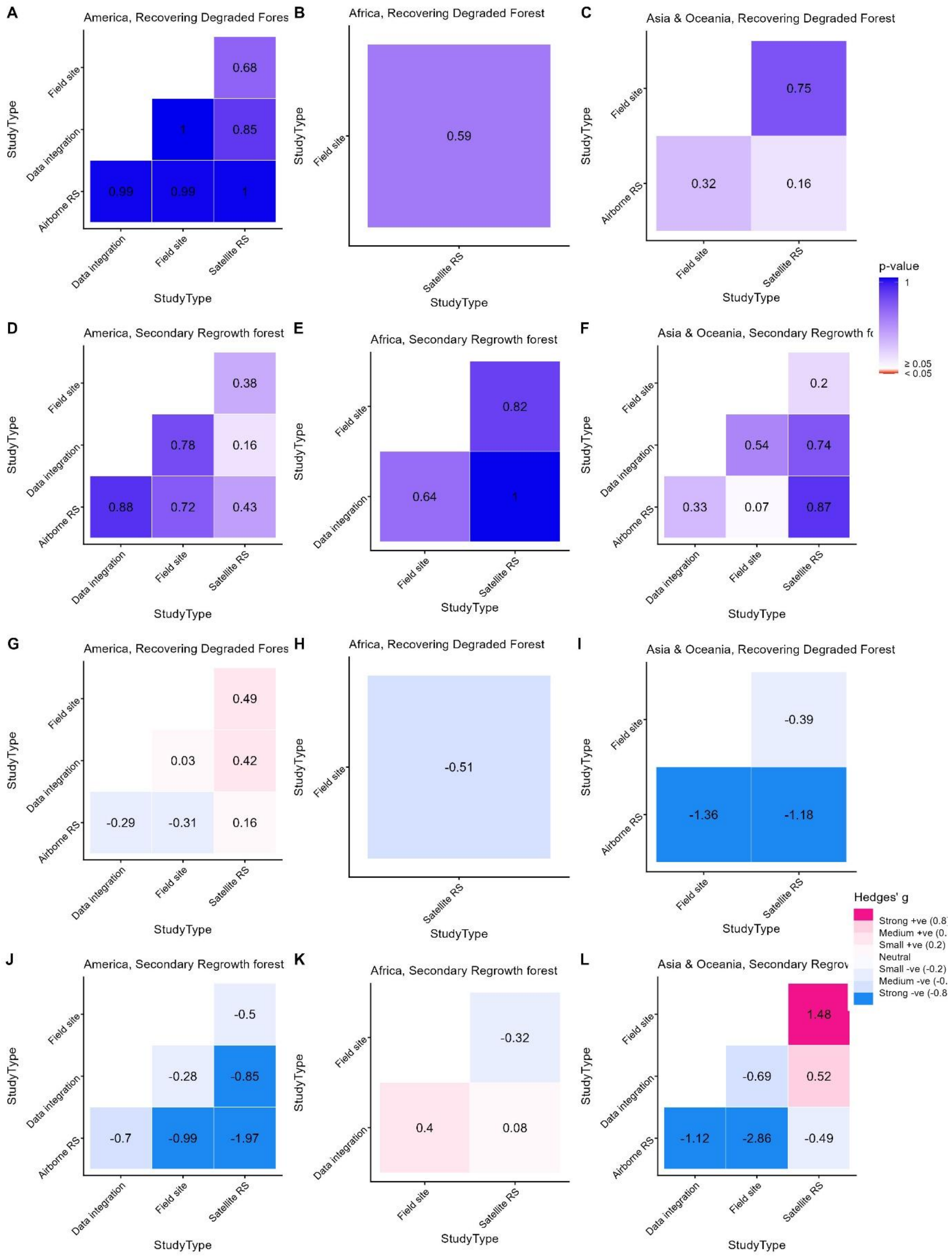

Supplementary Figure 11. Correlograms showing the outputs from various statistical tests between different data sources (StudyType) within different regenerating forests, according to the absolute AGC growth rates. As supplementary Figure 9, but for the Absolute Growth rates.

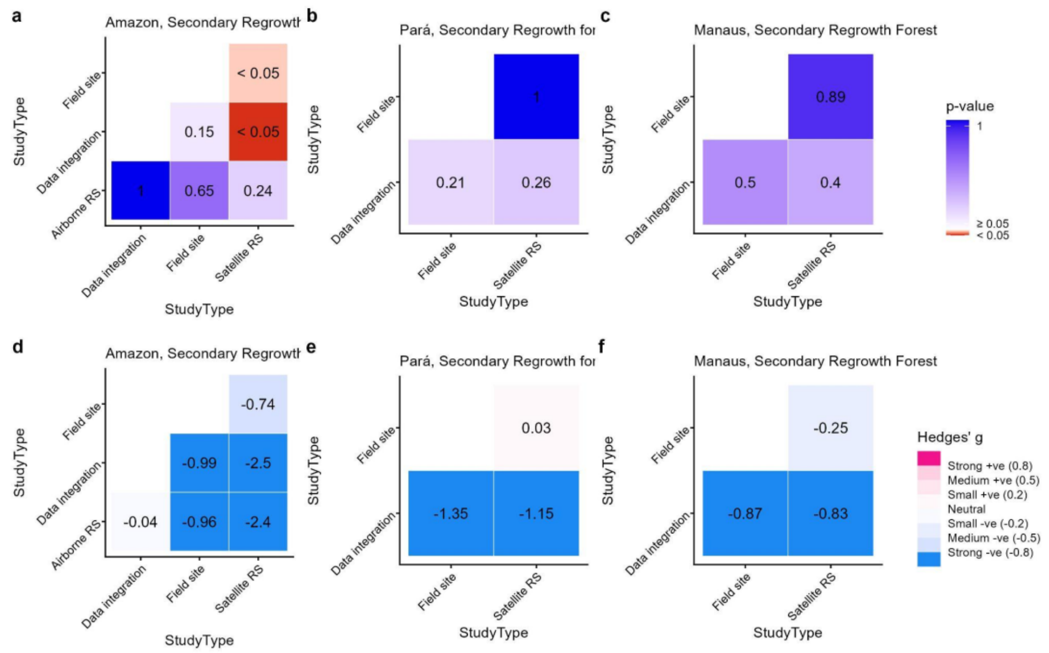

Supplementary Figure 12. Correlograms showing the outputs from various statistical tests between data sources (StudyType) within young Secondary Regrowth Forest, according to the absolute AGC growth rates in the Amazon. (a – c) are the p-values from Tukey's HSD test to determine statistically significant differences, where p < 0.05, this has been rounded to p < 0.05. Shading relates to the p-values, where hues of red are p < 0.05 and hues of blue are p >= 0.05. (d – f) are the Hedges's g effect sizes to determine the strength of the association. Where (a & d) is for the whole Amazon biome, (b & e) for Pará, and (c & f) for Manaus.

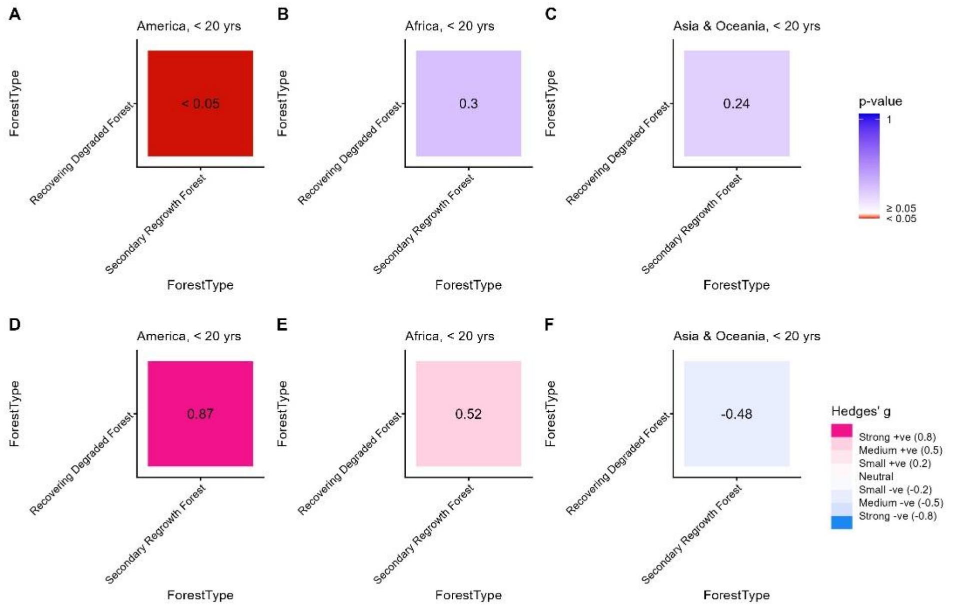

Supplementary Figure 13. Correlograms showing the outputs from various statistical tests between different forest types within young secondary regrowth forest, across the regions. (a – c) are the p-values from Tukey's HSD test to determine statistically significant differences, where p < 0.05, this has been rounded to p < 0.05. Shading relates to the p-values, where hues of red are p < 0.05 and hues of blue are p >= 0.05. (d – f) are the Hedges's g effect sizes to determine the strength of the association. Where (a & d) is for America, (b & e) for Africa, and (c & f) for Asia & Oceania.

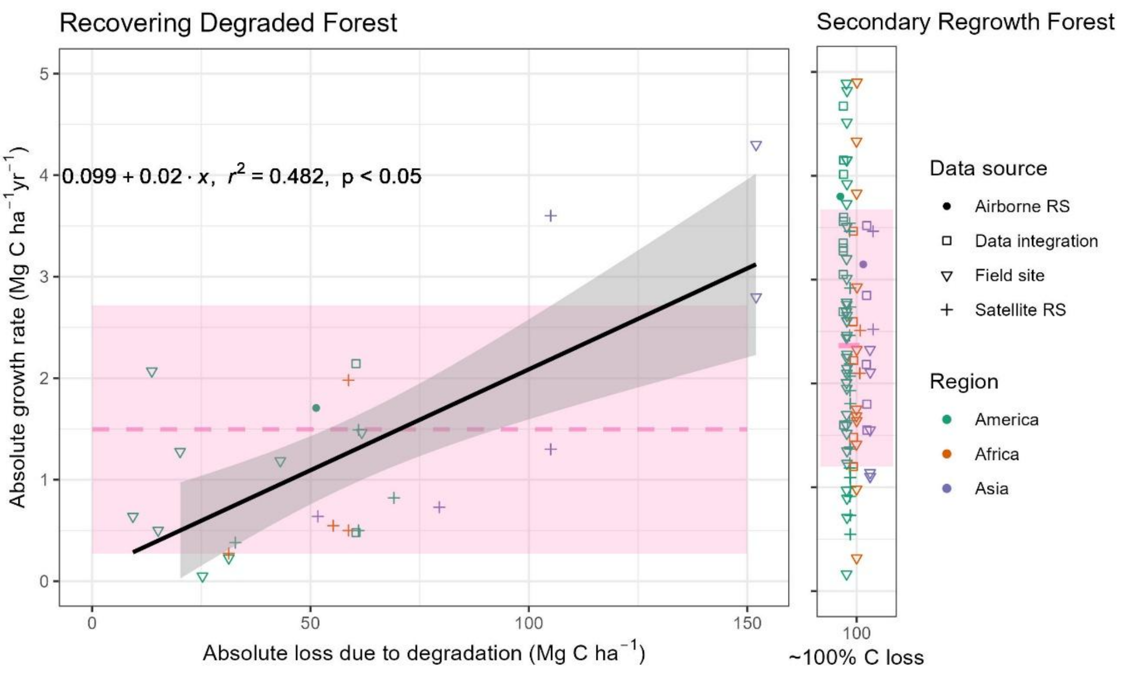

Supplementary Figure 14. The carbon losses due to degradation and carbon gains in recovering degraded forest and secondary regrowth forest. The absolute carbon losses and associated gains based on studies that captured both disturbance and regeneration in Degraded and Secondary Regrowth Forest. Horizontal lines are the mean and standard deviation of annual C increase.

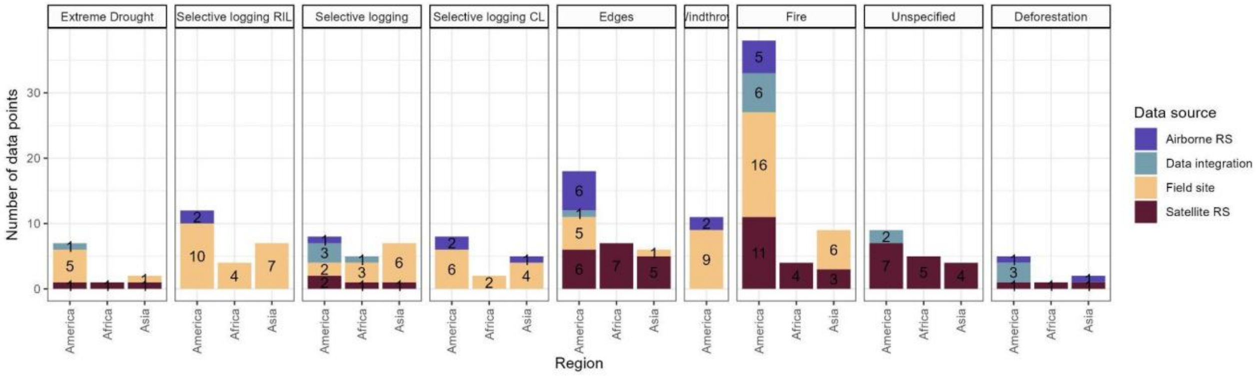

Supplementary Figure 15. The total number of data points collected for each disturbance type and data source across the regions.

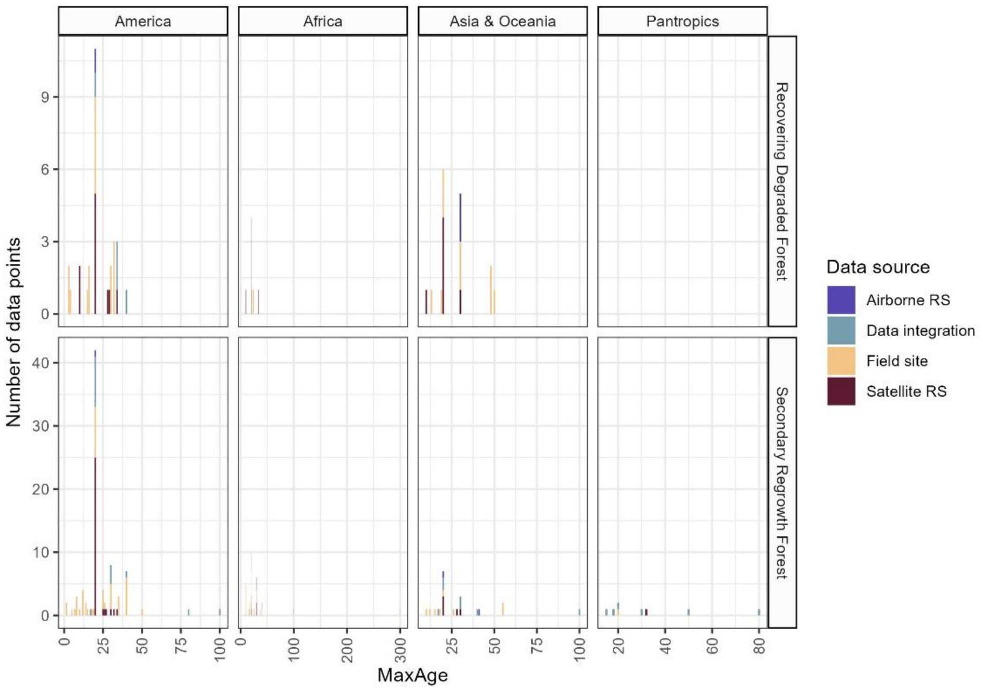

Supplementary Figure 16. The total number of data points collected for each regenerating forest type and data source. The panels have been split up across the regions by considering the Maximum Age (or years since last disturbance) as recorded in the study.

Supplementary Tables

Supplementary Table 1. Examples of policies, international agreements and pledges, linked to reducing tropical deforestation and degradation as well as enhancing forest regeneration.

Policy	Start Year	Explanation	Key data sources that will help to address policy.
Bonn Challenge[1]	2011	"Restore 150Mha of degraded and deforested landscapes by 2020 and 350Mha by 2030."	EO data to determine restoration success of degraded landscapes.
New York Declaration on Forests	2014	"End natural forest loss by 2030, with a 50% reduction by 2020."	
Paris Agreement	2015	Countries submit their NDCs. 1/4 of emission reductions expected to come from forests[2]	Field site data (National Forest Inventories, NFI) for countries to monitor forest changes through time as well as EO data to expand and extrapolate data across whole country to integrate into their National Greenhouse Gas Inventory (NGHGI).
UN decade on Ecosystem restoration	2021	"Halt degradation and restore ecosystems"	EO data to determine restoration success of degraded landscapes.
Glasgow Declaration	2021	"Halt and reverse forest loss and land degradation by 2030"	NFI and EO data to determine national success in achieving this target.
European Union Regulation on Deforestation Free products (EUDR)	2026	Specific products consumed by EU citizens do not contribute to deforestation/forest degradation worldwide after 31st December 2020.	EO data to determine extent of forest cover in 2020
Corporate Sustainability Due Diligence Directive (CSDDD)	2027	EU law that aims to ensure that large companies identify and address adverse human rights and environmental impacts within their operations and value chains. This includes integrating due diligence into corporate policies, monitoring effectiveness, and publicly communicating on due diligence efforts.	Land Use Cover and Change Maps, Carbon Maps, Biodiversity richness and many others
Corporate Sustainability Reporting Directive (CSRD)	2024	It aims to enhance and standardize sustainability reporting across companies operating within the EU.	Land Use Cover and Change Maps, Carbon Maps, Biodiversity richness and many others
2030 Agenda for	2015	UN SDG 15: "Protect, restore and promote sustainable use of terrestrial ecosystems,	Forest area as proportion of total land area, proportion of degraded land, progress towards sustainable

Sustainable Development		sustainably manage forests, combat desertification, and halt and reverse land degradation and halt biodiversity loss”	forest management, including afforestation and reforestation.
Kunming-Montreal Global Biodiversity Framework (GBF)	2024	Aims for “integrity, connectivity and resilience of all ecosystems are maintained, enhanced, or restored, substantially increasing the area of natural ecosystems by 2050”	Forest area, Forest distribution, Forest Fragmentation Index, Forest Landscape Integrity Index, Increase in secondary natural forest cover.
AFR100	2015	Country-led efforts to bring 100 million ha of land in Africa into restoration by 2030.	Project data outputs, NFI and EO data on degraded and recovering landscapes to determine regional success in achieving this target.
Initiative 20x20	2014	“A regional [Latin American] partnership to bring more than 50 million hectares of degraded land into the process of conservation and restoration by 2030”	Project data outputs, NFI and EO data on degraded and recovering landscapes to determine regional success in achieving this target.

Supplementary Table 2. Summary of the mean carbon losses in tropical moist forest for different Disturbance Types by region (as percentage loss compared to undisturbed forest or prior state of the forest before disturbance). Displayed *in the style of* the IPCC Tier 1 emission factors that *could* be in the IPCC GPG. Where Reference refers to the RefID as it appears in Supplementary Table 6.

Degradation Type	Region	Mean Loss (%)	SD	Average loss period (yrs)	Number of inputs	References (RefID)	Data Sources
**Deforestation**	Africa	87	NA	5	1	38	Satellite RS
	America	87	11.5	3.5	5	32, 38, 64	Airborne RS, Data integration, Satellite RS
	Asia	83.1	11.1	3	2	38, 70	Airborne RS, Satellite RS
	**Pantropical**	**86**	**9.8**	**3.6**	**8**	**32, 38, 64, 70**	**Airborne RS, Data integration, Satellite RS**
**Edges**	Africa	30.6	17.8	1	7	18, 26, 144, 145	Satellite RS
	America	27.5	17.1	3.86	17	18, 26, 57, 77, 97, 123, 145	Airborne RS, Field site, Satellite RS
	Asia	44.3	22.3	1	6	18, 26, 140, 145	Field site, Satellite RS
	**Pantropical**	**31.6**	**18.9**	**2.82**	**30**	18, 26, 57, 77, 97, 123, 140, 144, 145	**Airborne RS, Field site, Satellite RS**
**Extreme Drought**	Africa	0.8	NA	1.5	1	142	Satellite RS
	America	5.7	5.9	2.21	5	14, 36, 106, 122, 133, 142	Data integration, Field site, Satellite RS
	Asia	3.2	1.6	5.25	2	95, 142	Field site, Satellite RS
	**Pantropical**	**4.5**	**4.9**	**2.75**	**8**	**14, 36, 95, 106, 122, 133, 142**	**Data integration, Field site, Satellite RS**
**Fire**	Africa	48	19.9	2.75	4	18, 137, 141, 145	Satellite RS
	America	44.6	26.8	2.05	37	2, 4, 8, 9, 10, 11, 12, 18, 29, 30, 32, 52, 58, 81, 109, 112, 122, 141, 145	Airborne RS, Data integration, Field site, Satellite RS
	Asia	63.1	18.7	3.11	9	18, 126, 141, 145	Field site, Satellite RS
	**Pantropical**	**48.2**	**25.7**	**2.3**	**50**	**2, 4, 8, 9, 10, 11, 12, 18, 29, 30, 32, 52, 58, 81, 109, 112, 122, 126, 137, 141, 145**	**Airborne RS, Data integration, Field site, Satellite RS**
**Selective logging (ALL)**	Africa	17.4	13.5	0.8	11	5, 18, 40, 54, 73, 85, 86, 87	Data integration, Field site, Satellite RS
	America	23.4	15.7	1.88	27	5, 18, 32, 45, 47, 52, 64, 67, 81, 84, 89, 91, 96, 103, 107, 112, 116, 118, 120, 135, 138	Airborne RS, Data integration, Field site, Satellite RS
	Asia	42.1	17.5	4.5	19	7, 15, 18, 21, 35, 48, 55, 69, 76, 105, 108, 121, 127	Airborne RS, Field site, Satellite RS
	**Pantropical**	**28.5**	**18.6**	**2.62**	**57**	**5, 7, 15, 18, 21, 32, 35, 40, 45, 47, 48, 52, 54, 55, 64, 67, 69, 73, 76, 81, 84, 85, 86, 87, 89, 91, 96, 103, 105, 107, 108, 112, 116, 118, 120, 121, 127, 135, 138**	**Airborne RS, Data integration, Field site, Satellite RS**

**Selective logging (unspecified)**	Africa	23	16.2	1.33	5	18, 40, 73, 86	Data integration, Field site, Satellite RS
	America	30.6	21.3	1.83	7	18, 32, 52, 64, 67, 91, 103	Airborne RS, Data integration, Field site, Satellite RS
	Asia	48	11.1	3.07	7	15, 18, 35, 48, 76, 121, 127	Field site, Satellite RS
	**Pantropical**	**35**	**19.1**	**2.28**	**19**	**15, 18, 32, 35, 40, 48, 52, 64, 67, 73, 76, 86, 91, 103, 121, 127**	**Airborne RS, Data integration, Field site, Satellite RS**
**Selective logging CL**	Africa	22.3	12.4	0.58	2	54, 87	Field site
	America	31.5	13.9	2.21	8	52, 81, 112, 116, 118, 135, 138	Airborne RS, Field site
	Asia	57.1	16.1	4.62	5	7, 69, 105, 108, 127	Airborne RS, Field site
	**Pantropical**	**38.8**	**19.2**	**2.67**	**15**	**7, 52, 54, 69, 81, 87, 105, 108, 112, 116, 118, 127, 135, 138**	**Airborne RS, Field site**
**Selective logging RIL**	Africa	7.9	4.2	0.42	4	5, 85, 86, 87	Field site
	America	13.7	5.3	1.69	12	5, 45, 47, 81, 84, 89, 96, 107, 118, 120, 135, 138	Airborne RS, Field site
	Asia	25.6	8.9	5.86	7	21, 55, 69, 108, 127	Field site
	**Pantropical**	**16.3**	**9**	**2.84**	**23**	**5, 21, 45, 47, 55, 69, 81, 84, 85, 86, 87, 89, 96, 107, 108, 118, 120, 127, 135, 138**	**Airborne RS, Field site**
**Unspecified**	Africa	33	19.4	2	5	60, 137, 141, 145	Satellite RS
	America	34.8	16	1	9	22, 60, 62, 64, 114, 141, 145	Data integration, Satellite RS
	Asia	40.7	37.2	1	4	60, 141, 145	Satellite RS
	**Pantropical**	**35.6**	**21.5**	**1.27**	**18**	**22, 60, 62, 64, 114, 137, 141, 145**	**Data integration, Satellite RS**
Windthrow	America	33.6	19.6	2	11	32, 34, 82, 92, 124	Airborne RS, Field site

Supplementary Table 3. Summary of the mean and standard deviation carbon losses for cumulative disturbances in tropical moist forests. Carbon losses are expressed as the percentage (%) of carbon lost relative to nearby undisturbed forest or prior forest before disturbance.

Degradation Type	Repeat Disturbances	Mean C loss (%)	Standard Deviation (%)	Number of data points
**Edges**	No	24.1	24.4	26
**Edges**	Yes	56.3	18.1	7
**Windthrow**	No	26.4	26.4	6
**Windthrow**	Yes	42.2	17.5	5
**Fire**	No	41.4	24.4	39
**Fire**	Yes	61.9	26.2	16

Supplementary Table 4. Summary of the mean carbon gains for different regenerating forest types by region in tropical moist forest. Results are displayed by the absolute mean rate of carbon accumulation in young (<20 yrs) and older regenerating forest (> 20 yrs). Displayed *in the style of* the IPCC Tier 1 emission factors that *could* be in the IPCC GPG. Where Reference refers to the RefID as it appears in Supplementary Table 6.

Forest Type	Age	Region	Mean rate (Mg C/ha/yr)	SD of rate	Num of inputs (abs)	Mean C after 20 yrs of regrowth (%)	SD of C after 20 yrs (%)	Max. Age	Num. of inputs (%)	References (RefID)	Data Source
**Recovering Degraded Forest**	**< 20 yrs**	Africa	1.7	1.3	5	76.3	23.1	24	7	1, 18, 54, 60, 141, 90	Field site, Satellite RS
		America	1.7	1.3	22	80.4	23.6	34	24	1, 16, 18, 22, 37, 96, 47, 60, 84, 91, 112, 114, 118, 120, 135, 138, 141, 39, 146	Airborne RS, Data integration, Field site, Satellite RS
		Asia	2.8	1.5	10	58.4	13.5	30	8	1, 15, 18, 23, 60, 105, 141	Airborne RS, Field site, Satellite RS
		Oceania	2.8	0	2	63.5	9.2	20	2	66	Field site
		**Pantropical**	**2**	**1.4**	**39**	**74.6**	**22.6**	**34**	**41**	**1, 15, 16, 18, 22, 23, 37, 96, 47, 54, 60, 66, 84, 91, 105, 112, 114, 118, 120, 135, 138, 141, 90, 39, 146**	**Airborne RS, Data integration, Field site, Satellite RS**
	> 20 yrs	Africa	0.5	NA	1	NA	NA	34	0	60	Satellite RS
		America	0.5	0	2	NA	NA	40	0	60, 114	Data integration, Satellite RS
		Asia	1.8	0.7	2	NA	NA	50	0	60, 136	Field site, Satellite RS
		Oceania	2.1	0.7	2	NA	NA	48	0	66	Field site
		**Pantropical**	**1.3**	**0.9**	**7**	**NA**	**NA**	**50**	**0**	**60, 66, 114, 136**	**Data integration, Field site, Satellite RS**
**Secondary Regrowth Forest**	< 20 yrs	Africa	2.6	1.6	19	39.3	19.2	30	13	18, 31, 60, 96, 100, 101, 102, 111, 113, 139, 141, 88, 92, 13, 82, 33, 130, 115, 65	Data integration, Field site, Satellite RS
		America	2.5	1.2	70	37.1	16.3	40	62	3, 6, 18, 19, 25, 27, 28, 31, 32, 41, 43, 44, 46, 51, 53, 56, 59, 60, 61, 63, 68, 71, 72, 79, 80, 98, 101, 102, 110, 111, 113, 119, 128, 131, 141, 143, 117, 39, 42, 104, 115	Airborne RS, Data integration, Field site, Satellite RS
		Asia	2.2	0.8	14	36	8.6	55	11	18, 31, 49, 50, 60, 70, 78, 99, 101, 102, 113, 134, 141, 94, 115	Airborne RS, Data integration, Field site, Satellite RS
		**Pantropical**	**2.4**	**1.2**	**103**	**37.3**	**15.9**	**55**	**86**	**3, 6, 18, 19, 25, 27, 28, 31, 32, 41, 43, 44, 46, 49, 50, 51, 53, 56, 59, 60, 61, 63, 68, 70, 71, 72, 78, 79, 80, 96, 98, 99, 100, 101, 102, 110, 111, 113, 119, 128, 131, 134, 139, 141, 143, 88, 92, 13, 82, 117, 39, 33, 130, 42, 104, 94, 115, 65**	**Airborne RS, Data integration, Field site, Satellite RS**
	> 20 yrs	Africa	1.6	0.4	6	41.5	21.9	300	2	60, 96, 100, 113, 13, 115	Data integration, Field site, Satellite RS
		America	1.5	0.9	12	NA	NA	100	0	6, 25, 27, 28, 53, 60, 68, 113, 117, 115	Data integration, Field site, Satellite RS
		Asia	1.6	0.7	6	NA	NA	100	0	49, 60, 70, 113, 134, 115	Airborne RS, Data integration, Field site, Satellite RS
		**Pantropical**	**1.6**	**0.7**	**24**	**41.5**	**21.9**	**300**	**2**	6, 25, 27, 28, 49, 53, 60, 68, 70, 96, 100, 113, 134, 13, 117, 115	Airborne RS, Data integration, Field site, Satellite RS

Supplementary Table 5. Statistical summaries after running numerous mixed-effect models (in programme R) to further text the relationship between variables and the response variable (either AGC loss (Closs) or AGC gains (C20).

Model name	Model equation	Akaike Information Criterion (AIC) (ΔAIC)	p-value
		**% Carbon loss**	
Reference_deg	C_{loss} ~ 1 + (1 |RefID)	1706.6	NA
Extent_deg	C_{loss} ~ Extent + (1 |RefID)	1712.6 (+6.0)	**P < 0.1** for ExtentLocal<br>P > 0.1 for all other spatial extents
StudyType_deg	C_{loss} ~ StudyType + (1 |RefID)	1710.4 (+3.8)	P > 0.1 for all study types
Driver_deg	C_{loss} ~ DegradationType + (1 |RefID)	1638.6 (-68.0)	**P < 0.0001** for Degradation Types
		**% C gain after 20 years**	
Reference_reg	C_{20} ~ 1 + (1 |RefID)	1194.5	NA
Extent_reg	C_{20} ~ Extent + (1 |RefID)	1195.5 (+1.0)	P > 0.1 for all spatial extents
StudyType_reg	C_{20} ~ StudyType + (1 |RefID)	1199.3 (+4.9)	P > 0.1 for all study types
Forest_reg	C_{20} ~ ForestType + (1 |RefID)	1125.2 (-69.3)	**P < 0.0001** for Forest Types

Supplementary Table 6. Explanation of the variables extracted from the studies and presented in columns available in the databases on AGC losses and AGC gains, respectively.

Col. Letter	Column name	Description
	Degradation Dataset [AGC_losses_forest_type_current.xlx"]	
A	RefID	The numerical ID given to the study/reference. See 'reference_info.xsxl' for more information
B	Study	Lead author name and year of study
C	StudyType	Main data type used study (Field site, Airborne RS, Data integration or Satellite)
D	ForestType	The disturbance type i.e. either Degraded of Deforestation
E	DegradationType	The type of degradation explored by the study
F	Region	The large region to which the study pertains (America, Asia or Africa)
G	Sub-region	Information on the subregion in which the study took place, if applicable
H	Extent	The approximate spatial extent for which the study is relevant to: Local, Regional, Subnational, National, Continental, Pantropical
I	RepeatDisturbance	Whether the study explores the impact of repeat or co-occurring disturbances/degradation events (either Yes or No)
J	RepeatDisturbanceType	The type of repeat disturbance the study includes, if any.
K	Cpool	The carbon pools captured by the study
L	YSLD	The time period (in Years) between when a disturbance/degradation event took place, and when the study took place/measurements were taken
M	AbsoluteCLoss	The absolute carbon losses if given in the study, converted to MgC/ha
N	PercLossCompIntactFor	The percentage loss of carbon, relative to nearby undisturbed or prior state. (%)
O	errorMin	The minimum error value of the PercLossCompIntactFor, if any given.
P	errorMax	The maximum error value of the PercLossCompIntactFor, if any given.
Q	errorType	The type of error provided
R	Comment	Any additional comments to aid interpretation, or further information on where the information was sourced.
	**Regeneration Dataset [AGC_accumulation forest_type_current.xlx"]**	
A	RefID	The numerical ID given to the study/reference. See 'reference_info.xsxl' for more information
B	Study	Lead author name and year of study
C	Published_inPrep	Whether the study is published or in preparation/accepted.
D	StudyType	Main data type used study (Field site, Airborne RS, Data integration or Satellite)
E	ForestType	The forest type, either Recovering Degraded Forest of Secondary Regrowth Forest
F	DegradationType	The type of disturbance experienced by the forest
G	MaxAge	The maximum age included in the study
H	StandardisedAge	Based on MaxAge, the standardized age grouping used in this study, either < 20 yrs or > 20 yrs
I	Region	The large region to which the study pertains (America, Asia or Africa)
J	SubRegion	Information on the subregion in which the study took place, if applicable
K	Extent	The approximate spatial extent for which the study is relevant to: Local, Regional, Subnational, National, Continental, Pantropical
L	RepeatDisturbance	Whether the study explores the impact of repeat or co-occurring disturbances/degradation events (either Yes or No)
M	Yintercept	The value of C remaining after disturbance (in MgC/ha), if given
N	GrowthRate	The growth rate of AGC accumulation (in MgC/ha/yr) over the standardized age
O	errorMin	The minimum error value of the GrowthRate, if any given.
P	errorMax	The maximum error value of the GrowthRate, if any given.
Q	errorType	The type of error provided
R	Comment	Any additional comments to aid interpretation, or further information on where the information was sourced.
S	Yintercept_asPerc	The value of C remaining after disturbance (in%), if given
T	PercentageAt20yrs	The AGC after 20 years of regeneration, relative to nearby undisturbed or prior state, as a percentage
U	errorMinPercentage	The minimum error value of the PercentageAt20yrs, if any given.
V	errorMaxPercentage	The maximum error value of the PercentageAt20yrs, if any given.
W	errorType	The type of error provided of PercentageAt20yrs
X	Comment_relativeRate	Additional comments on the relativeRate, i.e PercentageAt20yrs

Supplementary Table 7. Table of all the references used in the synthesis. Where RefID refers to the number as it appears in the source data and supplementary Tables, and Reference citation, how it appears in the Reference list of the main paper.

RefID	Reference	Citation in Reference list
1	Achard et al. 2004	(*162*)
2	Alencar et al. 2006	(*163*)
3	Alves et al. 1997	(*164*)
4	Anderson et al. 2015	(*165*)
5	Aquino et al. 2022	(*26*)
6	Aryal et al. 2024	(*166*)
7	Asner et al. 2018	(*167*)
8	Balch et al. 2011	(*168*)
9	Balch et al. 2015	(*149*)
10	Barlow and Peres, 2004	(*170*)
11	Barlow et al. 2003	(*170*)
12	Barlow et al. 2012	(*171*)
13	Bauters et al. 2019	(*172*)
14	Berenguer et al. 2021	(*173*)
15	Berry et al. 2010	(*174*)
16	Blanc et al. 2009	(*175*)
17	Bonner et al. 2013	(*176*)
18	Bourgoin et al. 2024	(*41*)
19	Brazilian NGHGi, 2020	(*177*)
20	Brown and Lugo, 1990	(*178*)
21	Bryan et al. 2010	(*179*)
22	Bullock and Woodcock, 2021	(*126*)
23	Butarbutar et al. 2019	(*180*)
24	Carreiras et al. 2017	(*181*)
25	Cassol et al. 2019	(*182*)
26	Chaplin-Kramer et al. 2015	(*183*)
27	Chave et al. 2020	(*50*)
28	Chen et al. 2024	(*93*)
29	Cochrane et al. 1999a	(*184*)
30	Cochrane et al. 1999b	(*184*)
31	Cook-Patton et al. 2020	(*9*)
32	Csillik et al. 2024	(*80*)
33	Cuni Sanchez and Lindsell, 2016	(*185*)
34	Cushman et al. 2021	(*186*)
35	Cutler et al. 2020	(*187*)
36	da Costa et al. 2010	(*188*)
37	de Avila et al. 2018	(*189*)
38	De Sy et al. 2019	(*190*)
39	Doyle et al. 2025	(*191*)
40	Dupuis et al. 2025	(*192*)

41	Elias et al. 2022	(*73*)
42	Escobar et al. 2025	(*193*)
43	Faber-Langendoen, 1992	(*194*)
44	Feldpausch et al. 2004	(*195*)
45	Feldpausch et al. 2005	(*196*)
46	Feldpausch et al. 2007	(*197*)
47	Figueira et al. 2009	(*198*)
48	Fox et al. 2010	(*199*)
49	Fujiki et al. 2017	(*200*)
50	Fukushima et al. 2008	(*201*)
51	Gehring et al. 2005	(*202*)
52	Gerwing 2002	(*203*)
53	Giles et al. 2024	(*67*)
54	Gourlet-Fleury et al. 2013	(*204*)
55	Griscom et al. 2014	(*205*)
56	Guimaraes, 1993	(*206*)
57	Haneda et al. 2025	(*207*)
58	Haugaasen et al. 2003	(*208*)
59	Heinrich et al. 2021	(*11*)
60	Heinrich et al. 2023	(*12*)
61	Holcomb et al. 2023	(*88*)
62	Holcomb et al. 2024	(87)
63	Houghton et al. 2000	(*209*)
64	Houghton 2005	(*210*)
65	Houphouët et al. 2025	(*211*)
66	Hu et al. 2020	(*54*)
67	Huang and Asner 2010	(*212*)
68	Hughes et al. 1999	(*213*)
69	Imai et al. 2012	(*148*)
70	Jha et al. 2020	(*214*)
71	Johnson et al. 2001	(*215*)
72	Kalamandeen et al. 2020	(*216*)
73	Kenne Tene et al. 2023	(*217*)
74	Kenzo et al. 2015	(*218*)
75	Lapola et al. 2023	(*30*)
76	Lasco et al. 2006	(*219*)
77	Laurance et al. 1997	(*40*)
78	Lawrence, 2005	(*220*)
79	Lennox et al. 2018	(*19*)
80	Letcher and Chazdon 2009	(*221*)
81	Longo et al. 2016	(*222*)
82	Makelele et al. 2021	(*223*)
83	Marra et al. 2018	(*152*)
84	Mazzei et al. 2010	(*224*)
85	Medjibe et al. 2011	(*225*)
86	Medjibe et al. 2012	(*226*)

87	Medjibe et al. 2013	(*227*)
88	Michel et al. 2022	(*228*)
89	Miller et al. 2011	(*229*)
90	Mokake et al. 2024	(*230*)
91	Montero and Ellis, 2023	(*231*)
92	Moonen et al. 2019	(*232*)
93	Munoz et al. 2021	(*233*)
94	Murali et al. 2025	(*234*)
95	Murphy et al. 2013	(*235*)
95	Neves d'Oliveira et al. 2024	(*236*)
96	N'Guessan et al. 2019	(*51*)
97	Numata et al. 2011	(*237*)
98	Oberleitner et al. 2021	(*238*)
99	Ohtsuka, 2001	(*239*)
100	Ojoatre et al. 2024	(*240*)
101	Pan et al. 2011	(*139*)
102	Pan et al. 2024	(*1*)
103	Pearson et al. 2014	(*241*)
104	Pérez Abadía et al. 2025	(*242*)
105	Philipson et al. 2020	(*53*)
106	Phillips et al. 2009	(*243*)
107	Pinage et al. 2019	(*244*)
108	Pinard and Putz 1996	(*245*)
109	Pontes-Lopes et al. 2021	(*246*)
110	Poorter et al. 2016	(*10*)
111	Poorter et al. 2021	(*48*)
112	Rappaport et al. 2018	(*247*)
113	Requena Suarez et al. 2019	(79)
114	Requena Suarez et al. 2023	(98)
115	Robinson et al. 2025	(*138*)
116	Rockwell et al. 2014	(*248*)
117	Rodríguez-León et al. 2025	(*249*)
118	Roopsind et al. 2017	(*250*)
119	Rozendaal et al. 2017	(*251*)
120	Rutishauser et al. 2015	(*252*)
121	Saner et al. 2012	(*253*)
122	Silva et al. 2018	(*45*)
123	Silva Junior et al. 2020	(*15*)
124	Silver et al. 2000	(*254*)
125	Silverio et al. 2019	(*255*)
126	Slik et al. 2008	(*256*)
127	Stas et al. 2020	(*257*)
128	Steininger, 2000	(*258*)
129	Su et al. 2024	(*58*)
130	Titenwi et al. 2025	(*259*)
131	Uhl et al. 1988	(*260*)

132	Umunay et al. 2019	(*261*)
133	Uriarte et al. 2016	(*262*)
134	van Do et al. 2010	(*263*)
135	Vidal et al. 2016	(*264*)
136	Vijayanathan et al. 2025	(*265*)
137	Wanyama et al. 2025	(*266*)
138	West et al. 2014	(*267*)
139	Wheeler et al. 2016	(*268*)
140	Woodbury et al. 2024	(*269*)
141	Xu et al. 2026	(*92*)
142	Yang et al. 2022	(*44*)
143	Zarin et al. 2005	(*270*)
144	Zhao et al. 2021	(*271*)
145	Zhu et al. 2023	(*94*)
146	Zuleta et al. 2017	(*272*)

Supplementary Table 8. List of attendees of the R2D2 conference, held in March 2024.

First Name	Last Name	Institute
Alba	Viana Soto	TUM
Alexandra	Runge	GFZ
Amelia	Holcomb	University of Cambridge
Anne-Juul	Welsink	WUR
Arnan	Araza	WUR
Bart	Slagter	WUR
Ben	Newport	University of Bristol
Callum	Smith	University of Leeds
Camilo	Zamora	GFZ
Charlotte	Wheeler	University of Cambridge
Daniela	Requena	GFZ
David	Gibbs	WRI
Dominik	Spracklin	University of Leeds
Flavia	Mendes	Planet Lab
Gert-Jan	Nabuurs	WUR
Iain	McNicol	University of Edinburgh
Jens	Vanderzee	WUR
Jo	House	University of Bristol
Johannes	Reiche	WUR
Johannes	Balling	WUR
Katja	Berger	GFZ
Lina	Mercado	University of Exeter
Linda	Luck	GFZ
Luisa	Teixeira	Planet Lab
Martin	Herold	GFZ

Mengyu	Liang	UMD
Niamh	Kelly	WUR
Philippe	Ciais	LSCE
Robert	Masolele	WUR
Robert	Kennedy	Oregon State University
Robin	Chazdon	University of the Sunshine Coast
Rosie	Fisher	CICERO
Ruben	Valbuena	SLU
Sarah	Bereswill	PIK
Sietse	van der Woude	WUR
Simon	Besnard	GFZ
Stephen	Sitch	University of Exeter
Timothée	Stassin	GFZ
Toby	Jackson	University of Bristol
Tom	Pugh	Lund University
Una	Kelly	University of Münster
Viola	Heinrich	GFZ
Yidi	Xu	LSCE
Zhilin	Tian	GFZ
Aline	Pontes-Lopes	INPE
Amani	Bienvenu Konan	Université Félix Houphouët-Boigny
Andy	Marshall	University of Sunshine Coast
Annemarie	Eckes-Shephard	University of Lund
Anny	N'Guessan	Université Félix Houphouët-Boigny
Bruno	Herault	CIRAD
Camila	Silva	IPAM
Celso	Siva Junior	IPAM
Clement	Bourgoin	JRC
David	Lapola	University of Campinas
Giacomo	Grassi	JRC
Guido	Ceccherini	JRC
Henrique	Cassol	Bluebell Index
Jess	Baker	University of Leeds
Julie	Betbeder	CIRAD
Justin	Kassi	Université Félix Houphouët-Boigny
Kathryn	Baragwanath	Harvard University
Laura	Vedovato	Instituto de Pesquisas Tecnologicas
Laura	Duncanson	UMD
Lilian	Blanc	CIRAD
Loïc	Dutrieux	JRC
Luiz	Aragão	INPE
Marie-Therese	Schmehl	Universität Potsdam
Na	Chen	MIT
Nathália	Nacimento	University of São Paulo

Nilesh	Shine	University of Massachusetts Amherst
Olga	Nepomshina	GFZ
Pedro	Brancalion	University of São Paulo
Rene	Colditz	JRC
Ricardo	Dalagnol	JPL
Sascha	Delecluse	UCLouvain
Sugandha	Sugandha	University of Münster
Susan	Cook-Patton	Nature Conservancy
Thais	Michele	University of Exeter
Xueyuan	Gao	UMD
Yanlei	Feng	Berkeley

REFERENCES

1. Y. Pan, R. A. Birdsey, O. L. Phillips, R. A. Houghton, J. Fang, P. E. Kauppi, H. Keith, W. A. Kurz, A. Ito, S. L. Lewis, G.-J. Nabuurs, A. Shvidenko, S. Hashimoto, B. Lerink, D. Schepaschenko, A. Castanho, D. Murdiyarso, The enduring world forest carbon sink. *Nature* **631**, 563–569 (2024).

2. A. Baccini, W. Walker, L. Carvalho, M. Farina, D. Sulla-Menashe, R. A. Houghton, Tropical forests are a net carbon source based on aboveground measurements of gain and loss. *Science* **358**, 230–234 (2017).

3. W. Hubau, S. L. Lewis, O. L. Phillips, K. Affum-Baffoe, H. Beeckman, A. Cuní-Sanchez, A. K. Daniels, C. E. N. Ewango, S. Fauset, J. M. Mukinzi, D. Sheil, B. Sonké, M. J. P. Sullivan, T. C. H. Sunderland, H. Taedoumg, S. C. Thomas, L. J. T. White, K. A. Abernethy, S. Adu-Bredu, C. A. Amani, T. R. Baker, L. F. Banin, F. Baya, S. K. Begne, A. C. Bennett, F. Benedet, R. Bitariho, Y. E. Bocko, P. Boeckx, P. Boundja, R. J. W. Brienen, T. Brncic, E. Chezeaux, G. B. Chuyong, C. J. Clark, M. Collins, J. A. Comiskey, D. A. Coomes, G. C. Dargie, T. de Haulleville, M. N. D. Kamdem, J.-L. Doucet, A. Esquivel-Muelbert, T. R. Feldpausch, A. Fofanah, E. G. Foli, M. Gilpin, E. Gloor, C. Gonmadje, S. Gourlet-Fleury, J. S. Hall, A. C. Hamilton, D. J. Harris, T. B. Hart, M. B. N. Hockemba, A. Hladik, S. A. Ifo, K. J. Jeffery, T. Jucker, E. K. Yakusu, E. Kearsley, D. Kenfack, A. Koch, M. E. Leal, A. Levesley, J. A. Lindsell, J. Lisingo, G. Lopez-Gonzalez, J. C. Lovett, J.-R. Makana, Y. Malhi, A. R. Marshall, J. Martin, E. H. Martin, F. M. Mbayu, V. P. Medjibe, V. Mihindou, E. T. A. Mitchard, S. Moore, P. K. T. Munishi, N. N. Bengone, L. Ojo, F. E. Ondo, K. S.-H. Peh, G. C. Pickavance, A. D. Poulsen, J. R. Poulsen, L. Qie, J. Reitsma, F. Rovero, M. D. Swaine, J. Talbot, J. Taplin, D. M. Taylor, D. W. Thomas, B. Toirambe, J. T. Mukendi, D. Tuagben, P. M. Umunay, G. M. F. van der Heijden, H. Verbeeck, J. Vleminckx, S. Willcock, H. Wöll, J. T. Woods, L. Zemagho, Asynchronous carbon sink saturation in African and Amazonian tropical forests. *Nature* **579**, 80–87 (2020).

4. N. L. Harris, D. A. Gibbs, A. Baccini, R. A. Birdsey, S. de Bruin, M. Farina, L. Fatoyinbo, M. C. Hansen, M. Herold, R. A. Houghton, P. V. Potapov, D. R. Suarez, R. M. Roman-Cuesta, S. S. Saatchi, C. M. Slay, S. A. Turubanova, A. Tyukavina, Global maps of twenty-first century forest carbon fluxes. *Nat. Clim. Chang.* **11**, 234–240 (2021).

5. E. Berenguer, J. Ferreira, T. A. Gardner, L. E. O. C. Aragão, P. B. De Camargo, C. E. Cerri, M. Durigan, R. C. De Oliveira, I. C. G. Vieira, J. Barlow, A large-scale field assessment of carbon stocks in human-modified tropical forests. *Glob. Chang. Biol.* **20**, 3713–3726 (2014).

6. Y. Qin, X. Xiao, J. Wigneron, P. Ciais, M. Brandt, L. Fan, X. Li, S. Crowell, X. Wu, R. Doughty, Y. Zhang, F. Liu, S. Sitch, B. Moore, Carbon loss from forest degradation exceeds that from deforestation in the Brazilian Amazon. *Nat. Clim. Chang.* **11**, 442–448 (2021).

7. R. L. Chazdon, N. Blüthgen, P. H. S. Brancalion, V. Heinrich, F. Bongers, Drivers and benefits of natural regeneration in tropical forests. *Nat. Rev. Biodivers.* **1**, 298–314 (2025).

8. R. L. Chazdon, E. N. Broadbent, D. M. A. Rozendaal, F. Bongers, A. M. A. Zambrano, T. M. Aide, P. Balvanera, J. M. Becknell, V. Boukili, P. H. S. Brancalion, D. Craven, J. S. Almeida-Cortez, G. A. L. Cabral, B. de Jong, J. S. Denslow, D. H. Dent, S. J. DeWalt, J. M. Dupuy, S. M. Durán, M. M. Espírito-Santo, M. C. Fandino, R. G. César, J. S. Hall, J. L. Hernández-Stefanoni, C. C. Jakovac, A. B. Junqueira, D. Kennard, S. G. Letcher, M. Lohbeck, M. Martínez-Ramos, P. Massoca, J. A. Meave, R. Mesquita, F. Mora, R. Muñoz, R. Muscarella, Y. R. F. Nunes, S. Ochoa-Gaona, E. Orihuela-Belmonte, M. Peña-Claros, E. A. Pérez-García, D. Piotto, J. S. Powers, J. Rodríguez-Velazquez, I. E. Romero-Péréz, J. Ruíz, J. G. Saldarriaga, A. Sanchez-Azofeifa, N. B. Schwartz, M. K. Steininger, N. G. Swenson, M. Uriarte, M. van Breugel, H. van der Wal, M. D. M. Veloso, H. Vester, I. C. G. Vieira, T. V. Bentos, G. B. Williamson, L. Poorter, Carbon sequestration potential of second-growth forest regeneration in the Latin American tropics. *Sci. Adv.* **2**, e1501639 (2016).

9. S. C. Cook-Patton, S. M. Leavitt, D. Gibbs, N. L. Harris, K. Lister, K. J. Anderson-Teixeira, R. D. Briggs, R. L. Chazdon, T. W. Crowther, P. W. Ellis, H. P. Griscom, V. Herrmann, K. D. Holl, R. A. Houghton, C. Larrosa, G. Lomax, R. Lucas, P. Madsen, Y. Malhi, A. Paquette, J. D. Parker, K. Paul, D. Routh, S. Roxburgh, S. Saatchi, J. van den Hoogen, W. S. Walker, C. E. Wheeler, S. A. Wood, L. Xu, B. W. Griscom, Mapping carbon accumulation potential from global natural forest regrowth. *Nature* **585**, 545–550 (2020).

10. L. Poorter, F. Bongers, T. M. Aide, A. M. Almeyda Zambrano, P. Balvanera, J. M. Becknell, V. Boukili, P. H. S. Brancalion, E. N. Broadbent, R. L. Chazdon, D. Craven, J. S. De Almeida-Cortez, G. A. L. Cabral, B. H. J. De Jong, J. S. Denslow, D. H. Dent, S. J. DeWalt,

J. M. Dupuy, S. M. Durán, M. M. Espírito-Santo, M. C. Fandino, R. G. César, J. S. Hall, J. L. Hernandez-Stefanoni, C. C. Jakovac, A. B. Junqueira, D. Kennard, S. G. Letcher, J. C. Licona, M. Lohbeck, E. Marín-Spiotta, M. Martínez-Ramos, P. Massoca, J. A. Meave, R. Mesquita, F. Mora, R. Munõz, R. Muscarella, Y. R. F. Nunes, S. Ochoa-Gaona, A. A. De Oliveira, E. Orihuela-Belmonte, M. Penã-Claros, E. A. Pérez-Garciá, D. Piotto, J. S. Powers, J. Rodríguez-Velázquez, I. E. Romero-Pérez, J. Ruíz, J. G. Saldarriaga, A. Sanchez-Azofeifa, N. B. Schwartz, M. K. Steininger, N. G. Swenson, M. Toledo, M. Uriarte, M. Van Breugel, H. Van Der Wal, M. D. M. Veloso, H. F. M. Vester, A. Vicentini, I. C. G. Vieira, T. V. Bentos, G. B. Williamson, D. M. A. Rozendaal, Biomass resilience of Neotropical secondary forests. *Nature* **530**, 211–214 (2016).

11. V. H. A. Heinrich, R. Dalagnol, H. L. G. Cassol, T. M. Rosan, C. T. de Almeida, C. H. L. Silva Junior, W. A. Campanharo, J. I. House, S. Sitch, T. C. Hales, M. Adami, L. O. Anderson, L. E. O. C. Aragão, Large carbon sink potential of secondary forests in the Brazilian Amazon to mitigate climate change. *Nat. Commun.* **12**, 1785 (2021).

12. V. H. A. Heinrich, C. Vancutsem, R. Dalagnol, T. M. Rosan, D. Fawcett, C. H. L. Silva-Junior, H. L. G. Cassol, F. Achard, T. Jucker, C. A. Silva, J. House, S. Sitch, T. C. Hales, L. E. O. C. Aragão, The carbon sink of secondary and degraded humid tropical forests. *Nature* **615**, 436–442 (2023).

13. E. L. Bullock, C. E. Woodcock, C. Souza, P. Olofsson, Satellite-based estimates reveal widespread forest degradation in the Amazon. *Glob. Chang. Biol.* **26**, 2956–2969 (2020).

14. V. Heinrich, J. House, D. A. Gibbs, N. Harris, M. Herold, G. Grassi, R. Cantinho, T. M. Rosan, B. Zimbres, J. Z. Shimbo, J. Melo, T. Hales, S. Sitch, L. E. O. C. Aragão, Mind the gap: Reconciling tropical forest carbon flux estimates from earth observation and national reporting requires transparency. *Carbon Balance Manag.* **18**, (2023).

15. C. H. L. Silva Junior, L. E. O. C. Aragão, L. O. Anderson, M. G. Fonseca, Y. E. Shimabukuro, C. Vancutsem, F. Achard, R. Beuchle, I. Numata, C. A. Silva, E. E. Maeda, M. Longo, S. S. Saatchi, Persistent collapse of biomass in Amazonian forest edges following deforestation leads to unaccounted carbon losses. *Sci. Adv.* **6**, eaaz8360 (2020).

16. T. Neeff, J. G. P. Gamarra, A. Vollrath, E. Lindquist, G. Gill, J. Fox, J. Smith, K. Dyson, K. Tenneson, M. Sandker, T. Nakalema, Slowly getting there: A review of country experience on estimating emissions and removals from forest degradation. *Carbon Balance Manage.* **19**, 38 (2024).

17. D. M. Lapola, P. Pinho, J. Barlow, L. E. O. C. Aragão, E. Berenguer, R. Carmenta, H. M. Liddy, H. Seixas, C. V. J. Silva, C. H. L. Silva, A. A. C. Alencar, L. O. Anderson, D. Armenteras, V. Brovkin, K. Calders, J. Chambers, L. Chini, M. H. Costa, B. L. Faria, P. M. Fearnside, J. Ferreira, L. Gatti, V. H. Gutierrez-Velez, Z. Han, K. Hibbard, C. Koven, P. Lawrence, J. Pongratz, B. T. T. Portela, M. Rounsevell, A. C. Ruane, R. Schaldach, S. S. da Silva, C. von Randow, W. S. Walker, The drivers and impacts of Amazon forest degradation. *Science* **379**, eabp8622 (2023).

18. B. Buma, D. R. Gordon, K. M. Kleisner, A. Bartuska, A. Bidlack, R. DeFries, P. Ellis, P. Friedlingstein, S. Metzger, G. Morgan, K. Novick, J. N. Sanchirico, J. R. Collins, A. J. Eagle, R. Fujita, E. Holst, J. M. Lavallee, R. N. Lubowski, C. Melikov, L. A. Moore, E. E. Oldfield, J. Paltseva, A. M. Raffeld, N. A. Randazzo, C. Schneider, N. Uludere Aragon, S. P. Hamburg, Expert review of the science underlying nature-based climate solutions. *Nat. Clim. Chang.* **14**, 402–406 (2024).

19. G. D. Lennox, T. A. Gardner, J. R. Thomson, J. Ferreira, E. Berenguer, A. C. Lees, R. Mac Nally, L. E. O. C. Aragão, S. F. B. Ferraz, J. Louzada, N. G. Moura, V. H. F. Oliveira, R. Pardini, R. R. C. Solar, F. Z. Vaz-de Mello, I. C. G. Vieira, J. Barlow, Second rate or a second chance? Assessing biomass and biodiversity recovery in regenerating Amazonian forests. *Glob. Chang. Biol.* **24**, 5680–5694 (2018).

20. G. J. Nabuurs, P. Ciais, G. Grassi, R. A. Houghton, B. Sohngen, Reporting carbon fluxes from unmanaged forest. *Commun. Earth Environ.* **4**, 337 (2023).

21. L. Perugini, G. Pellis, G. Grassi, P. Ciais, H. Dolman, J. I. House, G. P. Peters, P. Smith, D. Günther, P. Peylin, Emerging reporting and verification needs under the Paris Agreement: How can the research community effectively contribute? *Environ. Sci. Policy* **122**, 116–126 (2021).

22. United Nations Framework Convention on Climate Change, “Synthesis report for the technical assessment component of the first global stocktake” (UNFCCC, 2022).

23. R. B. de Andrade, J. K. Balch, A. L. Parsons, D. Armenteras, R. Roman-Cuesta, J. Bulkan, Scenarios in tropical forest degradation: Carbon stock trajectories for REDD+. *Carbon Balance Manag.* **12**, 6 (2017).

24. Forest Declaration Assessment Partners, “Forests under fire: Tracking progress on 2030 forest goals” (2024); www.forestdeclaration.org.

25. S. de-Miguel, A. Birhanu, I. O. Menor, D. Ascoli, G. L. Spadoni, M. Erdozain, Carbon emissions from forest disturbances under global change. *Current Forest Rep.* **11**, 29 (2025).

26. C. Aquino, E. T. A. Mitchard, I. M. McNicol, H. Carstairs, A. Burt, B. L. Puma Vilca, M. Obiang Ebanéga, A. Modinga Dikongo, C. Dassi, S. Mayta, M. Tamayo, P. Grijalba, F. Miranda, M. Disney, Reliably mapping low-intensity forest disturbance using satellite radar data. *Front. For. Glob. Change* **5**, 1018762 (2022).

27. N. Sasaki, F. E. Putz, Critical need for new definitions of “forest” and “forest degradation” in global climate change agreements. *Conserv. Lett.* **2**, 226–232 (2009).

28. H. Allison, G. Goals, A. Holcomb, V. Kapos, “A discussion paper on the definition of forest degradation prepared for FAO” (2023); https://doi.org/10.13140/RG.2.2.21961.56169.

29. R. L. Chazdon, P. H. S. Brancalion, L. Laestadius, A. Bennett-Curry, K. Buckingham, C. Kumar, J. Moll-Rocek, I. C. G. Vieira, S. J. Wilson, When is a forest a forest? Forest concepts and definitions in the era of forest and landscape restoration. *Ambio* **45**, 538–550 (2016).

30. D. M. Lapola, P. Pinho, J. Barlow, L. E. O. C. Aragão, E. Berenguer, R. Carmenta, H. M. Liddy, H. Seixas, C. V. J. Silva, C. H. L. Silva, A. A. C. Alencar, L. O. Anderson, D. Armenteras, V. Brovkin, K. Calders, J. Chambers, L. Chini, M. H. Costa, B. L. Faria, P. M. Fearnside, J. Ferreira, L. Gatti, V. H. Gutierrez-Velez, Z. Han, K. Hibbard, C. Koven, P. Lawrence, J. Pongratz, B. T. T. Portela, M. Rounsevell, A. C. Ruane, R. Schaldach, S. S. da

Silva, C. von Randow, W. S. Walker, The drivers and impacts of Amazon forest degradation. *Science* **379**, eabp8622 (2023).

31. C. E. Doughty, D. B. Metcalfe, C. A. J. Girardin, F. F. Amézquita, D. G. Cabrera, W. H. Huasco, J. E. Silva-Espejo, A. Araujo-Murakami, M. C. Da Costa, W. Rocha, T. R. Feldpausch, A. L. M. Mendoza, A. C. L. Da Costa, P. Meir, O. L. Phillips, Y. Malhi, Drought impact on forest carbon dynamics and fluxes in Amazonia. *Nature* **519**, 78–82 (2015).

32. R. L. Chazdon, *Second Growth: The Promise of Tropical Forest Regeneration in an Age of Deforestation* (University of Chicago Press, 2014).

33. F. Bongers, R. L. Chazdon, L. Poorter, M. Peña-Claros, The potential of secondary forests. *Science* **348**, 642–643 (2015).

34. Y. Gao, M. Skutsch, J. Paneque-Gálvez, A. Ghilardi, Remote sensing of forest degradation: A review. *Environ. Res. Lett.* **15**, 2000–2010 (2020).

35. G.-J. Nabuurs, R. Mrabet, A. Abu Hatab, M. Bustamante, H. Clark, P. Havlik, J. House, C. Mbow, K. N. Ninan, A. Popp, S. Roe, B. Sohngen, S. Towprayoon, "Agriculture, Forestry and Other Land Uses (AFOLU)," in *IPCC, 2022: Climate Change 2022: Mitigation of Climate Change. Contribution of Working Group III to the Sixth Assessment Report of the Intergovernmental Panel on Climate Change*, P. R. Shukla, J. Skea, R. Slade, A. Al Khourdajie, R. van Diemen, D. McCollum, M. Pathak, S. SOme, P. Vyas, R. Fradera, M. Belkacemi, A. Hasija, G. Lisboa, S. Luz, J. Malley, Eds. (Cambridge University Press, 2022).

36. P. Friedlingstein, M. O'Sullivan, M. W. Jones, R. M. Andrew, J. Hauck, P. Landschützer, C. Le Quéré, H. Li, I. T. Luijkx, A. Olsen, G. P. Peters, W. Peters, J. Pongratz, C. Schwingshackl, S. Sitch, J. G. Canadell, P. Ciais, R. B. Jackson, S. R. Alin, A. Arneth, V. Arora, N. R. Bates, M. Becker, N. Bellouin, C. F. Berghoff, H. C. Bittig, L. Bopp, P. Cadule, K. Campbell, M. A. Chamberlain, N. Chandra, F. Chevallier, L. P. Chini, T. Colligan, J. Decayeux, L. Djeutchouang, X. Dou, C. Duran Rojas, K. Enyo, W. Evans, A. Fay, R. A. Feely, D. J. Ford, A. Foster, T. Gasser, M. Gehlen, T. Gkritzalis, G. Grassi, L. Gregor, N. Gruber, Ö. Gürses, I. Harris, M. Hefner, J. Heinke, G. C. Hurtt, Y. Iida, T. Ilyina, A. R. Jacobson, A. Jain, T. Jarníková, A. Jersild, F. Jiang, Z. Jin, E. Kato, R. F. Keeling, K. Klein

Goldewijk, J. Knauer, J. I. Korsbakken, S. K. Lauvset, N. Lefèvre, Z. Liu, J. Liu, L. Ma, S. Maksyutov, G. Marland, N. Mayot, P. McGuire, N. Metzl, N. M. Monacci, E. J. Morgan, S.-I. Nakaoka, C. Neill, Y. Niwa, T. Nützel, L. Olivier, T. Ono, P. I. Palmer, D. Pierrot, Z. Qin, L. Resplandy, A. Roobaert, T. M. Rosan, C. Rödenbeck, J. Schwinger, T. L. Smallman, S. Smith, R. Sospedra-Alfonso, T. Steinhoff, Q. Sun, A. J. Sutton, R. Séférian, S. Takao, H. Tatebe, H. Tian, B. Tilbrook, O. Torres, E. Tourigny, H. Tsujino, F. Tubiello, G. van der Werf, R. Wanninkhof, X. Wang, D. Yang, X. Yang, Z. Yu, W. Yuan, X. Yue, S. Zaehle, N. Zeng, J. Zeng, Global Carbon Budget 2024. *ESSD*, doi: 10.5194/essd-2024-519 (2024).

37. R. L. Chazdon, Tropical forest recovery: Legacies of human impact and natural disturbances. *Perspect. Plant Ecol. Evol. Syst.* **6**, 51–71 (2003).

38. T. M. Rosan, S. Sitch, L. M. Mercado, V. Heinrich, P. Friedlingstein, L. E. O. C. Aragão, Fragmentation-driven divergent trends in burned area in Amazonia and Cerrado. *Front. For. Glob. Chang.* **5**, 801408 (2022).

39. J. J. Gerwing, Degradation of forests through logging and fire in the eastern Brazilian Amazon. *For. Ecol. Manage.* **157**, 131–141 (2022).

40. W. Laurance, S. Laurance, L. Ferreira, J. Rankin-deMerona, C. Gascon, T. Lovejoy, Biomass collapse in Amazonian forest fragments. *Science* **278**, 1117–1118 (1997).

41. C. Bourgoin, G. Ceccherini, M. Girardello, C. Vancutsem, V. Avitabile, P. S. A. Beck, R. Beuchle, L. Blanc, G. Duveiller, M. Migliavacca, G. Vieilledent, A. Cescatti, F. Achard, Human degradation of tropical moist forests is greater than previously estimated. *Nature* **631**, 570–576 (2024).

42. L. E. O. C. Aragão, L. O. Anderson, M. G. Fonseca, T. M. Rosan, L. B. Vedovato, F. H. Wagner, C. V. J. Silva, C. H. L. Silva Junior, E. Arai, A. P. Aguiar, J. Barlow, E. Berenguer, M. N. Deeter, L. G. Domingues, L. Gatti, M. Gloor, Y. Malhi, J. A. Marengo, J. B. Miller, O. L. Phillips, S. Saatchi, 21st Century drought-related fires counteract the decline of Amazon deforestation carbon emissions. *Nat. Commun.* **9**, 536 (2018).

43. A. A. C. Alencar, L. A. Solórzano, D. C. Nepstad, Modeling forest understory fires in an eastern amazonian landscape. *Ecol. Appl.* **14**, 139–149 (2004).

44. H. Yang, P. Ciais, J.-P. Wigneron, J. Er^ Ome Chave, O. Cartus, X. Chen, L. Fan, J. K. Green, Y. Huang, E. Joetzjer, H. Kay, D. Makowski, F. Maignan, M. Santoro, S. Tao, L. Liu, Y. Yao, Climatic and biotic factors influencing regional declines and recovery of tropical forest biomass from the 2015/16 El Nino. doi: 10.1073/pnas (2022).

45. C. V. J. Silva, L. E. O. C. Araga, J. Barlow, F. Espirito-santo, P. J. Young, L. O. Anderson, E. Berenguer, I. Brasil, I. F. Brown, B. Castro, R. Farias, J. Ferreira, F. Franc, A. P. Lopes, C. Salimon, P. M. L. A. Grac, H. A. M. Xaud, Drought-induced Amazonian wildfires instigate a decadal-scale disruption of forest carbon dynamics. *Philos. Trans. R. Soc. Lond. B Biol. Sci.* **373**, 20180043 (2018).

46. Y. Feng, R. I. Negrón-Juárez, D. M. Romps, J. Q. Chambers, Amazon windthrow disturbances are likely to increase with storm frequency under global warming. *Nat. Commun.* **14**, 101 (2023).

47. N. Chen, N. E. Tsendbazar, D. Requena Suarez, J. Verbesselt, M. Herold, Characterizing aboveground biomass and tree cover of regrowing forests in Brazil using multi-source remote sensing data. *Remote Sens. Ecol. Conserv.* **9**, 553–567 (2023).

48. L. Poorter, D. Craven, C. C. Jakovac, M. T. van der Sande, L. Amissah, F. Bongers, R. L. Chazdon, C. E. Farrior, S. Kambach, J. A. Meave, R. Muñoz, N. Norden, N. Rüger, M. van Breugel, A. M. A. Zambrano, B. Amani, J. L. Andrade, P. H. S. Brancalion, E. N. Broadbent, H. de Foresta, D. H. Dent, G. Derroire, S. J. De Walt, J. M. Dupuy, S. M. Durán, A. C. Fantini, B. Finegan, A. Hernández-Jaramillo, J. L. Hernández-Stefanoni, P. Hietz, A. B. Junqueira, J. K. N'dja, S. G. Letcher, M. Lohbeck, R. López-Camacho, M. Martínez-Ramos, F. P. L. Melo, F. Mora, S. C. Müller, A. E. N'Guessan, F. Oberleitner, E. Ortiz-Malavassi, E. A. Pérez-García, B. X. Pinho, D. Piotto, J. S. Powers, S. Rodríguez-Buriticá, D. M. A. Rozendaal, J. Ruíz, M. Tabarelli, H. M. Teixeira, E. V. de Sá Barretto Sampaio, H. van der Wal, P. M. Villa, G. W. Fernandes, B. A. Santos, J. Aguilar-Cano, J. S. de Almeida-Cortez, E. Alvarez-Davila, F. Arreola-Villa, P. Balvanera, J. M. Becknell, G. A. L. Cabral, C. Castellanos-Castro, B. H. J. de Jong, J. E. Nieto, M. M. Espírito-Santo, M. C. Fandino, H.

García, D. García-Villalobos, J. S. Hall, A. Idárraga, J. Jiménez-Montoya, D. Kennard, E. Marín-Spiotta, R. Mesquita, Y. R. F. Nunes, S. Ochoa-Gaona, M. Peña-Claros, N. Pérez-Cárdenas, J. Rodríguez-Velázquez, L. S. Villanueva, N. B. Schwartz, M. K. Steininger, M. D. M. Veloso, H. F. M. Vester, I. C. G. Vieira, G. B. Williamson, K. Zanini, B. Hérault, Multidimensional tropical forest recovery. *Science* **374**, 1370–1376 (2021).

49. F. Q. Brearley, S. Prajadinata, P. S. Kidd, J. Proctor, Suriantata, Structure and floristics of an old secondary rain forest in Central Kalimantan, Indonesia, and a comparison with adjacent primary forest. *For. Ecol. Manage.* **195**, 385–397 (2004).

50. J. Chave, C. Piponiot, I. Maréchaux, H. de Foresta, D. Larpin, F. J. Fischer, G. Derroire, G. Vincent, B. Hérault, Slow rate of secondary forest carbon accumulation in the Guianas compared with the rest of the Neotropics. *Ecol. Appl.* **30**, e02004 (2020).

51. A. E. N'Guessan, J. K. N'dja, O. N. Yao, B. H. K. Amani, R. G. Z. Gouli, C. Piponiot, I. C. Zo-Bi, B. Hérault, Drivers of biomass recovery in a secondary forested landscape of West Africa. *For. Ecol. Manage.* **433**, 325–331 (2019).

52. T. Besisa Nguba, J. Bogaert, J. R. Makana, J. P. Mate Mweru, K. R. Sambieni, J. Bwazani Balandi, C. Mumbere Musavandalo, J. F. Bastin, Assessing forest degradation in the Congo Basin: The need to broaden the focus from logging to small-scale agriculture (a systematic review). *Forests* **16**, 953 (2025).

53. C. D. Philipson, M. E. J. Cutler, P. G. Brodric, G. P. Asne, D. S. Boy, P. M. Costa, J. Fiddes, G. M. Food, G. M. F. Van Der Heijden, A. Ledo, P. R. Lincol, J. A. Margrov, R. E. Marti, S. Milne, M. A. Pinar, G. Reynolds, M. Snoep, H. Tangki, Y. S. Wai, C. E. Wheele, D. F. R. P. Burslem, Active restoration accelerates the carbon recovery of human-modified tropical forests. *Science* **369**, 838–841 (2020).

54. J. Hu, J. Herbohn, R. L. Chazdon, J. Baynes, J. K. Vanclay, Above-ground biomass recovery following logging and thinning over 46 years in an Australian tropical forest. *Sci. Total Environ.* **734**, 139098 (2020).

55. C. C. Smith, J. Barlow, J. R. Healey, L. de Sousa Miranda, P. J. Young, N. B. Schwartz, Amazonian secondary forests are greatly reducing fragmentation and edge exposure in old-growth forests. *Environ. Res. Lett.* **18**, 124016 (2023).

56. B. H. K. Amani, A. E. N'Guessan, V. Van der Meersch, G. Derroire, C. Piponiot, A. G. M. Elogne, K. Traoré, J. K. N'Dja, B. Hérault, Lessons from a regional analysis of forest recovery trajectories in West Africa. *Environ. Res. Lett.* **17**, 115005 (2022).

57. K. J. Anderson-Teixeira, A. D. Miller, J. E. Mohan, T. W. Hudiburg, B. D. Duval, E. H. DeLucia, Altered dynamics of forest recovery under a changing climate. *Glob Change Biol*. (2013). https://doi.org/10.1111/gcb.12194.

58. Y. Su, X. Li, C. Zhang, W. Yan, P. Ciais, S. C. Cook-Patton, O. L. Phillips, J. Shang, A. Cescatti, J. M. Chen, J. Liu, J. Chave, C. E. Doughty, V. Heinrich, F. Tian, Y. Luo, Y. Liu, Z. Yu, D. Hao, S. Tao, Y. Zhang, Z. Zeng, R. Lafortezza, Y. Huang, L. Fan, X. Wang, Y. Qin, Q. Ran, K. Yan, X. Liu, L. Liu, Y. Yue, J. Ren, W. Yuan, X. Chen, Carbon accumulation rate peaks at 1,000-m elevation in tropical planted and regrowth forests. *One Earth* **8**, 101147 (2024).

59. E. A. Davidson, C. J. Reis De Carvalho, I. C. G. Vieira, R. D. O. Figueiredo, P. Moutinho, F. Y. Ishida, M. T. P. Dos Santos, J. B. Guerrero, K. Kalif, R. T. Sabá, Nitrogen and phosphorus limitation of biomass growth in a tropical secondary forest. *Ecol. Appl.* **14**, 150–163 (2004).

60. W. Tang, J. S. Hall, O. L. Phillips, R. J. W. Brienen, S. J. Wright, M. Y. Wong, L. O. Hedin, M. van Breugel, J. B. Yavitt, P. M. Hannam, S. A. Batterman, Tropical forest carbon sequestration accelerated by nitrogen. *Nat. Commun.* **17**, 55 (2026).

61. P. M. Fearnside, W. M. Guimarães, Carbon uptake by secondary forests in Brazilian Amazonia. *For. Ecol. Manage.* **80**, 35–46 (1996).

62. E. V. Wandelli, P. M. Fearnside, Secondary vegetation in central Amazonia: Land-use history effects on aboveground biomass. *For. Ecol. Manage.* **347**, 140–148 (2015).

63. A. R. Marshall, M. A. Coates, J. Archer, E. Kivambe, H. Mnendendo, S. Mtoka, R. Mwakisoma, R. J. R. L. de Figueiredo, F. M. Njilima, Liana cutting for restoring tropical forests: A rare palaeotropical trial. *Afr. J. Ecol.* **55**, 282–297 (2017).

64. M. J. P. Sullivan, S. L. Lewis, K. Affum-Baffoe, C. Castilho, F. Costa, A. C. Sanchez, C. E. N. Ewango, W. Hubau, B. Marimon, A. Monteagudo-Mendoza, L. Qie, B. Sonké, R. V. Martinez, T. R. Baker, R. J. W. Brienen, T. R. Feldpausch, D. Galbraith, M. Gloor, Y. Malhi, S. I. Aiba, M. N. Alexiades, E. C. Almeida, E. A. De Oliveira, E. Á. Dávila, P. A. Loayza, A. Andrade, S. A. Vieira, L. E. O. C. Aragão, A. Araujo-Murakami, E. J. M. M. Arets, L. Arroyo, P. Ashton, C. G. Aymard, F. B. Baccaro, L. F. Banin, C. Baraloto, P. B. Camargo, J. Barlow, J. Barroso, J. F. Bastin, S. A. Batterman, H. Beeckman, S. K. Begne, A. C. Bennett, E. Berenguer, N. Berry, L. Blanc, P. Boeckx, J. Bogaert, D. Bonal, F. Bongers, M. Bradford, F. Q. Brearley, T. Brncic, F. Brown, B. Burban, J. L. Camargo, W. Castro, C. Céron, S. C. Ribeiro, V. C. Moscoso, J. Chave, E. Chezeaux, C. J. Clark, F. C. De Souza, M. Collins, J. A. Comiskey, F. C. Valverde, M. C. Medina, L. Da Costa, M. Dančsák, G. C. Dargie, S. Davies, N. D. Cardozo, T. De Haulleville, M. B. De Medeiros, J. Del Aguila Pasquel, G. Derroire, A. Di Fiore, J. L. Doucet, A. Dourdain, V. Droissart, L. F. Duque, R. Ekoungoulou, F. Elias, T. Erwin, A. Esquivel-Muelbert, S. Fauset, J. Ferreira, G. F. Llampazo, E. Foli, A. Ford, M. Gilpin, J. S. Hall, K. C. Hamer, A. C. Hamilton, D. J. Harris, T. B. Hart, R. Hédl, B. Herault, R. Herrera, N. Higuchi, A. Hladik, E. H. Coronado, I. Huamantupa-Chuquimaco, W. H. Huasco, K. J. Jeffery, E. Jimenez-Rojas, M. Kalamandeen, M. N. K. Djuikouo, E. Kearsley, R. K. Umetsu, L. K. Kho, T. Killeen, K. Kitayama, B. Klitgaard, A. Koch, N. Labrière, W. Laurance, S. Laurance, M. E. Leal, A. Levesley, A. J. N. Lima, J. Lisingo, A. P. Lopes, G. Lopez-Gonzalez, T. Lovejoy, J. C. Lovett, R. Lowe, W. E. Magnusson, J. Malumbres-Olarte, Â. G. Manzatto, B. H. Marimon, A. R. Marshall, T. Marthews, S. M. D. A. Reis, C. Maycock, K. Melgaço, C. Mendoza, F. Metali, V. Mihindou, W. Milliken, E. T. A. Mitchard, P. S. Morandi, H. L. Mossman, L. Nagy, H. Nascimento, D. Neill, R. Nilus, P. N. Vargas, W. Palacios, N. P. Camacho, J. Peacock, C. Pendry, M. C. P. Mora, G. C. Pickavance, J. Pipoly, N. Pitman, M. Playfair, L. Poorter, J. R. Poulsen, A. D. Poulsen, R. Preziosi, A. Prieto, R. B. Primack, H. Ramírez-Angulo, J. Reitsma, M. Réjou-Méchain, Z. R. Correa, T. R. De Sousa, L. R. Bayona, A. Roopsind, A. Rudas, E. Rutishauser, K. A. Salim, R. P. Salomão, J. Schietti, D. Sheil, R. C. Silva, J. S. Espejo, C. S. Valeria, M. Silveira, M. Simo-Droissart, M. F. Simon, J. Singh, Y. C. S. Shareva, C. Stahl, J. Stropp, R. Sukri, T. Sunderland, M. Svátek, M.

D. Swaine, V. Swamy, H. Taedoumg, J. Talbot, J. Taplin, D. Taylor, H. Ter Steege, J. Terborgh, R. Thomas, S. C. Thomas, A. Torres-Lezama, P. Umunay, L. V. Gamarra, G. Van Der Heijden, P. Van Der Hout, P. Van Der Meer, M. Van Nieuwstadt, H. Verbeeck, R. Vernimmen, A. Vicentini, I. C. G. Vieira, E. V. Torre, J. Vleminckx, V. Vos, O. Wang, L. J. T. White, S. Willcock, J. T. Woods, V. Wortel, K. Young, R. Zagt, L. Zemagho, P. A. Zuidema, J. A. Zwerts, O. L. Phillips, Long-term thermal sensitivity of earth's tropical forests. *Science* **368**, 869–874 (2020).

65. A. W. Cheesman, F. Brown, P. Artaxo, M. N. Farha, G. A. Folberth, F. J. Hayes, V. H. A. Heinrich, T. C. Hill, L. M. Mercado, R. J. Oliver, M. O'Sullivan, J. Uddling, L. A. Cernusak, S. Sitch, Reduced productivity and carbon drawdown of tropical forests from ground-level ozone exposure. *Nat. Geosci.* **17**, 1003–1007 (2024).

66. E. F. Moran, E. S. Brondizio, J. M. Tucker, M. C. da Silva-Forsberg, S. Mccracken, I. Falesi, Effects of soil fertility and land-use on forest succession in Amazônia. *For. Ecol. Manage.* **139**, 93–108 (2000).

67. A. L. Giles, J. Schietti, M. F. Rosenfield, R. C. Mesquita, D. L. M. Vieira, I. C. G. Vieira, L. Poorter, P. H. S. Brancalion, M. Peña-Claros, J. Siqueira, L. Oliveira Junior, M. M. do Espírito-Santo, P. S. de M. Sarmento, J. N. Ferreira, E. Berenguer, J. Barlow, F. Elias, H. L. G. Cassol, R. C. Silva, S. C. Ribeiro, N. Medeiros, A. B. Junqueira, P. Massoca, M. J. Ferreira, M. Gastauer, L. V. Ferreira, D. R. A. de Almeida, L. E. O. C. Aragão, C. C. Jakovac, Simple ecological indicators benchmark regeneration success of Amazonian forests. *Commun. Earth Environ.* **5**, 780 (2024).

68. J. Chave, R. Condit, S. Aguilar, A. Hernandez, S. Lao, R. Perez, Error propagation and sealing for tropical forest biomass estimates. *Philos. Trans. R. Soc. Lond. Ser. B Biol. Sci.* **359**, 409–420 (2004).

69. IPCC, "Chapter 4. Forest land," in *IPCC Guidelines for National Greenhouse Gas Inventories*, Eggleston H.S, L. Buendia, K. Miwa, T. Ngara, K. Tanabe, Eds. (2006), vol. 4, pp. 1–29. www.ipcc-nggip.iges.or.jp/public/2006gl/pdf/4_Volume4/V4_04_Ch4_Forest_Land.pdf.

70. P. Olofsson, G. M. Foody, M. Herold, S. V. Stehman, C. E. Woodcock, M. A. Wulder, Good practices for estimating area and assessing accuracy of land change. *Remote Sens. Environ.* **148**, 42–57 (2014).

71. A. Tyukavina, S. V. Stehman, A. H. Pickens, P. Potapov, M. C. Hansen, Practical global sampling methods for estimating area and map accuracy of land cover and change. *Remote Sens. Environ.* **324**, 114714 (2025).

72. C. F. Salk, R. L. Chazdon, K. P. Andersson, Detecting landscape-level changes in tree biomass and biodiversity: Methodological constraints and challenges of plot-based approaches. *Can. J. For. Res.* **43**, 799–808 (2013).

73. F. Elias, J. Ferreira, A. F. Resende, E. Berenguer, F. França, C. C. Smith, G. Schwartz, R. O. Nascimento, M. Guedes, L. Chesini Rossi, M. M. M. de Seixas, C. M. da Silva, J. Barlow, Comparing contemporary and lifetime rates of carbon accumulation from secondary forests in the eastern Amazon. *For. Ecol. Manage.* **508**, 120053 (2022).

74. M. K. Nesha, M. Herold, V. De Sy, A. E. Duchelle, C. Martius, A. Branthomme, M. Garzuglia, O. Jonsson, A. Pekkarinen, An assessment of data sources, data quality and changes in national forest monitoring capacities in the Global Forest Resources Assessment 2005-2020. *Environ. Res. Lett.* **16**, 5 (2021).

75. A. Ahrends, M. T. Bulling, P. J. Platts, R. Swetnam, C. Ryan, N. Doggart, P. M. Hollingsworth, R. Marchant, A. Balmford, D. J. Harris, N. Gross-Camp, P. Sumbi, P. Munishi, S. Madoffe, B. Mhoro, C. Leonard, C. Bracebridge, K. Doody, V. Wilkins, N. Owen, A. R. Marshall, M. Schaafsma, K. Pfliegner, T. Jones, J. Robinson, E. Topp-Jørgensen, H. Brink, N. D. Burgess, Detecting and predicting forest degradation: A comparison of ground surveys and remote sensing in Tanzanian forests. *Plants People Planet* **3**, 268–281 (2021).

76. C. E. Wheeler, E. T. A. Mitchard, H. E. Nalasco Reyes, G. Iñiguez Herrera, J. I. Marquez Rubio, H. Carstairs, M. Williams, A new field protocol for monitoring forest degradation. *Front. For. Glob. Change* **4**, 655280 (2021).

77. K. Cueva, “Metodologia de Procesamiento Y Análisis de Datos del Inventario Forestal Nacional (IFN)” (San Lorenzo, 2015); https://estadisticasambientales.ine.gov.py/subidas/documentos/Manual de campo.pdf.

78. IPCC, *Good Practice Guidance for Land Use, Land-Use Change and Forestry* (Institute for Global Environmental Strategies for Intergovernmental Panel on Climate Change, 2003).

79. D. Requena Suarez, D. M. A. Rozendaal, V. De Sy, O. L. Phillips, E. Alvarez-Dávila, K. Anderson-Teixeira, A. Araujo-Murakami, L. Arroyo, T. R. Baker, F. Bongers, R. J. W. Brienen, S. Carter, S. C. Cook-Patton, T. R. Feldpausch, B. W. Griscom, N. Harris, B. Hérault, E. N. Honorio Coronado, S. M. Leavitt, S. L. Lewis, B. S. Marimon, A. Monteagudo Mendoza, J. Kassi N’dja, A. E. N’Guessan, L. Poorter, L. Qie, E. Rutishauser, P. Sist, B. Sonké, M. J. P. Sullivan, E. Vilanova, M. M. H. Wang, C. Martius, M. Herold, Estimating aboveground net biomass change for tropical and subtropical forests: Refinement of IPCC default rates using forest plot data. *Glob. Chang. Biol.* **25**, 3609–3624 (2019).

80. O. Csillik, M. Keller, M. Longo, A. Ferraz, E. R. Pinagé, E. B. Görgens, J. P. Ometto, V. Silgueiro, D. Brown, P. Duffy, K. C. Cushman, S. Saatchi, A large net carbon loss attributed to anthropogenic and natural disturbances in the Amazon Arc of Deforestation. *Proc. Natl. Acad. Sci. U.S.A.* **121**, e2310157121 (2024).

81. F. J. Fischer, B. Morgan, T. Jackson, J. Chave, D. Coomes, K. Cushman, R. Dalagnol, M. Dalponte, L. Duncanson, S. Saatchi, R. Seidl, K. Stereńczak, G. V. Laurin, S. Adu-Bredu, J. Aguirre-Gutiérrez, B. Antonielli, J. D. Armston, M. L. de Assis, N. Barbier, A. Burt, R. G. César, J. Cervenka, N. Coops, L. Cullen, J. W. Dalling, A. Davies, M. Demol, J. Ebenbeck, F. Fassnacht, L. Fatoyinbo, M. García, N. I. Gasparri, T. Gobakken, T. R. H. Goodbody, E. B. Görgens, T. Gorum, C. Gosper, H. Guan, J. Heiskanen, M. Heurich, M. Hobi, B. Höfle, A. Hooijer, A. Huth, A. Kedrov, J. R. Kellner, S. Koenig, K. Král, M. Krassovski, H. Kuechly, M. Krůček, K. K. Htoo, N. Labrière, D. Lai, J. Larson, H. Laudon, D. Lemke, J. Lenoir, Y. Malhi, O. A. Malik, M. Martin, I. McNicol, M. Milenkovic, D. Minor, E. Mitchard, V. Moudrý, H. C. Muller-Landau, E. Næsset, A. Nathalang, J. P. Ometto, M. Onishi, Y. Onoda, P. Pellikka, H. Persson, M. P. Ferreira, P. Ploton, S. M. Prober, F. Rahman, P. Rana, M. Réjou-Méchain, J. Schäfer, C. Senf, A. Shapiro, D. Schepaschenko, G. Shen, M. Silman, T.

Silva, J. Singh, F. Slik, J. Stillhard, A. Subash, R. Takeshige, S. Tao, E. Tenorio, T. Tokola, P. Tompalski, N. Tripathi, R. Valbuena, R. Valentini, R. Vernimmen, G. Vincent, J. Wallerman, W. S. W. M. Jaafar, Y. Wang, H. Weiser, J. White, L. Winiwarter, M. Wulder, Z. Yuan, K. Zdunic, Y. Zeng, H. Zhang, J. Zhang, Z. Zhang, T. Jucker, The Global Canopy Atlas: Analysis-ready maps of 3D structure for the world's woody ecosystems. biorXiv 673375 [Preprint] (2025). https://doi.org/10.1101/2025.08.31.673375.

82. M. Santoro, O. Cartus, N. Carvalhais, D. M. A. Rozendaal, V. Avitabile, A. Araza, S. De Bruin, M. Herold, S. Quegan, P. Rodríguez-Veiga, H. Balzter, J. Carreiras, D. Schepaschenko, M. Korets, M. Shimada, T. Itoh, Á. Moreno Martínez, J. Cavlovic, R. C. Gatti, P. Da Conceição Bispo, N. Dewnath, N. Labrière, J. Liang, J. Lindsell, E. T. A. Mitchard, A. Morel, A. M. Pacheco Pascagaza, C. M. Ryan, F. Slik, G. Vaglio Laurin, H. Verbeeck, A. Wijaya, S. Willcock, The global forest above-ground biomass pool for 2010 estimated from high-resolution satellite observations. *Earth Syst. Sci. Data* **13**, 3927–3950 (2021).

83. C. J. Iheaturu, G. F. Curatola Fernández, V. R. Wingate, F. O. Akinyemi, C. J. Okolie, C. Ifejika Speranza, Remote sensing of tropical forest recovery: A review and decision-support framework for multi-sensor integration. *Remote Sens. Environ.* **335**, 115257 (2026).

84. C. Vancutsem, F. Achard, J.-F. Pekel, G. Vieilledent, S. Carboni, D. Simonetti, J. Gallego, L. E. O. C. Aragão, R. Nasi, Long-term (1990–2019) monitoring of forest cover changes in the humid tropics. *Sci. Adv.* **7**, eabe1603 (2021).

85. D. R. A. de Almeida, L. B. Vedovato, M. Fuza, P. Molin, H. Cassol, A. F. Resende, P. M. Krainovic, C. T. de Almeida, C. Amaral, L. Haneda, R. W. Albuquerque, E. Gorgens, J. Romanelli, M. Ferreira, C. Salk, N. Espinoza, C. Silva, E. Broadbent, P. H. S. Brancalion, Remote sensing approaches to monitor tropical forest restoration: Current methods and future possibilities. *J Appl. Ecol*. (2024). https://doi.org/10.1111/1365-2664.14830.

86. J. Reiche, J. Balling, A. H. Pickens, R. N. Masolele, A. Berger, M. J. Weisse, D. Mannarino, Y. Gou, B. Slagter, G. Donchyts, S. Carter, Integrating satellite-based forest disturbance alerts improves detection timeliness and confidence. *Environ. Res. Lett.* **19**, 054011 (2024).

87. A. Holcomb, P. Burns, S. Keshav, D. A. Coomes, Repeat GEDI footprints measure the effects of tropical forest disturbances. *Remote Sens. Environ.* **308**, 114174 (2024).

88. A. Holcomb, S. V. Mathis, D. A. Coomes, S. Keshav, Computational tools for assessing forest recovery with GEDI shots and forest change maps. *Sci. Remote Sens.* **8**, 100106 (2023).

89. I. M. McNicol, C. M. Ryan, E. T. A. Mitchard, Carbon losses from deforestation and widespread degradation offset by extensive growth in African woodlands. *Nat. Commun.* **9**, 3045 (2018).

90. M. Liang, L. Duncanson, J. A. Silva, F. Sedano, Quantifying aboveground biomass dynamics from charcoal degradation in Mozambique using GEDI Lidar and Landsat. *Remote Sens. Environ.* **284**, 113367 (2023).

91. N. Chen, N. E. Tsendbazar, D. Requena Suarez, C. H. L. Silva-Junior, J. Verbesselt, M. Herold, Revealing the spatial variation in biomass uptake rates of Brazil's secondary forests. *ISPRS J. Photogramm. Remote Sens.* **208**, 233–244 (2024).

92. Y. Xu, P. Ciais, M. Santoro, C. Bourgoin, F. Ritter, A. Pellissier-Tanon, Y. Feng, C. Zhou, G. He, V. Heinrich, S. Besnard, N. Robinson, S. C. Cook-Patton, J. Chave, L. E. O. C. Aragao, J. P. Ometto, S. P. K. Bowring, I. Fayad, L. Zhu, Y. Su, J. P. Wigneron, W. Li, Small persistent humid forest clearings drive tropical forest biomass losses. *Nature* **649**, 375–380 (2026).

93. C. H. L. Silva Junior, V. H. A. Heinrich, A. T. G. Freire, I. S. Broggio, T. M. Rosan, J. Doblas, L. O. Anderson, G. X. Rousseau, Y. E. Shimabukuro, C. A. Silva, J. I. House, L. E. O. C. Aragão, Benchmark maps of 33 years of secondary forest age for Brazil. *Sci. Data* **7**, 269 (2020).

94. L. Zhu, W. Li, P. Ciais, J. He, A. Cescatti, M. Santoro, K. Tanaka, O. Cartus, Z. Zhao, Y. Xu, M. Sun, J. Wang, Comparable biophysical and biogeochemical feedbacks on warming from tropical moist forest degradation. *Nat. Geosci.* **16**, 244–249 (2023).

95. A. Araza, S. de Bruin, M. Herold, S. Quegan, N. Labriere, P. Rodriguez-Veiga, V. Avitabile, M. Santoro, E. T. A. Mitchard, C. M. Ryan, O. L. Phillips, S. Willcock, H. Verbeeck, J.

Carreiras, L. Hein, M. Schelhaas, A. M. Pacheco-Pascagaza, P. da Conceição Bispo, G. V. Laurin, G. Vieilledent, F. Slik, A. Wijaya, S. L. Lewis, A. Morel, J. Liang, H. Sukhdeo, D. Schepaschenko, J. Cavlovic, H. Gilani, R. Lucas, A comprehensive framework for assessing the accuracy and uncertainty of global above-ground biomass maps. *Remote Sens. Environ.* **272**, 112917 (2022).

96. I. M. McNicol, A. Keane, N. D. Burgess, S. J. Bowers, E. T. A. Mitchard, C. M. Ryan, Protected areas reduce deforestation and degradation and enhance woody growth across African woodlands. *Commun. Earth Environ.* **4**, 392 (2023).

97. M. C. Hansen, P. Potapov, A. Tyukavina, Comment on "Tropical forests are a net carbon source based on aboveground measurements of gain and loss". *Science* **3629**, eaar3629 (2019).

98. D. Requena Suarez, D. M. A. Rozendaal, V. De Sy, M. Decuyper, N. Málaga, P. Durán Montesinos, A. Arana Olivos, R. De la Cruz Paiva, C. Martius, M. Herold, Forest disturbance and recovery in Peruvian Amazonia. *Glob. Chang. Biol.* **29**, 3601–3621 (2023).

99. E. L. Bullock, S. P. Healey, Z. Yang, R. Acosta, H. Villalba, K. P. Insfrán, J. B. Melo, S. Wilson, L. Duncanson, E. Næsset, J. Armston, S. Saarela, G. Ståhl, P. L. Patterson, R. Dubayah, Estimating aboveground biomass density using hybrid statistical inference with GEDI lidar data and Paraguay's national forest inventory. *Environ. Res. Lett.* **18**, 085001 (2023).

100. C. V. J. Silva, L. E. O. C. Aragão, P. J. Young, F. Espirito-Santo, E. Berenguer, L. O. Anderson, I. Brasil, A. Pontes-Lopes, J. Ferreira, K. Withey, F. França, P. M. L. A. Graça, L. Kirsten, H. Xaud, C. Salimon, M. A. Scaranello, B. Castro, M. Seixas, R. Farias, J. Barlow, Estimating the multi-decadal carbon deficit of burned Amazonian forests. *Environ. Res. Lett.* **15**, 114023 (2020).

101. D. M. A. Rozendaal, R. L. Chazdon, Demographic drivers of tree biomass change during secondary succession in northeastern Costa Rica. *Ecol. Appl.* **25**, 506–516 (2015).

102. IPCC EFDB Management Group, "Final Report of the Emission Factor Database Review" (2025).

103. IPCC, "2019 Refinement to the 2006 IPCC Guidelines for National Greenhouse Gas Inventories Volume 4: Agriculture, Forestry, and Other Land Use" (2019); https://www.ipcc-nggip.iges.or.jp/public/2019rf/pdf/4_Volume4/19R_V4_Ch04_Forest Land.pdf.

104. L. Rowland, A. C. L. Da Costa, D. R. Galbraith, R. S. Oliveira, O. J. Binks, A. A. R. Oliveira, A. M. Pullen, C. E. Doughty, D. B. Metcalfe, S. S. Vasconcelos, L. V. Ferreira, Y. Malhi, J. Grace, M. Mencuccini, P. Meir, Death from drought in tropical forests is triggered by hydraulics not carbon starvation. *Nature* **528**, 119–122 (2015).

105. M. C. Hansen, P. V. Potapov, R. Moore, M. C. Hancher, S. A. Turubanova, A. Tyukavina, D. Thau, S. V. Stehman, S. J. Goetz, T. R. Loveland, A. Kommareddy, A. Egorov, L. Chini, C. O. Justice, J. R. G. Townshend, High-resolution global maps of 21st-century forest cover change. *Science* **850**, 2011–2014 (2013).

106. C. Dupuis, P. Lejeune, A. Michez, A. Fayolle, How can remote sensing help monitor tropical moist forest degradation?—A systematic review. *Remote Sens.* **12**, 1087 (2020).

107. A. H. Pickens, M. C. Hansen, Z. Song, A. Poulson, A. Komarova, A. Baggett, T. Kerr, A. Mikus, C. Ortiz Dominguez, A. Tyukavina, A. Lima, Rapid monitoring of global land change. *Nat. Commun.* **16**, 8948 (2025).

108. A. J. Welsink, C. Dupuis, L. Cue La Rosa, M. Weghorst, J. van der Zee, S. van der Woude, M. Peña-Claros, M. Herold, K. Fesenmyer, J. Reiche, Monitoring fine-scale natural and logging-related tropical forest degradation using Sentinel-1. *Remote Sens. Environ.* **328**, 114878 (2025).

109. R. Dalagnol, F. H. Wagner, L. S. Galvão, D. Braga, F. Osborn, L. B. Sagang, P. da Conceição Bispo, M. Payne, C. Silva Junior, S. Favrichon, V. Silgueiro, L. O. Anderson, L. E. O. C. Aragão, R. Fensholt, M. Brandt, P. Ciais, S. Saatchi, Mapping tropical forest degradation with deep learning and planet NICFI data. *Remote Sens. Environ.* **298**, 113798 (2023).

110. B. Slagter, J. Reiche, D. Marcos, A. Mullissa, E. Lossou, M. Peña-Claros, M. Herold, Monitoring direct drivers of small-scale tropical forest disturbance in near real-time with Sentinel-1 and -2 data. *Remote Sens. Environ.* **295**, 113655 (2023).

111. A. Mullissa, S. Saatchi, R. Dalagnol, T. Erickson, N. Provost, F. Osborn, A. Ashary, V. Moon, D. Melling, LUCA: A Sentinel-1 SAR-based global forest land use change alert. *Remote Sens.* **16**, 2151 (2024).

112. A. Tyukavina, P. Potapov, M. C. Hansen, A. H. Pickens, S. V. Stehman, S. Turubanova, D. Parker, V. Zalles, A. Lima, I. Kommareddy, X.-P. Song, L. Wang, N. Harris, Global trends of forest loss due to fire from 2001 to 2019. *Front. Remote Sens.* **3**, 825190 (2022).

113. E. Maurent, B. Hérault, C. Piponiot, G. Derroire, D. Delgado, B. Finegan, M. A. Kientz, B. H. K. Amani, M. A. N. Bieng, A common framework to model recovery in disturbed tropical forests: Common model for disturbed forest recovery. *Ecol. Model.* **483**, (2023).

114. D. M. Lapola, C. C. Blanco, B. R. Cardeli, A. Esquivel-Muelbert, J. V. Martinelli, C. A. N. Quesada, B. F. Rius, C. H. L. Silva-Junior, Not just semantics: CO_2 fertilization can be a disturbance leading to worldwide forest degradation. *Plants People Planet* **7**, 638–643 (2025).

115. J. Ghazoul, Z. Burivalova, J. Garcia-Ulloa, L. A. King, Conceptualizing forest degradation. *Trends Ecol. Evol.* **30**, 622–632 (2015).

116. J. Ghazoul, R. Chazdon, Annual Review of Environment and Resources Degradation and Recovery in Changing Forest Landscapes: A Multiscale Conceptual Framework. doi: 10.1146/annurev-environ (2017).

117. R. Neumann, "Stories of nature's hybridity in Europe: Implications for forest conservation in the Global South," in *The Social Lives of ForestsForests: Past, Present, and Future of Woodland Resurgence*, S. B. Hecht, K. D. Morrison, C. Padoch, Eds. (University of Chicago Press, 2014), pp. 31–44.

118. Brazil MCTI, "Brazil'sNational Forest Reference Emission Level for Results-based Payments for REDD+ under the United Nations Framework Convention on Climate Change" (2024); https://redd.unfccc.int/media/brazil-national-frel_modified_v3_clean-13-mar-2024.pdf.

119. B. Buma, P. C. Frumhoff, B. M. Rogers, S. Sartzetakis, C. A. Phillips, C. Schädel, R. Treharne, S. M. Natali, A. Alpert, M. Goldberg, J. P. Holdren, J. G. Canadell, K. Dooley, W. A. Kurz, C. Nobre, E. Ury, S. P. Hamburg, Policy solutions to better assess progress toward Paris goals given warming-induced ecosystem emissions, which shorten timelines by 2–5 years. *One Earth* **9**, 101571 (2026).

120. J. Tolan, H. I. Yang, B. Nosarzewski, G. Couairon, H. V. Vo, J. Brandt, J. Spore, S. Majumdar, D. Haziza, J. Vamaraju, T. Moutakanni, P. Bojanowski, T. Johns, B. White, T. Tiecke, C. Couprie, Very high resolution canopy height maps from RGB imagery using self-supervised vision transformer and convolutional decoder trained on aerial lidar. *Remote Sens. Environ.* **300**, 113888 (2024).

121. J. Balling, J. Verbesselt, V. De Sy, M. Herold, J. Reiche, Exploring archetypes of tropical fire-related forest disturbances based on dense optical and radar satellite data and active fire alerts. *Forests* **12**, 456 (2021).

122. D. A. Clark, D. B. Clark, S. F. Oberbauer, Annual tropical-rainforest productivity through two decades: Complex responses to climatic factors, [CO_2] and storm damage. *J. Geophys. Res. Biogeosci.* **126**, e2021JG006557 (2021).

123. I. D. Thompson, M. R. Guariguata, K. Okabe, C. Bahamondez, R. Nasi, V. Heymell, C. Sabogal, I. D. Thompson, M. R. Guariguata, K. Okabe, C. Bahamondez, R. Nasi, V. Heymell, C. Sabogal, An operational framework for defining and monitoring forest degradation. *Ecol. Soc.* **18**, 20 (2013).

124. J. L. Reid, M. E. Fagan, R. A. Zahawi, Positive site selection bias in meta-analyses comparing natural regeneration to active forest restoration. *Sci. Adv.* **4**, eaas9143 (2018).

125. D. R. A. de Almeida, A. M. Almeyda Zambrano, E. N. Broadbent, A. L. Wendt, P. Foster, B. E. Wilkinson, C. Salk, D. de A. Papa, S. C. Stark, R. Valbuena, E. B. Gorgens, C. A. Silva, P. H. S. Brancalion, M. Fagan, P. Meli, R. Chazdon, Detecting successional changes in tropical forest structure using GatorEye drone-borne lidar. *Biotropica* **52**, 1155–1167 (2020).

126. E. L. Bullock, C. E. Woodcock, Carbon loss and removal due to forest disturbance and regeneration in the Amazon. *Sci. Total Environ.* **764**, 142839 (2021).

127. M. S. De Meira Junior, J. R. R. Pinto, N. O. Ramos, E. P. Miguel, R. D. O. Gaspar, O. L. Phillips, The impact of long dry periods on the aboveground biomass in a tropical forests: 20 years of monitoring. *Carbon Balance Manag.* **15**, 12 (2020).

128. A. C. Bennett, G. C. Dargie, A. Cuni-Sanchez, J. T. Mukendi, W. Hubau, J. M. Mukinzi, O. L. Phillips, Y. Malhi, M. J. P. Sullivan, D. L. M. Cooper, S. Adu-Bredu, K. Affum-Baffoe, C. A. Amani, L. F. Banin, H. Beeckman, S. K. Begne, Y. E. Bocko, P. Boeckx, J. Bogaert, T. Brncic, E. Chezeaux, C. J. Clark, A. K. Daniels, T. de Haulleville, M.-N. D. Kamdem, J. Doucet, F. E. Ondo, C. E. N. Ewango, T. R. Feldpausch, E. G. Foli, C. Gonmadje, J. S. Hall, O. J. Hardy, D. J. Harris, S. A. Ifo, K. J. Jeffery, E. Kearsley, M. Leal, A. Levesley, J.-R. Makana, F. M. Lukasu, V. P. Medjibe, V. Mihindu, S. Moore, N. N. Begone, G. C. Pickavance, J. R. Poulsen, J. Reitsma, B. Sonké, T. C. H. Sunderland, H. Taedoumg, J. Talbot, D. S. Tuagben, P. M. Umunay, H. Verbeeck, J. Vleminckx, L. J. T. White, H. Woell, J. T. Woods, L. Zemagho, S. L. Lewis, Resistance of African tropical forests to an extreme climate anomaly. *Proc. Natl. Acad. Sci. U.S.A.* **118**, e2003169118 (2021).

129. M. B. Mills, Y. Malhi, R. M. Ewers, L. K. Kho, Y. A. Teh, S. Both, D. F. R. P. Burslem, N. Majalap, R. Nilus, W. Huaraca Huasco, R. Cruz, M. M. Pillco, E. C. Turner, G. Reynolds, T. Riutta, Tropical forests post-logging are a persistent net carbon source to the atmosphere. *Proc. Natl. Acad. Sci. U.S.A.* **120**, e2214462120 (2023).

130. M. Jung, C. Schwalm, M. Migliavacca, S. Walther, G. Camps-Valls, S. Koirala, P. Anthoni, S. Besnard, P. Bodesheim, N. Carvalhais, F. Chevallier, F. Gans, D. S. Goll, V. Haverd, P. Köhler, K. Ichii, A. K. Jain, J. Liu, D. Lombardozzi, J. E. M. S. Nabel, J. A. Nelson, M. O'Sullivan, M. Pallandt, D. Papale, W. Peters, J. Pongratz, C. Rödenbeck, S. Sitch, G.

Tramontana, A. Walker, U. Weber, M. Reichstein, Scaling carbon fluxes from eddy covariance sites to globe: Synthesis and evaluation of the FLUXCOM approach. *Biogeosciences* **17**, 1343–1365 (2020).

131. P. H. S. Brancalion, C. Bello, R. L. Chazdon, M. Galetti, P. Jordano, R. A. F. Lima, A. Medina, M. A. Pizo, J. L. Reid, Maximizing biodiversity conservation and carbon stocking in restored tropical forests. *Conserv. Lett*. (2018). https://doi.org/10.1111/conl.12454.

132. J.-F. Bastin, Y. Finegold, C. Garcia, D. Mollicone, M. Rezende, D. Routh, C. M. Zohner, T. W. Crowther, The global tree restoration potential. *Science* **365**, 76–79 (2019).

133. E. M. Gora, I. R. McGregor, H. C. Muller-Landau, J. C. Burchfield, K. C. Cushman, V. E. Rubio, G. B. Mori, M. J. P. Sullivan, M. W. Chmielewski, A. Esquivel-Muelbert, Storms are an important driver of change in tropical forests. *Ecol. Lett.* **28**, e70157 (2025).

134. E. M. Docherty, E. Gloor, D. Sponchiado, M. Gilpin, C. A. D. Pinto, H. M. Junior, I. Coughlin, L. Ferreira, J. A. S. Junior, A. C. L. da Costa, P. Meir, D. Galbraith, Long-term drought effects on the thermal sensitivity of Amazon forest trees. *Plant Cell Environ.* **46**, 185–198 (2023).

135. K. Nesha, M. Herold, V. De Sy, S. de Bruin, A. Araza, N. Málaga, J. G. P. Gamarra, K. Hergoualc'h, A. Pekkarinen, C. Ramirez, D. Morales-Hidalgo, R. Tavani, Exploring characteristics of national forest inventories for integration with global space-based forest biomass data. *Sci. Total Environ.* **850**, 157788 (2022).

136. F. N. Begliomini, P. H. S. Brancalion, "Are state-of-the-art LULC maps able to track ecological restoration efforts in Brazilian Atlantic Forest?," in *2024 IEEE International Geoscience and Remote Sensing Symposium* (IEEE, 2024), pp. 4748–4752.

137. B. Brede, L. Terryn, N. Barbier, H. M. Bartholomeus, R. Bartolo, K. Calders, G. Derroire, S. M. Krishna Moorthy, A. Lau, S. R. Levick, P. Raumonen, H. Verbeeck, D. Wang, T. Whiteside, J. van der Zee, M. Herold, Non-destructive estimation of individual tree biomass: Allometric models, terrestrial and UAV laser scanning. *Remote Sens. Environ.* **280**, 113180 (2022).

138. N. Robinson, C. R. Drever, D. A. Gibbs, K. Lister, A. Esquivel-Muelbert, V. Heinrich, P. Ciais, C. H. L. Silva-Junior, Z. Liu, T. A. M. Pugh, S. Saatchi, Y. Xu, S. C. Cook-Patton, Protect young secondary forests for optimum carbon removal. *Nat. Clim. Chang.* **15**, 793–800 (2025).

139. Y. Pan, R. A. Birdsey, J. Fang, R. Houghton, P. E. Kauppi, W. A. Kurz, O. L. Phillips, A. Shvidenko, S. L. Lewis, J. G. Canadell, P. Ciais, R. B. Jackson, S. W. Pacala, A. D. McGuire, S. Piao, A. Rautiainen, S. Sitch, D. Hayes, A large and persistent carbon sink in the world's forests. *Science* **333**, 988–993 (2011).

140. M. J. Grant, A. Booth, A typology of reviews: An analysis of 14 review types and associated methodologies. *Health Inf. Libr. J.* (2009). https://doi. org/10.1111/j.1471-1842.2009.00848.x.

141. A. R. Martin, M. Doraisami, S. C. Thomas, Global patterns in wood carbon concentration across the world's trees and forests. *Nat. Geosci.* **11**, 915–920 (2018).

142. F. E. Putz, T. Baker, B. W. Griscom, T. Gopalakrishna, A. Roopsind, P. M. Umunay, J. Zalman, E. A. Ellis, Ruslandi, P. W. Ellis, Intact forest in selective logging landscapes in the tropics. *Front. For. Glob. Change* **2**, 30 (2019).

143. C. V. de J. Silva, J. R. dos Santos, L. S. Galvão, R. D. da Silva, Y. M. Moura, Floristic and structure of an Amazonian primary forest and a chronosequence of secondary succession. *Acta Amaz.* **46**, 133–150 (2016).

144. R-core, TukeyHSD: Compute Tukey Honest Significant Differences (stats v3.6.3 package) (2019). https://rdocumentation.org/packages/stats/versions/3.6.2.

145. S. Mattan, hedges_g: Convert effect sizes (effectsize v 1.0.1 package) (2025). https://cran.r-project.org/web/packages/effectsize/effectsize.pdf.

146. R Development Core Team, R: A Language and Environment for Statistical Computing. R Foundation for Statistical Computing (2008). http://r-project.org/.

147. K. Baragwanath, E. Bayi, N. Shinde, Collective property rights lead to secondary forest growth in the Brazilian Amazon. *Proc. Natl. Acad. Sci. U.S.A.* **120**, e2221346120 (2023).

148. N. Imai, T. Seino, S.-i. Aiba, M. Takyu, J. Titin, K. Kitayama, Effects of selective logging on tree species diversity and composition of Bornean tropical rain forests at different spatial scales. *Plant Ecol.* **213**, 1413–1424 (2012).

149. J. K. Balch, P. M. Brando, D. C. Nepstad, M. T. Coe, D. Silvério, T. J. Massad, E. A. Davidson, P. Lefebvre, C. Oliveira-Santos, W. Rocha, R. T. S. Cury, A. Parsons, K. S. Carvalho, The susceptibility of southeastern Amazon forests to fire: Insights from a large-scale burn experiment. *Bioscience* **65**, 893–905 (2015).

150. Y. S. S. Barima, A. T. M. Kouakou, I. Bamba, Y. C. Sangne, M. Godron, J. Andrieu, J. Bogaert, Cocoa crops are destroying the forest reserves of the classified forest of Haut-Sassandra (Ivory Coast). *Glob. Ecol. Conserv.* **8**, 85–98 (2016).

151. C. C. Jakovac, A. B. Junqueira, R. Crouzeilles, M. Peña-Claros, R. C. G. Mesquita, F. Bongers, The role of land-use history in driving successional pathways and its implications for the restoration of tropical forests. *Biol. Rev. Camb. Philos. Soc.* **96**, 1114–1134 (2021).

152. D. Magnabosco Marra, S. E. Trumbore, N. Higuchi, G. H. P. M. Ribeiro, R. I. Negrón-Juárez, F. Holzwarth, S. W. Rifai, J. dos Santos, A. J. N. Lima, V. F. Kinupp, J. Q. Chambers, C. Wirth, Windthrows control biomass patterns and functional composition of Amazon forests. *Glob. Chang. Biol.* **24**, 5867–5881 (2018).

153. P. M. Brando, J. K. Balch, D. C. Nepstad, D. C. Morton, F. E. Putz, M. T. Coe, D. Silvério, M. N. Macedo, E. A. Davidson, C. C. Nóbrega, A. Alencar, B. S. Soares-Filho, Abrupt increases in Amazonian tree mortality due to drought-fire interactions. *Proc. Natl. Acad. Sci. U.S.A.* **111**, 6347–6352 (2014).

154. S. Traoré, I. C. Zo-Bi, C. Piponiot, R. Aussenac, B. Hérault, Fragmentation is the main driver of residual forest aboveground biomass in West African low forest-high deforestation landscapes. *Trees For. People* **15**, 100477 (2024).

155. A. Quisehuatl-Medina, J. P. Averett, B. A. Endress, L. Lopez-Toledo, Removal of cattle accelerates tropical dry forest succession in Northwestern Mexico. *Biotropica* **52**, 457–469 (2020).

156. P. van Lierop, E. Lindquist, S. Sathyapala, G. Franceschini, Global forest area disturbance from fire, insect pests, diseases and severe weather events. *For. Ecol. Manage.* **352**, 78–88 (2015).

157. S. Estrada-Villegas, P. R. Stevenson, O. López, S. J. Dewalt, L. S. Comita, D. H. Dent, Animal seed dispersal recovery during passive restoration in a forested landscape. *Philos. Trans. R Soc. Lond. B Biol. Sci.* **378**, 20210076 (2023).

158. O. Csillik, J. Reiche, V. De Sy, A. Araza, M. Herold, Rapid remote monitoring reveals spatial and temporal hotspots of carbon loss in Africa's rainforests. *Commun. Earth Environ.* **3**, 48 (2022).

159. B. A. Williams, H. L. Beyer, M. E. Fagan, R. L. Chazdon, M. Schmoeller, S. Sprenkle-Hyppolite, B. W. Griscom, J. E. M. Watson, A. M. Tedesco, M. Gonzalez-Roglich, G. A. Daldegan, B. Bodin, D. Celentano, S. J. Wilson, J. R. Rhodes, N. S. Alexandre, D. H. Kim, D. Bastos, R. Crouzeilles, Global potential for natural regeneration in deforested tropical regions. *Nature* **636**, 131–137 (2024).

160. L. Mo, C. M. Zohner, P. B. Reich, J. Liang, S. de Miguel, G. J. Nabuurs, S. S. Renner, J. van den Hoogen, A. Araza, M. Herold, L. Mirzagholi, H. Ma, C. Averill, O. L. Phillips, J. G. P. Gamarra, I. Hordijk, D. Routh, M. Abegg, Y. C. A. Yao, G. Alberti, A. M. A. Zambrano, B. V. Alvarado, E. Alvarez-Dávila, P. Alvarez-Loayza, L. F. Alves, I. Amaral, C. Ammer, C. Antón-Fernández, A. Araujo-Murakami, L. Arroyo, V. Avitabile, G. A. Aymard, T. R. Baker, R. Bałazy, O. Banki, J. G. Barroso, M. L. Bastian, J. F. Bastin, L. Birigazzi, P. Birnbaum, R. Bitariho, P. Boeckx, F. Bongers, O. Bouriaud, P. H. S. Brancalion, S. Brandl, F. Q. Brearley, R. Brienen, E. N. Broadbent, H. Bruelheide, F. Bussotti, R. Cazzolla Gatti, R. G. César, G. Cesljar, R. L. Chazdon, H. Y. H. Chen, C. Chisholm, H. Cho, E. Cienciala, C. Clark, D. Clark, G. D. Colletta, D. A. Coomes, F. Cornejo Valverde, J. J. Corral-Rivas, P. M. Crim, J. R. Cumming, S. Dayanandan, A. L. de Gasper, M. Decuyper, G. Derroire, B. DeVries, I. Djordjevic, J. Dolezal, A. Dourdain, N. L. E. Obiang, B. J. Enquist, T. J. Eyre, A. B.

Fandohan, T. M. Fayle, T. R. Feldpausch, L. V. Ferreira, L. Finér, M. Fischer, C. Fletcher, L. Frizzera, D. Gianelle, H. B. Glick, D. J. Harris, A. Hector, A. Hemp, G. Hengeveld, B. Hérault, J. L. Herbohn, A. Hillers, E. N. Honorio Coronado, C. Hui, T. Ibanez, N. Imai, A. M. Jagodziński, B. Jaroszewicz, V. K. Johannsen, C. A. Joly, T. Jucker, I. Jung, V. Karminov, K. Kartawinata, E. Kearsley, D. Kenfack, D. K. Kennard, S. Kepfer-Rojas, G. Keppel, M. L. Khan, T. J. Killeen, H. S. Kim, K. Kitayama, M. Köhl, H. Korjus, F. Kraxner, D. Kucher, D. Laarmann, M. Lang, H. Lu, N. V. Lukina, B. S. Maitner, Y. Malhi, E. Marcon, B. S. Marimon, B. H. Marimon-Junior, A. R. Marshall, E. H. Martin, J. A. Meave, O. Melo-Cruz, C. Mendoza, I. Mendoza-Polo, S. Miscicki, C. Merow, A. Monteagudo Mendoza, V. S. Moreno, S. A. Mukul, P. Mundhenk, M. G. Nava-Miranda, D. Neill, V. J. Neldner, R. V. Nevenic, M. R. Ngugi, P. A. Niklaus, J. Oleksyn, P. Ontikov, E. Ortiz-Malavasi, Y. Pan, A. Paquette, A. Parada-Gutierrez, E. I. Parfenova, M. Park, M. Parren, N. Parthasarathy, P. L. Peri, S. Pfautsch, N. Picard, M. T. F. Piedade, D. Piotto, N. C. A. Pitman, A. D. Poulsen, J. R. Poulsen, H. Pretzsch, F. R. Arevalo, Z. Restrepo-Correa, M. Rodeghiero, S. G. Rolim, A. Roopsind, F. Rovero, E. Rutishauser, P. Saikia, C. Salas-Eljatib, P. Saner, P. Schall, M. J. Schelhaas, D. Schepaschenko, M. Scherer-Lorenzen, B. Schmid, J. Schöngart, E. B. Searle, V. Seben, J. M. Serra-Diaz, D. Sheil, A. Z. Shvidenko, J. E. Silva-Espejo, M. Silveira, J. Singh, P. Sist, F. Slik, B. Sonké, A. F. Souza, K. J. Stereńczak, J. C. Svenning, M. Svoboda, B. Swanepoel, N. Targhetta, N. Tchebakova, H. ter Steege, R. Thomas, E. Tikhonova, P. M. Umunay, V. A. Usoltsev, R. Valencia, F. Valladares, F. van der Plas, T. Van Do, M. E. van Nuland, R. M. Vasquez, H. Verbeeck, H. Viana, A. C. Vibrans, S. Vieira, K. von Gadow, H. F. Wang, J. V. Watson, G. D. A. Werner, S. K. Wiser, F. Wittmann, H. Woell, V. Wortel, R. Zagt, T. Zawiła-Niedźwiecki, C. Zhang, X. Zhao, M. Zhou, Z. X. Zhu, I. C. Zo-Bi, G. D. Gann, T. W. Crowther, Integrated global assessment of the natural forest carbon potential. *Nature* **624**, 92–101 (2023).

161. V. Heinrich, A. Holcomb, S. Besnard, D. Requena Suarez, S. Cook-Patton, C. Bourgoin, R. Chazdon, D. Gibbs, F. Mendes, I. McNicol, C. Wheeler, C. Silva-Junior, B. Amani, J.-F. Bastin, T. Besisa Nguba, N. Chen, H Chen, P Ciais, R. Dalagnol, X. Dou, Q. Duan, X. Gao, A. N. Goodarzi, B. Hérault, J. House, D. Lapola, M. Liang, Z. Meng, G.-J. Nabuurs, E. Poor, L. Parsons, J. Reiche, S. Sitch, R. Valbuena, A.-J. Welsink, S. Wiltshire, C. Wu, Y. Xu, J. Zhao, L. Aragão, M. Herold (2026). Database for: A meta-analysis of carbon losses and gains from tropical moist forest degradation and regeneration. [Dataset] Zenodo (2026). https://doi.org/10.5281/zenodo.19470704.

162. F. Achard, H. D. Eva, P. Mayaux, H. J. Stibig, A. Belward, Improved estimates of net carbon emissions from land cover change in the tropics for the 1990s. *Global Biogeochem. Cycles* **18**, GB2008 (2004).

163. A. Alencar, D. Nepstad, M. Del Carmen, V. Diaz, “Forest Understory Fire in the Brazilian Amazon in ENSO and Non-ENSO Years: Area Burned and Committed Carbon Emissions” (2006); http://EarthInteractions.org.

164. D. S. Alves, J. V. Soares, S. Amaral, E. M. K. Mello, S. A. S. Almeida, O. F. Da Silva, A. M. Silveira, Biomass of primary and secondary vegetation in Rondônia, Western Brazilian Amazon. *Glob. Chang. Biol.* **3**, 451–461 (1997).

165. L. O. Anderson, L. E. O. C. Aragão, M. Gloor, E. Arai, M. Adami, S. S. Saatchi, Y. Malhi, Y. E. Shimabukuro, J. Barlow, E. Berenguer, V. Duarte, Disentangling the contribution of multiple land covers to fire-mediated carbon emissions in Amazonia during the 2010 drought. *Global Biogeochem. Cycles* **29**, 1739–1753 (2015).

166. D. R. Aryal, B. H. J. De Jong, S. Sánchez-Silva, A. Haas-Ek, L. Esparza-Olguin, S. Ochoa-Gaona, R. Ghimire, D. E. Morales-Ruiz, Biomass recovery along a tropical forest succession: Trends on tree diversity, wood traits and stand structure. *For. Ecol. Manage.* **555**, 121709 (2024).

167. G. P. Asner, P. G. Brodrick, C. Philipson, N. R. Vaughn, R. E. Martin, D. E. Knapp, J. Heckler, L. J. Evans, T. Jucker, B. Goossens, D. J. Stark, G. Reynolds, R. Ong, N. Renneboog, F. Kugan, D. A. Coomes, Mapped aboveground carbon stocks to advance forest conservation and recovery in Malaysian Borneo. *Biol. Conserv.* **217**, 289–310 (2018).

168. J. K. Balch, D. C. Nepstad, L. M. Curran, P. M. Brando, O. Portela, P. Guilherme, J. D. Reuning-Scherer, O. de Carvalho Jr., Size, species, and fire behavior predict tree and liana mortality from experimental burns in the Brazilian Amazon. *For. Ecol. Manage.* **261**, 68–77 (2011).

169. J. Barlow, C. A. Peres, Ecological responses to El Niño-induced surface fires in central Brazilian Amazonia: Management implications for flammable tropical forests. *Philos. Trans. R. Soc. Lond. B: Biol. Sci.* **359**, 367–380 (2004).

170. J. Barlow, C. A. Peres, B. O. Lagan, T. Haugaasen, Large tree mortality and the decline of forest biomass following Amazonian wildfires. *Ecol. Lett*. (2002). https://doi.org/10.1046/j.1461-0248.2003.00394.x.

171. J. Barlow, L. Parry, T. A. Gardner, J. Ferreira, L. E. O. C. Aragão, R. Carmenta, E. Berenguer, I. C. G. Vieira, C. Souza, M. A. Cochrane, The critical importance of considering fire in REDD+ programs. *Biol. Conserv.* **154**, 1–8 (2012).

172. M. Bauters, O. Vercleyen, B. Vanlauwe, J. Six, B. Bonyoma, H. Badjoko, W. Hubau, A. Hoyt, M. Boudin, H. Verbeeck, P. Boeckx, Long-term recovery of the functional community assembly and carbon pools in an African tropical forest succession. *Biotropica* **51**, 319–329 (2019).

173. E. Berenguer, G. D. Lennox, J. Ferreira, Y. Malhi, L. E. O. C. Aragão, J. R. Barreto, F. Del Bon Espírito-Santo, A. E. S. Figueiredo, F. França, T. A. Gardner, C. A. Joly, A. F. Palmeira, C. A. Quesada, L. C. Rossi, M. M. M. de Seixas, C. C. Smith, K. Withey, J. Barlow, Tracking the impacts of El Niño drought and fire in human-modified Amazonian forests. *Proc. Natl. Acad. Sci. U.S.A.* **118**, e2019377118 (2021).

174. N. J. Berry, O. L. Phillips, S. L. Lewis, J. K. Hill, D. P. Edwards, N. B. Tawatao, N. Ahmad, D. Magintan, C. V. Khen, M. Maryati, R. C. Ong, K. C. Hamer, The high value of logged tropical forests: Lessons from northern Borneo. *Biodivers. Conserv.* **19**, 985–997 (2010).

175. L. Blanc, M. Echard, B. Herault, D. Bonal, E. Marcon, J. Chave, C. Baraloto, Dynamics of aboveground carbon stocks in a selectively logged tropical forest. *Ecol. Appl.* **19**, 1397–1404 (2009).

176. M. T. L. Bonner, S. Schmidt, L. P. Shoo, A meta-analytical global comparison of aboveground biomass accumulation between tropical secondary forests and monoculture plantations. *For. Ecol. Manage.* **291**, 73–86 (2013).

177. Ministry of Foreign Affairs, Ministry of Science, Technology and Innovations, *Fourth Biennial Update Report of Brazil* (2020).

178. S. Brown, A. Lugo, Tropical secondary forests. *J. Trop. Ecol.* **6**, 1–32 (1990).

179. J. Bryan, P. Shearman, J. Ash, J. B. Kirkpatrick, Impact of logging on aboveground biomass stocks in lowland rain forest, Papua New Guinea. *Ecol. Appl.* **20**, 2096–2103 (2010).

180. T. Butarbutar, S. Soedirman, P. R. Neupane, M. Köhl, Carbon recovery following selective logging in tropical rainforests in Kalimantan, Indonesia. *For. Ecosyst.* **6**, 36 (2019).

181. J. M. B. Carreiras, J. Jones, R. M. Lucas, Y. E. Shimabukuro, Mapping major land cover types and retrieving the age of secondary forests in the Brazilian Amazon by combining single-date optical and radar remote sensing data. *Remote Sens. Environ.* **194**, 16–32 (2017).

182. H. L. G. Cassol, J. M. de B. Carreiras, E. C. Moraes, L. E. O. C. Aragão, C. V. de J. Silva, S. Quegan, Y. E. Shimabukuro, Retrieving secondary forest aboveground biomass from polarimetric ALOS-2 PALSAR-2 data in the Brazilian Amazon. *Remote Sens.* **11**, 59 (2019).

183. R. Chaplin-Kramer, I. Ramler, R. Sharp, N. M. Haddad, J. S. Gerber, P. C. West, L. Mandle, P. Engstrom, A. Baccini, S. Sim, C. Mueller, H. King, Degradation in carbon stocks near tropical forest edges. *Nat. Commun.* **6**, 10158 (2015).

184. M. A. Cochrane, M. D. Schulze, Fire as a recurrent event in tropical forests of the eastern Amazon: Effects on forest structure, biomass, and species composition. *Biotropica* **31**, 2–16 (1999).

185. A. Cuni Sanchez, J. A. Lindsell, The role of remnant trees in carbon sequestration, vegetation structure and tree diversity of early succession regrowing fallows in eastern Sierra Leone. *Afr. J. Ecol.* **55**, 188–197 (2016).

186. K. C. Cushman, J. T. Burley, B. Imbach, S. S. Saatchi, C. E. Silva, O. Vargas, C. Zgraggen, J. R. Kellner, Impact of a tropical forest blowdown on aboveground carbon balance. *Sci. Rep.* **11**, 11279 (2021).

187. M. E. J. Cutler, C. D. Philipson, D. F. R. P. Burslem, G. M. Foody, P. R. Lincol, M. A. Pinard, M. Snoep, C. E. Wheeler, H. Tangki, Y. S. Wai, Aboveground carbon density plots from a logged forest, Danum Valley, Borneo, 1992-2016 (Dataset), NERC Environmental

Information Data Centre (2020). https://doi.org/https://doi.org/10.5285/a75e6371-a931-4676-9199-d1f5af565ab2.

188. A. C. L. da Costa, D. Galbraith, S. Almeida, B. T. T. Portela, M. da Costa, J. de Athaydes Silva Junior, A. P. Braga, P. H. L. de Gonçalves, A. A. de Oliveira, R. Fisher, O. L. Phillips, D. B. Metcalfe, P. Levy, P. Meir, Effect of 7 yr of experimental drought on vegetation dynamics and biomass storage of an eastern Amazonian rainforest. *New Phytol.* **187**, 579–591 (2010).

189. A. L. de Avila, M. T. van der Sande, C. F. Dormann, M. Peña-Claros, L. Poorter, L. Mazzei, A. R. Ruschel, J. N. M. Silva, J. O. P. de Carvalho, J. Bauhus, Disturbance intensity is a stronger driver of biomass recovery than remaining tree-community attributes in a managed Amazonian forest. *J. Appl. Ecol.* **55**, 1647–1657 (2018).

190. V. De Sy, M. Herold, F. Achard, V. Avitabile, A. Baccini, S. Carter, J. G. P. W. Clevers, E. Lindquist, M. Pereira, L. Verchot, Tropical deforestation drivers and associated carbon emission factors derived from remote sensing data. *Environ. Res. Lett.* **14**, 094022 (2019).

191. E. L. Doyle, H. A. Graham, C. A. Boulton, T. M. Lenton, T. R. Feldpausch, A. M. Cunliffe, Evaluating GEDI for quantifying forest structure across a gradient of degradation in Amazonian rainforests. *Environ. Res. Lett.* **20**, 054016 (2025).

192. C. Dupuis, G. Ligot, J. F. Bastin, P. Lejeune, J. L. Doucet, V. Rossi, A. Fayolle, Scaling up the assessment of logging's impact on forest structure in Central Africa using field and UAV data. *Environ. Res. Lett.* **20**, 014018 (2025).

193. S. Escobar, F. L. Newell, M. J. Endara, J. E. Guevara-Andino, A. R. Landim, E. L. Neuschulz, R. Hausmann, J. Müller, K. M. Pedersen, M. Schleuning, C. J. Tremlett, E. Villa-Galaviz, H. M. Schaefer, D. A. Donoso, N. Blüthgen, Reassembly of a tropical rainforest: A new chronosequence in the Chocó tested with the recovery of tree attributes. *Ecosphere* **16**, e70157 (2025).

194. D. Faber-Langendoen, Ecological constraints on rain forest management at Bajo Calima, western Colombia. *Forest Ecol. Manage.* **53**, 213–244 (1992).

195. T. R. Feldpausch, M. A. Rondon, E. C. M. Fernandes, S. J. Riha, E. Wandelli, Carbon and nutrient accumulation in secondary forests regenerating on pastures in central Amazonia. *Ecol. Appl.* **14**, 164–176 (2004).

196. T. R. Feldpausch, S. Jirka, C. A. M. Passos, F. Jasper, S. J. Riha, When big trees fall: Damage and carbon export by reduced impact logging in southern Amazonia. *For. Ecol. Manage.* **219**, 199–215 (2005).

197. T. R. Feldpausch, C. da Conceicao Prates-Clark, E. C. M. Fernandes, S. J. Riha, Secondary forest growth deviation from chronosequence predictions in central Amazonia. *Glob. Chang. Biol.* **13**, 967–979 (2007).

198. A. M. E. S. Figueira, S. D. Miller, C. A. D. De Sousa, M. C. Menton, A. R. Maia, H. R. Da Rocha, M. L. Goulden, Effects of selective logging on tropical forest tree growth. *J. Geophys. Res. Biogeosci.* **114**, G00B05 (2009).

199. J. C. Fox, C. K. Yosi, P. Nimiago, F. Oavika, J. N. Pokana, K. Lavong, R. J. Keenan, Assessment of aboveground carbon in primary and selectively harvested tropical forest in Papua New Guinea. *Biotropica* **42**, 410–419 (2010).

200. S. Fujiki, S. Nishio, K. I. Okada, J. Nais, K. Kitayama, Plant communities and ecosystem processes in a succession-altitude matrix after shifting cultivation in the tropical montane forest zone of northern Borneo. *J. Trop. Ecol.* **33**, 33–49 (2017).

201. M. Fukushima, M. Kanzaki, M. Hara, T. Ohkubo, P. Preechapanya, C. Choocharoen, Secondary forest succession after the cessation of swidden cultivation in the montane forest area in Northern Thailand. *For. Ecol. Manage.* **255**, 1994–2006 (2008).

202. C. Gehring, M. Denich, P. L. G. Vlek, Resilience of secondary forest regrowth after slash-and-burn agriculture in central Amazonia. *J. Trop. Ecol.* **21**, 519–527 (2005).

203. J. J. Gerwing, Degradation of forests through logging and fire in the eastern Brazilian Amazon. *For. Ecol. Manage.* **157**, 131–141 (2002).

204. S. Gourlet-Fleury, F. Mortier, A. Fayolle, F. Baya, D. Ouédraogo, F. Bénédet, N. Picard, Tropical forest recovery from logging: A 24 year silvicultural experiment from Central Africa. *Philos. Trans. R. Soc. Lond. B Biol. Sci.* **368**, 20120302 (2013).

205. B. Griscom, P. Ellis, F. E. Putz, Carbon emissions performance of commercial logging in East Kalimantan, Indonesia. *Glob. Chang. Biol.* **20**, 923–937 (2014).

206. W. M. Guimarães, “Liberação de carbono e mudanças nos estoques dos nutrientes contidos na biomassa aérea e no solo resultante de queimadas de florestas secundárias em áreas de pastagens abandonadas,” thesis, Instituto Nacional de Pesquisas da Amazônia, Manaus (1993).

207. L. E. Haneda, P. H. S. Brancalion, D. Valle, C. A. Silva, E. B. Gorgens, G. A. Prata, R. A. Kamimura, S. H. M. Gomes, A. K. Sanchez, D. R. Alves de Almeida, Edge effect impacts on forest structure and carbon stocks in REDD+ projects: An assessment in the Amazon using UAV-LiDAR. *For. Ecol. Manage.* **585**, 122646 (2025).

208. T. Haugaasen, J. Barlow, C. A. Peres, Surface wildfires in central Amazonia: Short-term impact on forest structure and carbon loss. *For. Ecol. Manage.* **179**, 321–331 (2003).

209. R. A. Houghton, D. L. Skole, C. Nobre, J. Hackler, K. Lawrence, W. H. Chomentowsi, Annual fuxes of carbon from deforestation and regrowth in the Brazilian Amazon. *Nature* **403**, 301–304 (2000).

210. R. A. Houghton, W. Hole, W. Hole, Aboveground forest biomass and the global carbon balance. *Glob. Chang. Biol.* **11**, 945–958 (2005).

211. A. D. L. Houphouët, Y. C. Sangne, A. Diarrassouba, Y. C. Adou Yao, J. Betbeder, B. Hérault, Forest structure recovery around West Africa’s last great rainforest: Modelling complex dynamics in Taï national park. *Trees For. People* **22**, 101005 (2025).

212. M. Huang, G. P. Asner, Long-term carbon loss and recovery following selective logging in Amazon forests. *Global Biogeochem. Cycles* **24**, GB3028 (2010).

213. R. F. Hughes, J. B. Kauffman, V. J. Jaramillo, Biomass, carbon, and nutrient dynamics of secondary forests in a humid tropical region of Mexico. *Ecology* **80**, 1892–1907 (1999).

214. N. Jha, N. Kumar Tripathi, W. Chanthorn, W. Brockelman, A. Nathalang, R. Pelissier, S. Pimmasarn, P. Ploton, N. Sasaki, S. G. P. Virdis, M. Réjou-Méchain, Forest aboveground biomass stock and resilience in a tropical landscape of Thailand. *Biogeosciences* **17**, 121–134 (2020).

215. C. M. Johnson, I. C. G. Vieira, D. J. Zarin, J. Frizano, A. H. Johnson, Carbon and nutrient storage in primary and secondary forests in eastern Amazônia. *For. Ecol. Manage.* **147**, 245–252 (2001).

216. M. Kalamandeen, E. Gloor, I. Johnson, S. Agard, M. Katow, A. Vanbrooke, D. Ashley, S. A. Batterman, G. Ziv, K. Holder-Collins, O. L. Phillips, E. S. Brondizio, I. Vieira, D. Galbraith, Limited biomass recovery from gold mining in Amazonian forests. *J. Appl. Ecol.* **57**, 1730–1740 (2020).

217. L. Kenne Tene, M. C. Momo Solefack, S. Momo Takoudjou, S. R. Monthe, A. Tchokomeni, Forest activities drive diversity and structure in the tropical rainforest. *Afr. J. Ecol.* **61**, 689–698 (2023).

218. T. Kenzo, R. Furutani, D. Hattori, S. Tanaka, K. Sakurai, I. Ninomiya, J. J. Kendawang, Aboveground and belowground biomass in logged-over tropical rain forests under different soil conditions in Borneo. *J. For. Res.* **20**, 197–205 (2015).

219. R. D. Lasco, O. M. Lopez Center, K. Macdicken, F. Pulhin, Carbon stocks assessment of a selectively logged Dipterocarp forest and wood processing mill in the Philippines. *J. Trop. For. Sci.* **18**, 212–221 (2006).

220. D. Lawrence, Biomass accumulation after 10-200 years of shifting cultivation in Bornean rain forest. *Ecology* **86**, 26–33 (2005).

221. S. G. Letcher, R. L. Chazdon, Rapid recovery of biomass, species richness, and species composition in a forest chronosequence in Northeastern Costa Rica. *Biotropica* **41**, 608–617 (2009).

222. M. Longo, M. Keller, M. N. dos-Santos, V. Leitold, E. R. Pinagé, A. Baccini, S. Saatchi, E. M. Nogueira, M. Batistella, D. C. Morton, Aboveground biomass variability across intact and degraded forests in the Brazilian Amazon. *Global Biogeochem. Cycles* **30**, 1639–1660 (2016).

223. I. A. Makelele, K. Verheyen, P. Boeckx, L. Cizungu Ntaboba, B. Mujinya Bazirake, C. Ewango, M. Bauters, Afrotropical secondary forests exhibit fast diversity and functional recovery, but slow compositional and carbon recovery after shifting cultivation. *J. Veg. Sci.* **32**, e13071 (2021).

224. L. Mazzei, P. Sist, A. Ruschel, F. E. Putz, P. Marco, W. Pena, J. E. R. Ferreira, Above-ground biomass dynamics after reduced-impact logging in the Eastern Amazon. *For. Ecol. Manage.* **259**, 367–373 (2010).

225. V. P. Medjibe, F. E. Putz, M. P. Starkey, A. A. Ndouna, H. R. Memiaghe, Impacts of selective logging on above-ground forest biomass in the Monts de Cristal in Gabon. *For. Ecol. Manage.* **262**, 1799–1806 (2011).

226. V. Medjibe, F. Putz, Cost comparisons of reduced-impact and conventional logging in the tropics. *J. For. Econ.* **18**, 242–256 (2012).

227. V. P. Medjibe, F. E. Putz, C. Romero, Certified and uncertified logging concessions compared in Gabon: Changes in stand structure, tree species, and biomass. *Environ. Manag.* **51**, 524–540 (2013).

228. O. O. Michel, Y. Yu, W. Fan, T. Lubalega, C. Chen, C. K. Sudi Kaiko, Impact of land use change on tree diversity and aboveground carbon storage in the Mayombe Tropical Forest of the Democratic Republic of Congo. *Land* **11**, 787 (2022).

229. S. D. Miller, M. L. Goulden, L. R. Hutyra, M. Keller, S. R. Saleska, S. C. Wofsy, A. M. S. Figueira, H. R. Da Rocha, P. B. De Camargo, Reduced impact logging minimally alters tropical rainforest carbon and energy exchange. *Proc. Natl. Acad. Sci. U.S.A.* **108**, 19431–19435 (2011).

230. S. E. Mokake, G. B. Chuyong, E. A. Egbe, Long-term impacts of selective logging in a tropical rainforest in the east region of Cameroon. *Afr. J. Ecol.* **62**, e13341 (2024).

231. S. Armenta Montero, E. A. Ellis, Carbon stock recovery from tree regeneration following selective logging in tropical forest of the Yucatan Peninsula, Mexico. *Carbon Manag.* **14**, 2238672 (2023).

232. P. C. J. Moonen, B. Verbist, F. Boyemba Bosela, L. Norgrove, S. Dondeyne, K. Van Meerbeek, E. Kearsley, H. Verbeeck, P. Vermeir, P. Boeckx, B. Muys, Disentangling how management affects biomass stock and productivity of tropical secondary forests fallows. *Sci. Total Environ.* **659**, 101–114 (2019).

233. J. D. U. Muñoz, D. M. Marra, R. I. Negrón-Juarez, R. Tello-Espinoza, W. Alegría-Muñoz, T. Pacheco-Gómez, S. W. Rifai, J. Q. Chambers, H. S. Jenkins, A. Brenning, S. E. Trumbore, Recovery of forest structure following large-scale windthrows in the northwestern amazon. *Forests* **12**, 667 (2021).

234. A. Murali, S. Kasinathan, K. Bhat, J. Ratnam, M. Sankaran, D. Mudappa, T. R. S. Raman, A. M. Osuri, Structure and dynamics of secondary and mature rainforests: Insights from South Asian long-term monitoring plots. *Plant Ecol. Divers.* **18**, 55–67 (2025).

235. H. T. Murphy, M. G. Bradford, A. Dalongeville, A. J. Ford, D. J. Metcalfe, No evidence for long-term increases in biomass and stem density in the tropical rain forests of Australia. *J. Ecol.* **101**, 1589–1597 (2013).

236. M. V. Neves d'Oliveira, R. P. Miller, L. C. Oliveira, E. M. Braz, F. Thaines, J. L. Januário, M. H. A. Acuña, Growth dynamics of an Amazonian forest: Effects of reduced impact logging and recurring atypical climate events during a 20-year study. *For. Ecol. Manage.* **562**, 121937 (2024).

237. I. Numata, M. A. Cochrane, C. M. Souza, M. H. Sales, Carbon emissions from deforestation and forest fragmentation in the Brazilian Amazon. *Environ. Res. Lett.* **6**, 044003 (2011).

238. F. Oberleitner, C. Egger, S. Oberdorfer, S. Dullinger, W. Wanek, P. Hietz, Recovery of aboveground biomass, species richness and composition in tropical secondary forests in SW Costa Rica. *For. Ecol. Manage.* **479**, 118580 (2021).

239. T. Ohtsuka, Biomass changes in early tropical succession on a large-scale shifting cultivation area, northeast Borneo Island. *Tropics* **10**, 529 (2001).

240. S. Ojoatre, J. Barlow, S. R. Jacobs, M. C. Rufino, Recovery of aboveground biomass, soil carbon stocks and species diversity in tropical montane secondary forests of East Africa. *For. Ecol. Manage.* **552**, 121569 (2024).

241. T. R. H. Pearson, S. Brown, F. M. Casarim, Carbon emissions from tropical forest degradation caused by logging. *Environ. Res. Lett.* **9**, 034017 (2014).

242. D. P. Abadía, H. Q. Mosquera, J. M. Del Arco, Influence of environmental factors on the aboveground biomass of mature and postmining forests in Chocó. *Life* **15**, 98 (2025).

243. O. L. Phillips, L. E. O. C. Aragão, S. L. Lewis, J. B. Fisher, J. Lloyd, G. López-González, Y. Malhi, A. Monteagudo, J. Peacock, C. A. Quesada, G. van der Heijden, S. Almeida, I. Amaral, L. Arroyo, G. Aymard, T. R. Baker, O. Bánki, L. Blanc, D. Bonal, P. Brando, J. Chave, A. C. A. de Oliveira, N. D. Cardozo, C. I. Czimczik, T. R. Feldpausch, M. A. Freitas, E. Gloor, N. Higuchi, E. Jiménez, G. Lloyd, P. Meir, C. Mendoza, A. Morel, D. A. Neill, D. Nepstad, S. Patiño, M. C. Peñuela, A. Prieto, F. Ramírez, M. Schwarz, J. Silva, M. Silveira, A. S. Thomas, H. ter Steege, J. Stropp, R. Vásquez, P. Zelazowski, E. A. Dávila, S. Andelman, A. Andrade, K. Chao, T. Erwin, A. Di Fiore, E. Honorio C, H. Keeling, T. J. Killeen, W. F. Laurance, A. P. Cruz, N. C. A. Pitman, P. N. Vargas, H. Ramírez-Angulo, A. Rudas, R. Salamão, N. Silva, J. Terborgh, A. Torres-Lezama, Drought sensitivity of the Amazon rainforest. *Science* **323**, 1344–1347 (2009).

244. E. R. Pinagé, M. Keller, P. Duffy, M. Longo, M. N. Dos-Santos, D. C. Morton, Long-term impacts of selective logging on amazon forest dynamics from multi-temporal airborne lidar. *Remote Sens.* **11**, 709 (2019).

245. M. A. Pinard, F. E. Putz, Retaining forest biomass by reducing logging damage. *Biotropica* **28**, 278–295 (1996).

246. A. Pontes-Lopes, C. V. J. Silva, J. Barlow, L. M. Rincón, W. A. Campanharo, C. A. Nunes, C. T. De Almeida, C. H. L. Silva Júnior, H. L. G. Cassol, R. Dalagnol, S. C. Stark, P. M. L. A. Graça, L. E. O. C. Aragão, Drought-driven wildfire impacts on structure and dynamics in a wet Central Amazonian forest. *Proc. Biol. Sci.* **288**, 20210094 (2021).

247. D. I. Rappaport, D. C. Morton, M. Longo, M. Keller, R. Dubayah, M. N. Dos-Santos, Quantifying long-term changes in carbon stocks and forest structure from Amazon forest degradation. *Environ. Res. Lett.* **13**, 065013 (2018).

248. C. A. Rockwell, K. A. Kainer, M. V. N. d'Oliveira, C. L. Staudhammer, C. Baraloto, Logging in bamboo-dominated forests in southwestern Amazonia: Caveats and opportunities for smallholder forest management. *For. Ecol. Manage.* **315**, 202–210 (2014).

249. C. H. Rodríguez-León, A. Sterling, A. Trujillo-Briñez, Y. D. Suárez-Córdoba, L. L. Roa-Fuentes, Forest attribute dynamics in secondary forests: Insights for advancing ecological restoration and transformative territorial management in the Amazon. *Diversity* **17**, 39 (2025).

250. A. Roopsind, V. Wortel, W. Hanoeman, F. E. Putz, Quantifying uncertainty about forest recovery 32-years after selective logging in Suriname. *For. Ecol. Manage.* **391**, 246–255 (2017).

251. D. M. A. Rozendaal, R. L. Chazdon, F. Arreola-Villa, P. Balvanera, T. V. Bentos, J. M. Dupuy, J. L. Hernández-Stefanoni, C. C. Jakovac, E. E. Lebrija-Trejos, M. Lohbeck, M. Martínez-Ramos, P. E. S. Massoca, J. A. Meave, R. C. G. Mesquita, F. Mora, E. A. Pérez-García, I. E. Romero-Pérez, I. Saenz-Pedroza, M. van Breugel, G. B. Williamson, F. Bongers, Demographic drivers of aboveground biomass dynamics during secondary succession in neotropical dry and wet forests. *Ecosystems* **20**, 340–353 (2017).

252. E. Rutishauser, B. Hérault, C. Baraloto, L. Blanc, L. Descroix, E. D. Sotta, J. Ferreira, M. Kanashiro, L. Mazzei, M. V. N. D'Oliveira, L. C. De Oliveira, M. Peña-Claros, F. E. Putz,

A. R. Ruschel, K. Rodney, A. Roopsind, A. Shenkin, K. E. Da Silva, C. R. De Souza, M. Toledo, E. Vidal, T. A. P. West, V. Wortel, P. Sist, Rapid tree carbon stock recovery in managed Amazonian forests. *Curr. Biol.* **25**, R787–R788 (2015).

253. P. Saner, Y. Y. Loh, R. C. Ong, A. Hector, Carbon stocks and fluxes in tropical lowland dipterocarp rainforests in Sabah, Malaysian Borneo. *PLOS ONE* **7**, e29642 (2012).

254. W. L. Silver, R. Ostertag, A. E. Lugo, The potential for carbon sequestration through reforestationof abandoned tropical agricultural and pasture lands. *Restor. Ecol.* **8**, 394–407 (2000).

255. D. V. Silvério, P. M. Brando, M. M. C. Bustamante, F. E. Putz, D. M. Marra, S. R. Levick, S. E. Trumbore, Fire, fragmentation, and windstorms: A recipe for tropical forest degradation. *J. Ecol.* **107**, 656–667 (2019).

256. J. W. F. Slik, C. S. Bernard, M. Van Beek, F. C. Breman, K. A. O. Eichhorn, Tree diversity, composition, forest structure and aboveground biomass dynamics after single and repeated fire in a Bornean rain forest. *Oecologia* **158**, 579–588 (2008).

257. S. M. Stas, T. C. Le, H. D. Tran, T. T. H. Hoang, M. van Kuijk, A. Van Le, D. T. Ngo, A. van Oostrum, O. L. Phillips, E. Rutishauser, B. D. Spracklen, T. T. A. Tran, T. T. Le, D. V. Spracklen, Logging intensity drives variability in carbon stocks in lowland forests in Vietnam. *For. Ecol. Manage.* **460**, 117863 (2020).

258. M. K. Steininger, Secondary forest structure and biomass following short and extended land-use in central and southern Amazonia. *J. Trop. Ecol.* **16**, 689–708 (2000).

259. P. N. Titenwi, M. N. Sainge, U. Kargbo, R. A. S. Kamara, A. Musa, T. M. Kabba, B. K. Buanie, A. L. Njouonkou, E. Aruna, M. J. P. Sullivan, A. Leite, A. Cuni-Sanchez, Carbon recovery in secondary forests: Insights from three West African countries. *For. Ecol. Manage.* **575**, 122386 (2025).

260. C. Uhl, R. Buschbacher, E. A. S. Serrão, Abandoned pastures in eastern Amazonia. I. Patterns of plant succession. *J. Ecol.* **76**, 663–681 (1988).

261. P. M. Umunay, T. G. Gregoire, T. Gopalakrishna, P. W. Ellis, F. E. Putz, Selective logging emissions and potential emission reductions from reduced-impact logging in the Congo Basin. *For. Ecol. Manage.* **437**, 360–371 (2019).

262. M. Uriarte, J. R. Lasky, V. K. Boukili, R. L. Chazdon, A trait-mediated, neighbourhood approach to quantify climate impacts on successional dynamics of tropical rainforests. *Funct. Ecol.* **30**, 157–167 (2016).

263. T. Van Do, A. Osawa, N. T. Thang, Recovery process of a mountain forest after shifting cultivation in Northwestern Vietnam. *For. Ecol. Manage.* **259**, 1650–1659 (2010).

264. E. Vidal, T. A. P. West, F. E. Putz, Recovery of biomass and merchantable timber volumes twenty years after conventional and reduced-impact logging in Amazonian Brazil. *For. Ecol. Manage.* **376**, 1–8 (2016).

265. J. Vijayanathan, M. A. Lias, M. S. A. M. Yusof, M. Z. Abdullah, T. Van Do, W. R. Kadir, Net ecosystem production of a tropical secondary forest in Jengka, Pahang, Malaysia. *IForest* **18**, 54–60 (2025).

266. D. Wanyama, M. C. Wimberly, R. Doughty, F. Mensah, Forest aboveground biomass accumulation lags forest canopy recovery in degraded tropical forests of Ghana. *Int. J. Appl. Earth Obs. Geoinf.* **142**, 104753 (2025).

267. T. A. P. West, E. Vidal, F. E. Putz, Forest biomass recovery after conventional and reduced-impact logging in Amazonian Brazil. *For. Ecol. Manage.* **314**, 59–63 (2014).

268. C. E. Wheeler, P. A. Omeja, C. A. Chapman, M. Glipin, C. Tumwesigye, S. L. Lewis, Carbon sequestration and biodiversity following 18 years of active tropical forest restoration. *For. Ecol. Manage.* **373**, 44–55 (2016).

269. D. J. Woodbury, H. Jayawickrama, M. P. Martin, S. Ediriweera, M. S. Ashton, Land tenure and human disturbance influence the current distribution of aboveground biomass in Sri Lankan rainforest fragments. *For. Ecol. Manage.* **572**, 122285 (2024).

270. D. J. Zarin, E. A. Davidson, E. Brondizio, I. C. G. Vieira, T. Sá, T. Feldpausch, E. A. G. Schuur, R. Mesquita, E. Moran, P. Delamonica, M. J. Ducey, G. C. Hurtt, C. Salimon, M. Denich, Legacy of fire slows carbon accumulation in Amazonian forest regrowth. *Front. Ecol. Environ.* **3**, 365–369 (2005).

271. Z. Zhao, W. Li, P. Ciais, M. Santoro, O. Cartus, S. Peng, Y. Yin, C. Yue, H. Yang, L. Yu, L. Zhu, J. Wang, Fire enhances forest degradation within forest edge zones in Africa. *Nat. Geosci.* **14**, 479–483 (2021).

272. D. Zuleta, A. Duque, D. Cardenas, H. C. Muller-Landau, S. J. Davies, Drought-induced mortality patterns and rapid biomass recovery in a terra firme forest in the Colombian Amazon. *Ecology* **98**, 2538–2546 (2017).
